# Mechanisms of interactions between lung‐origin telocytes and mesenchymal stem cells to treat experimental acute lung injury

**DOI:** 10.1002/ctm2.231

**Published:** 2020-12-08

**Authors:** Ding Zhang, Dongli Song, Lin Shi, Xiaoru Sun, Yonghua Zheng, Yiming Zeng, Xiangdong Wang

**Affiliations:** ^1^ Zhongshan Hospital Institute of Clinical Science Zhongshan Hospital Shanghai Medical College Fudan University Shanghai China; ^2^ Department of Pulmonary and Critical Care Medicine Huashan Hospital Fudan University Shanghai China; ^3^ Department of Respiratory Medicine Shanghai Jinshan Tinglin Hospital Shanghai China; ^4^ Department of Pulmonary and Critical Care Medicine Clinical Center for Molecular Diagnosis and Therapy The Second Affiliated Hospital of Fujian Medical University Quanzhou Fujian Province China

**Keywords:** acute lung injury, co‐transplantation, mesenchymal stem cells, telocyte

## Abstract

Acute lung injury is a serious form and major cause of patient death and still needs efficient therapies. The present study evidenced that co‐transplantation of mesenchymal stem cells (MSCs) and telocytes (TCs) improved the severity of experimental lung tissue inflammation, edema, and injury, where TCs increased MSCs migration into the lung and the capacity of MSCs proliferation and movement. Of molecular mechanisms, Osteopontin‐dominant networks were active in MSCs and TCs, and might play supportive and nutrimental roles in the interaction between MSCs and TCs, especially activated TCs by lipopolysaccharide. The interaction between epidermal growth factor and its receptor from MSCs and TCs could play critical roles in communications between MSCs and TCs, responsible for MSCs proliferation and movement, especially after inflammatory activation. Our studies provide the evidence that TCs possess nutrimental and supportive roles in implanted MSCs, and co‐transplantation of MSCs and TCs can be a new alternative in the therapy of acute lung injury.

## Graphical abstract

1. TCs can accelerate the migration of MSCs from the intraperitoneal space into injured lung tissues through intercellular signals and/or cell‐cell interaction.
2. Pre‐activated MSCs and TCs with LPS show more efficient in cell‐cell communication by OPN‐ or EGFR‐dominated signal pathways

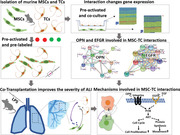

## 1 INTRODUCTION

The acute respiratory distress syndrome (ARDS), a severe type of acute lung injury (ALI), still contributes to high morbidity and mortality in critically ill patients. ARDS/ALI‐associated mortality remains more than 50% due to the lack of effective therapies, despite the improvement of supportive cares.^1^ Pathological changes of ALI mainly include lung tissue inflammation characterized by leukocyte influx and activation, endothelial barrier dysfunction by plasma exudation and edema, and tissue injury by compromised alveolar–capillary barrier, gas exchange, or structure.^2^ There are several challenges in the development of efficient therapies for ALI/ARDS, e.g. unexpected occurrence, acute and rapid progression, uncontrolled severity, or complex mechanisms with the involvement of multi‐cells, mediators, and systems.

Mesenchymal stem cells (MSCs) were suggested as an alternative of potential therapies for ALI to prevent acute lung inflammation, tissue edema, and injury, although there are still a large number of obstacles to be overcome.^3^ Our previous studies initially demonstrated that co‐transplantation of dominant cells with supporting cells could increase therapeutic effects of cells immediately after the induction of acute organ failure.^4^ Transplanted cells need a period to be vascularized, nourished, oriented, or attracted into the location of tissue injury to start biological functions by inflammatory mediators/cells, nutrient factors from supporting cells, or direct interactions between cells. Clinical application of stem cells, recently started in patients with moderate‐to‐severe ARDS using low, intermediate, or high doses of MSCs,^5^ demonstrated that patients could develop the tolerance against the intravenous infusion of allogeneic, bone marrow‐derived human MSCs. Thus, there is an urgent need to further improve applications of MSCs in patients with ARDS.

Telocytes (TCs) were coined and suggested as a type of interstitial cells with podoms, podomers, and long telopodes (Tp).^6^ The profiles of gene expression of primary lung TCs were about 90% similar between 5 and 10 days after cell culture, different from those of pulmonary MSCs, alveolar type II cells, fibroblasts, airway basal cells, proximal airway epithelial cells, CD8^+^ T cells from lungs, or CD8^+^ T cells from bronchial lymph nodes.^7^ Gene expression profiles in chromosomes 17 and 18 of TCs showed that TCs could play a complex role in the maintenance of immune homoeostasis, immune surveillance, cell proliferation and differentiation, and tissue regeneration.^8^ TCs were located in many organs /tissues, especially under airway epithelial cells and interstitial tissues of lungs, and connected with multi‐cells for cell‐cell communication through telopode‐formed networks^9^ and with stem cells within niches in the heart^10^ and lung.^11^ TCs could have close communication among cells within the tissue and augmented stem cell proliferation associated with epithelial renewal by regulating Wnt signaling.^12^

The present study first proved the therapeutic effects of intraperitoneal co‐transplantation of MSCs and TCs in ALI induced by an intratracheal instillation of lipopolysaccharide (LPS). We defined morphological features and phenomes of isolated and cultured TCs from lungs and investigated how TCs supported and attracted MSCs migration and distribution into the injured lung in animals with or without ALI. Therapeutic effects of co‐transplantation were furthermore evaluated in ALI after intraperitoneal injection with cells or conditioned medium, for example, MSCs, TCs, or both at high or low doses, or the corresponding medium at high or low doses. According to the principle of “two‐hit” model,^13^ we pre‐activated MSCs, TCs, or both with LPS for 12 h as the first hit and investigated roles of activated TCs in migration and proliferation of activated or non‐activated MSCs after LPS stimulation as the second hit. Molecular mechanisms of cell‐cell interaction and communication were investigated by profiling gene expressions of MSCs or TCs after the co‐culture, where regulatory roles of osteopontin (OPN)‐dominated signal pathways and epidermal growth factor (EGF)‐receptor (EGFR) signaling in TCs‐MSCs interaction were studied by monoclonal antibody, gene knockdown, or specific inhibitor. The present study especially focused on critical and supportive roles of TCs in the improvement of migration of MSCs into the lung, repair of lung tissue, and acceleration of MSCs capacity in ALI.

## 2 METHODS

### 2.1 Animals

The animal experiments of the present study were approved by Ethical Committee of Zhongshan Hospital Fudan University. 6–8 weeks Female C57BL /6J mice with the weight of 20–25 g and 6–8 weeks old, were provided by Animal Facility, Zhongshan Hospital Institute of Clinical Science, Fudan University. LPS (Product Number L9143) was originated and phenol‐extracted from serotype 10 Pseudomonas Aeruginosa (Sigma‐Aldrich, St. Louis, MO, USA).

### 2.2 Cell preparation and culture

Lung TCs were isolated from lung tissues of 6–8 weeks C57BL /6J mice and cultured.^11^, ^14^ Briefly, the lung tissues were separated after mice were anaesthetized and cut into small pieces in neutralizing digestive fluid after digestion with collagenases. The solution was filtered with 40μm cell strainer and centrifuged at 400 g for 5 min. Cells were resuspend in culture dishes and cultured for cell adhesion. The supernatant with cells was moved into new culture dishes. Cells were distributed at a density of 10^5^ cells/cm^2^ and maintained at 37°C in a humidified atmosphere (5% CO_2_) until becoming 80% confluent. Culture medium was changed every 48 h during TCs were expanded and the passages 6–8 were used for cell transplantation. MSCs were obtained, thawed, and expanded from bone marrow of 6–8 weeks C57BL/6J mice.^15^ In briefly, the bone marrow was washed repeatedly for the single‐cell suspension and harvested fluid was centrifuged at 500 × *g* for additional 5 min to acquire the cell mass. After washing thrice, the cells were resuspended and cultured with DMEM/F12 medium, containing 10% fetal bovine serum (FBS) and 1% penicillin‐streptomycin in cell incubator. Anti‐Sca‐1 antibodies (E13–161.7), anti‐CD34 (RAM34), anti‐CD31 (MEC13.3), anti‐VCAM‐1 (429 [MVCA‐M.A]), anti‐CD11b (M1/70), and anti‐CD45R (RA3‐6B2) and non‐specific isotype IgG as controls (BD Biosciences, NJ) were used for cellular surface labeling, and analyzed by BD FACS Aria III flow cytometer (BD).

### 2.3 MSCs‐ and TCs‐conditioned medium

TCs was confluent at 80‐90% after about 10 days to reach 5 × 10^6^ MSCs, TCs, or both at 1:1 were cultured to about 80% confluences for 24 h after stimulation with or without LPS. After media and cells were collected separately, cells were rinsed thrice and cultured in serum‐free DMEM/F12 for 24 h. The above‐conditioned medium was removed and cellular debris was removed using a 50‐μm filter.

### 2.4 Induction of acute lung injury

Mice were anesthetized with Isoflurane in a plastic box with controlled ventilation and put in a supine position of head‐up on the board tilted at 30°. LPS at 1000 μg/mL was intratracheally administered to mice at a volume of 1 mL/kg mice body weight (BW).^16^ Animals for controls received the same manipulations and volume of PBS at 1 mL/kg BW. Mice remained in the position until regaining of consciousness and then had access to food and water ad libitum. All animals were terminated by the overdose of anesthesia 24 h after the intratracheal instillation of LPS or PBS. The blood was collected from the artery, and then cells and plasma were separated and preserved in ‐80°C. The lung was inflated with 4% paraformaldehyde under the pressure of 20 cm H_2_O and fixed for histopathology analysis.

### 2.5 Distribution of implanted MSCs and TCs

To define the localization and number of implanted cells, MSCs and TCs were labeled with a red fluorescent dye (PKH26 Cell Linker Mini Kit for General Cell Membrane Labeling, MINI26 kit, Sigma) and a green fluorescent dye (CFSE, 21888, Sigma), respectively. The efficacy and viability of labeling were > 98% and > 96%, respectively, evaluated by trypan blue exclusion. C57BL/6J mice were randomized into six groups with the intratracheal administration of PBS or LPS at the volume of 1 mL/kg body weight 1 h before the intraperitoneal injection of 10^6^ MSCs alone (n = 18), TCs alone (n = 18), or MSCs combined with TCs (n = 18). Mice were then terminated at 24, 48, or 72 h after the intraperitoneal administration of cells (n = 6 per time point).

Cells were isolated from the left lung using dispase^®^ II enzyme at 3.6 U/mL (Roche Mannheim, Germany).^17^ The cell number per lung was determined by using a hemocytometer. Cells labeled with fluorescence were isolated and analyzed by BD FACSAria III flow cytometer (BD) with 50 000 events per sample. The number of donor cells per lung tissue was calculated as the formula: (Isolated cell number per lung × [identified labeled donor cells/50 000]). The percentage of donor cell number per lung tissue/10^6^ was recorded. The right lung was fixed using 4% paraformaldehyde, and the left lung was infiltrated with PBS containing 30% sucrose under the pressure of 20 cm H_2_O and then fresh‐frozen in liquid nitrogen with Tissue‐Tek OCT Compound (Sakura Finetek, Torrance, CA, USA). Sections of five μm and 50 μm thick were stored at −80°C before the analysis and then fixed in ice‐cold acetone for 10 min for use. Nuclei was stained with 4′, 6′ ‐diamidino‐2‐phenylindole dihydrochloride hydrate (Sigma‐Aldrich). Images were recorded with a TCS SL laser scanning confocal microscope (Leica Microsystems, Mannheim, Germany).

### 2.6 Experimental design of cell co‐transplantation

The therapeutic effects of cell co‐transplantation were evaluated by either MSCs or TCs alone or together at low and high concentrations (n = 80 animals, 8 animals per group) after a pilot study. Animals without any manipulation were used as negative controls, intraperitoneally injected with vehicle at the volume of 0.5 mL per animal for 24 h, and intratracheally administered with PBS at 1 mL/kg BW as the manipulation controls, or with LPS at 1 mg/mL/kg BW as positive controls (ALI). Animals were intratracheally challenged with LPS 24 h after the intraperitoneal injection with vehicle at the volume of 0.5 ml per animal, with MSCs at 10^6^ or 5 × 10^6^, TCs at 10^6^ or 5 × 10^6^, or MSCs combined with TCs at 10^6^ or 5 × 10^6^ each, respectively. Potential effects of mediators produced from cells were investigated by an intraperitoneal injection of the conditioned medium. Another 80 animals were used, including native controls, manipulation controls, or positive controls, or groups were intraperitoneally injected with 0.5 mL MSCs‐conditioned medium at 25% or 100% of the origin, TC‐conditioned medium at 25% or 100%, or MSCs/TCs‐conditioned medium at 25% or 100% for 24 h, respectively, followed by the intratracheal administration with LPS.

### 2.7 Pulmonary inflammation and endothelial barrier dysfunction

The lung was intratracheally washed with sterile PBS under the pressure of 20 cm H_2_O. The bronchoalveolar lavage fluid (BALF) was collected into tubes on ice and the volume was recorded. The BALF was centrifuged at 1200 rpm for 5 min and the supernatant was stored at ‐80°C for further analyses. Pulmonary endothelial barrier dysfunction was measured by the plasma exudation from the peripheral blood into the alveolar space. Total protein in BALF were measured with an enhanced BCA Protein Assay Kit (P0010S, Beyotime, Shanghai, China) and albumin with a murine‐specific albumin ELISA kit (AB108792, Abcam, Cambridge, MA) at the detection limit of 1.5 ng/mL, respectively. The cells were harvested from BALF and re‐suspended in 0.25 mL PBS, and the number of neutrophils and white blood cells was measured using Auto Hematology Analyzer (BC‐5300 VetTM, Mindray, China). Cytokine levels of interleukin (IL)‐6 and IL‐1β in BALF or plasma were evaluated with cytokine‐specific Quantizing ELISA kits (R&D Systems).

### 2.8 Evaluation of lung tissue injury

Lung tissue damage and injury induced by LPS were evaluated by pathological scoring. The lungs from additional animals in each group (n = 6) were washed with PBS under the pressure of 20 cm H_2_O, fixed with 4% paraformaldehyde, embedded in paraffin, and cut into sections of 5 μm thick. After stained with hematoxylin and eosin, photos were obtained with a BX51 microscope DP71 camera (Olympus, Toyko, Japan). Twenty random high‐power fields (X400) were independently scored. Images were evaluated for the lung injury score evaluation as reported previously.^18^

### 2.9 Gene expression profiles of MSCs‐TCs interaction

To define alterations of gene expression profiles during MSCs‐TCs interaction, gene expression profiles were evaluated in co‐culture of MSCs and TCs at the concentration of 10^5^, as compared with MSCs or TCs alone. We also investigated potential effects of activated TCs or MSCs stimulated with LPS on MSCs or TCs, as compared with MSCs or TCs alone, or co‐culture of TCs and MSCs without activation. Gene expression profiles were measured as previously described.^7^ Briefly, total RNAs were isolated, amplified, labeled by One‐Color Low Input Quick Amp Labeling Kit (Agilent technologies, Santa Clara, CA, US), and purified using RNeasy mini kit (QIAGEN, GmBH, Germany). Each slide was hybridized using Gene Expression Hybridization Kit (Agilent) in Hybridization Oven (Agilent), washed in dishes with Gene Expression Wash Buffer Kit (Agilent) for 16 h, and then scanned using Agilent Microarray Scanner equipped with default settings. Data were measured with Feature Extraction software 10.7 and raw data were normalized with Quantile algorithm, Gene Spring Software 11.0 (Agilent).

### 2.10 Validation of selected OPN or EGF roles

Two potential driven factors OPN and EGF were selected for the further investigation to understand potential mechanisms of MSCs‐TCs interaction. MSCs or TCs were plated into 12 well plate 24 hrs and then transfected with RNA interference OPN siRNA (sc‐36130, Santa Cruz Co. Ltd, USA), EGF siRNA (sc‐39417, Santa Cruz Co. Ltd), and the fluorescein‐conjugated control siRNAs (sc‐36869, Santa Cruz Co. Ltd) using lipofectamine 2000 (Invitrogen, CA). Knockdown of OPN or EGF was identified by quantitative PCR and western blot 48 h after expression of the siRNAs. β‐actin was used as an internal control. OPN or EGF‐positive or negative MSCs or TCs at the concentration of 10^5^ cells/well were cultured alone or co‐cultured with or without the challenge of LPS at doses of 10, 100, or 1000 ng/mL for 4, 8, 24, or 48 h, respectively. MSCs or TCs were detached and collected with Trizol (Invitrogen, Carlsbad, USA) for RNA isolation. Total RNA was isolated with TRIZOL(Invitrogen) and measured with NanoDrop One (Thermo) at 260 nm. RNA was reversed and transcribed to cDNA using the Super‐Script First‐strand Synthesis System (Invitrogen). Quantitative RT‐PCR was carried out with the two‐stage program parameters using an ABI 7000PCR instrument (Eppendorf, Hamburg, Germany), as follows: at 95°C for 1 min, then at 95°C for 30 s, and at 60°C for 40 cycles of 5 s. The sequences of the primer sets are as follows: mouse OPN: 5′‐GAGGAAACCAGCCAAGGTAA‐3′ (forward [F]) and 5′‐GCAAATCACTGCCAATCTCA ‐3′(reverse [R]); mouse EGF: 5′‐GGCAGACAGAGCCAGTTCA‐3′ (F) and 5′‐ AGCAGTGATTAGCCGTGGAA ‐3′ (R); mouse Connexin 43: 5′‐GAACACGGCAAGGTGAAGAT‐3′(F) and 5′‐GAGCGAGAGACACCAAGGAC‐3′(R); and mouse glyceraldehyde‐3‐phosphate dehydro‐genase (GAPDH): 5′‐GGTGAAGGTCGGTGTGAACG‐3′(F) and 5′‐CTCGCTCCTGGAAGATGGTG‐3′(R). Dissociation reaction plots were examined for the confirmation of specificity of the amplification product. Each sample had six wells and each well was tested in triplicate.

### 2.11 Western blotting

Cells were detached in 1 mM EDTA solution and then added lysing buffer (Sigma–Aldrich). Proteins were boiled for 5 min with β‐mercaptoethanol, and separated by electrophoresis in 10% (wt/vol) SDS‐polyacrylamide gel. Proteins were loaded in each lane and transferred onto polyvinylidene difluoride (PVDF) membrane. The membrane with proteins were incubated with TBST buffer (10 mM Tris /HCl [pH 8.3], 0.05% Tween‐20, and 150 mM NaCl) containing 5% (wt/vol) milk powder for 60 min at room temperature to block nonspecific binding. Membranes with protein were incubated with the primary antibodies, including anti‐mouse EGF monoclonal antibody (D5, sc‐374255, Santa Cruz Biotechnology Inc., CA, USA), anti‐mouse Connexin 43 (Cx43) monoclonal antibody (D7, sc‐13558) at 4°C overnight, and then mouse or goat anti‐mouse IgG‐HRPs (sc‐2005) as the secondary antibody. Revelation was performed with the ECL plus Western blotting detection kit (Amersham Biosciences, Saclay‐Orsay, France), and Chemocapt software using Chemi‐Smart 2000 (Vilber Lourmat, Marne‐la‐Vallée, France) were used for image acquisition.

### 2.12 Evaluation of cell‐cell interactions

Indirect interactions between MSCs and TCs were measured with the Trans‐Well migration assay. Migration ratios were examined in transwell dishes with the diameter of 6.5 mm and 8‐μm pore filters (Corning Costar, Cambridge, MA, USA). The upper chamber was first coated with 0.1% (wt/vol) bovine gelatin (Sigma‐Aldrich) for 1 h at 37°C and then with MSCs at 5 × 10^4^ cells. The low chamber was coated with TCs at 5 × 10^4^ cells in serum‐free DMEM/F12. The complete culture medium was collected 24 h after MSCs, TCs, or both were challenged with vehicle or LPS at 1000 ng/mL. Cells in upper chambers were washed with pre‐cold PBS for twice, and were stained with May‐Grünwald‐Giemsa. The total number of cells migrated from the upper chamber to the low ones were counted under light microscopy (×200). The influence of external OPN in the interaction between MSCs and TCs on cell migration was also evaluated by neutralizing intracellular OPN with anti‐mouse OPN antibody (AF808, R&D). Concentrations of anti‐OPN antibody used were selected and chosen on basis of our studies.

Direct interactions between MSCs and TCs were assayed by measuring cell bio‐behaviors by Cell‐IQ (Chip‐man, Finland), for example, cell morphology, proliferation, death, differentiation, and movement. MSCs, TCs, or both at 10^4^ cells were placed on 24‐well plates and cultured in an incubator for 24 h. The plates were then incubated in Cell‐IQ incubator after the treatment. Analysis was performed using a freely distributed Image software (McMaster Biophotonics Facility, Hamilton, ON, Canada), with the Manual Tracking plugin supported by Fabrice Cordeliéres (Institut Curie, Orsay, France). The cell bio‐behaviors were examined using a real‐time cell monitoring system equipped in a Cell‐IQ cell‐culturing platform (Chip‐Man Technologies). Images of each visible through a microscope were captured at 60‐minute intervals for 72 h. Total cell number of each image was measured automatically by Cell‐IQ system. Each group contained three wells and four replicate microscopic fields were measured in each. To investigate the connexin 43‐dependent influence of TCs on MSCs proliferation, CFSE‐labeled MSCs at 10^5^ cells per well were directly co‐cultured with PKH‐26 labeled TCs at the ratio of 1:1 or alone in serum‐free DMEM/F12 for 24 h. Cells were harvested and MSCs proliferation was determined by flow cytometry analysis of CFSE fluorescence 24, 48, and 72 h after 1000 ng/mL LPS was added in MSCs or co‐cultured with TCs, with or without the appearance of carbenoxolone disodium (CBX, Sigma, St. Louis, MO, USA), a gap‐junction (connexin 43) inhibitor, at doses of 1, 10, or 100 nM.

### 2.13 Statistical analysis

Values were expressed as means ± SEM. Differences between groups were assessed by one‐way ANOVA with post hoc comparisons and Dunnett's test with statistic software (GraphPad Prism version 6.0). All in vitro experiments were performed in triplicate and repeated three to six times. A value of *P*<.05 was considered statistically significant.

## 3 RESULTS

### 3.1 Isolation and characterization of murine pulmonary TCs

**FIGURE 1:**
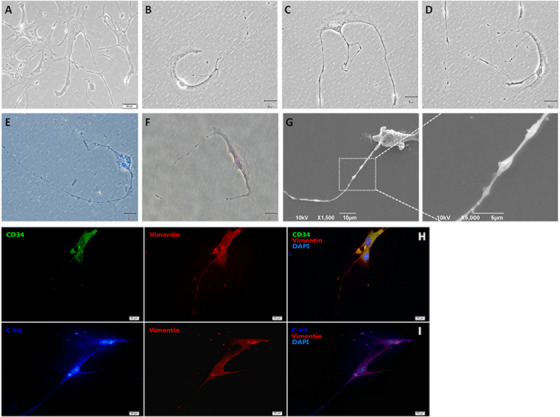
Morphological features of isolated and cultured telocytes from lungs. Multi‐phenotypes of telocytes were detected within 5 days after the culture (A). The long (B), thin and moniliform prolongations named telopodes (C, D) became clearer from 10 days and on. Mitochondria were identified in telocyte bodies (E) and telopodes (F). Telopodes of lung telocytes were detected by scanning electronic microscope (G). Telocytes were positive for CD34, c‐kit and vimentin (H, I) by the staining of immunofluorescence cytochemistry

Of TCs biomarkers,^19^ CD34‐, c‐kit‐, or vimentin‐positive cells and TCs morphological specificities at passages between 6 and 8 were selected and cultured as mouse lung origin TCs, including the extensive cellular body with long and thin prolongations (telopodes) (Figure 1). The long, thin, and moniliform telopodes became clearer from 10 days and on (Figure 1A–D). Dilated portions (podoms) distributed along the telopode (Figure 1E–G) with mitochondria and endoplasmic reticulum by transmission electronic microscopy, and form thin segments (podomers) by phase‐contrast microscopy with methylene blue staining (Figure 1E,F). Telopodes of lung TCs were detected in Figure 1G). Those TCs maintained positive staining of CD34, c‐kit, or vimentin (Figure 1H–I), while murine MSCs were positive for Sca‐1, CD34^+^, or VCAM^+^,^20^ and negative for both monocyte marker (CD11b) and leukocyte marker (CD45R) by flow cytometry (Supplement Figure 1).

### 3.2 Effects of co‐transplantation on cell distributions in lungs

**FIGURE 2:**
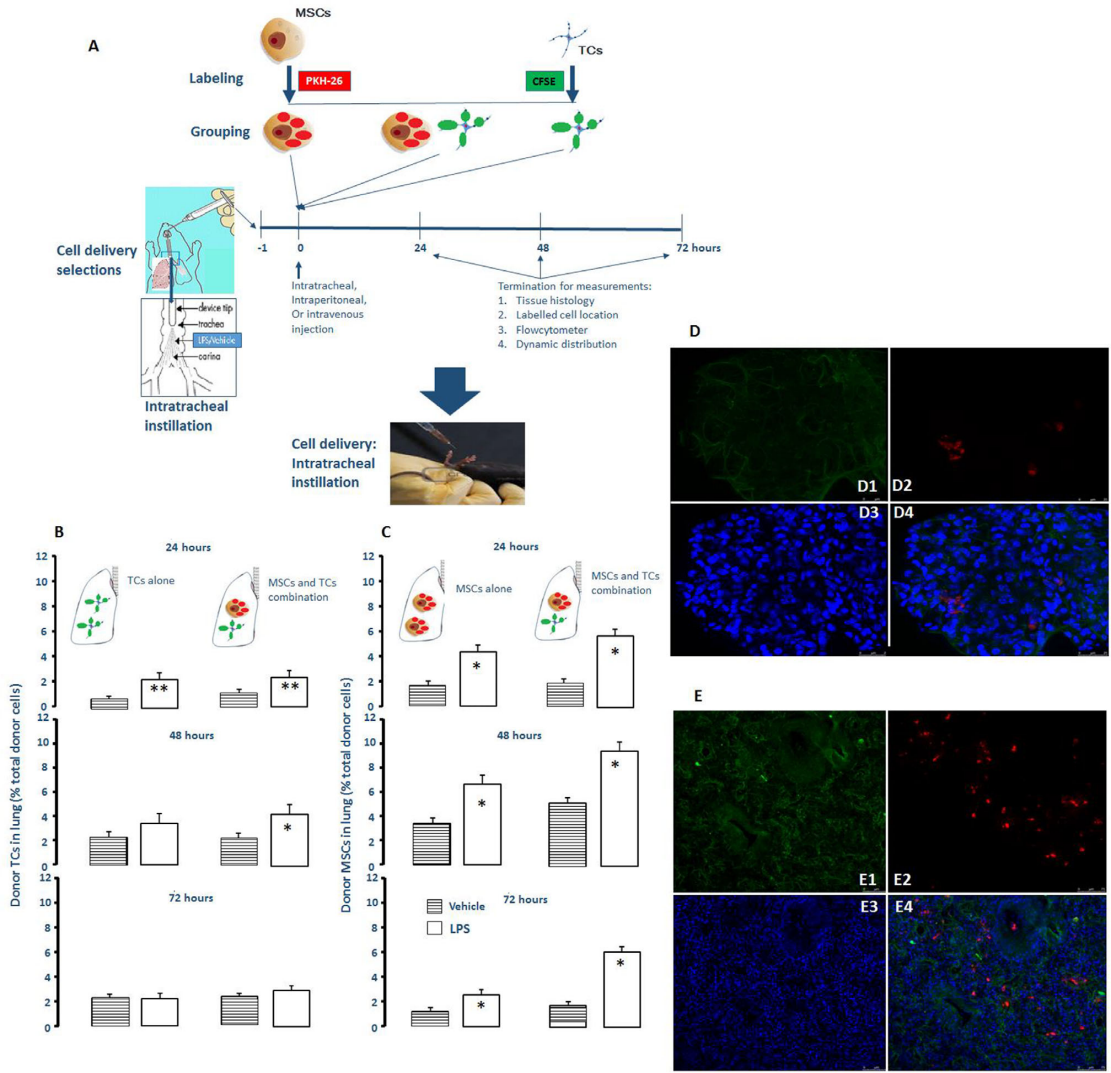
Effects of TCs on MSCs migration and distribution. The intraperitoneal injection was selected as the cell delivery approach after compared with intravenous and intratracheal administration of PKH‐26 labeled MSCs and CFSE labeled TCs in animals with or without LPS‐induced ALI (A). The number of implanted TCs (B) or MSCs (C) to the lung were measured 24, 48, and 72 h after the intraperitoneal injection of TCs, MSCs, or combination in animals with or without the intratracheal challenge of LPS. * and ** stand for *P* values < .05 and .01, respectively, as compared with the correspondent groups without LPS challenge. Labeled MSCs (Red) migrated into lungs and formed small clusters in the normal condition (D2, D4) rather than TCs (D1, D3). Under LPS challenge, MSCs were mainly located in the interstitial and inflammatory area and a few in the lumen of small airways or alveolar space (E2, E4), while migrated TCs were also detected in the lung tissue (Green, E1, E3). Nuclei were stained with DAPI (blue)

Therapeutic effects and distributions of transplanted cells depend upon delivery locations and processes.^21^ To measure the optimal deliver of co‐transplanted MSCs and TCs in ALI,^22^ we analyzed dynamic distributions in lung tissue and retentions of transplanted cells at 24, 48, or 72 h after intratracheal, intravenous, or intraperitoneal deliveries of MSCs, TCs, or both, 4 or 24 h after the intratracheal instillation of LPS or vehicle (Figure 2A). About 60–70% or 30% of animals died within 24 h after intravenous or intratracheal injection of MSCs, TCs, or both (n = 10/group). About 80 % of animals had acute lung inflammation, edema, terminal airway blocking, or alveolar wall damage from 24 h after intratracheal instillation of cells, independent upon injected cell types. LPS‐induced higher migration and retention of labeled TCs in lungs 24 h or 24–48 h after the intraperitoneal injection of TCs or both (Figure 2B), while increased number of labeled MSCs in lungs during 72 h after the intraperitoneal injection of MSCs or both (Figure 2C).

The number of labeled MSCs in animals with both cells significantly higher than those in animals with MSCs, with a peak at 48 h, as compared with that at 24 or 72 h, respectively. The number of labeled MSCs in lungs was significantly higher than that of TCs, especially under LPS challenge, mainly located in the interstitial space and inflammatory area in a small form of cell clusters (Figure 2D), and a few in the lumen of small airways or alveolar space. TCs were mainly located in the interstitial space, and a few gathered with MSCs (Figure 2E). The number of labeled MSCs or TCs in lungs increased maximally 48 h after the transplantation with or without LPS challenge. More than 60% of MSCs disappeared at 72 h after MSCs transplantation, while less than 40% after co‐transplantation at 48 h. To confirm the intact cell with the fluorescence in the lung tissue, labeled TCs or MSCs with nuclear staining and CFSE labeling were detected using laser scanning confocal microscopy. From three‐dimensional merging images, we noticed more clearly the appearance of MSCs or TCs in lungs and co‐appearance of both together.

### 3.3 Effects of co‐transplantation on the severity of ALI

**FIGURE 3:**
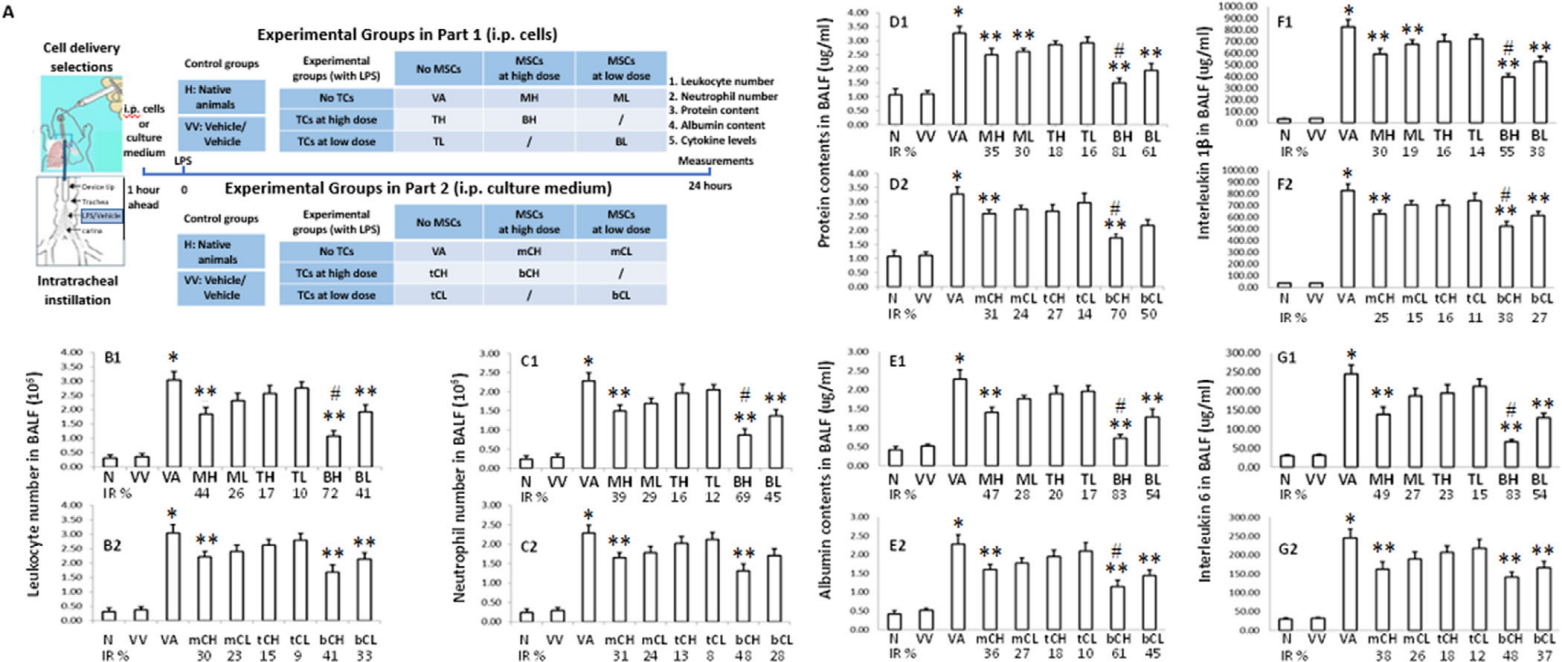
Therapeutic effects of TCs and MSCs co‐transplantation on acute lung injury. Study protocol (A) demonstrated that animals were intraperitoneally injected with cells (B1‐G1) or cell‐cultured medium (B2‐G2) 1 h before intratracheal infusion of LPS and terminated 24 h after LPS challenge. Study 1 included animals without any treats (N), with vehicles (VV), with vehicle and LPS‐induced ALI (VA), with MSCs at high (MH) or low doses (ML) and ALI, with TCs at high (TH) or low doses (TL) and ALI, with both at high (BH) or low doses (BL) and ALI. Study 2 included animals without any treats (N), with vehicles (VV), with vehicle and LPS‐induced ALI (VA), with MSCs medium at high (mCH) or low doses (mCL) and ALI, with TCs medium at high (tCH) or low doses (tCL) and ALI, with both at high (bCH) or low doses (bCL) and ALI. Levels of leukocytes (B), neutrophils (C), total proteins (D), albumin (E), interleukin 1β (F), and interleukin 6 (G) in bronchoalveolar lavage fluid were measured at 24 h. Inhibitory rate (IR %) was calculated as the ratio = (each treat group‐VV)/(VA‐VV) × 100. * and ** stand for *P* values < .05, as compared with animals treated with vehicle and ALI animals, and ^#^ *P* values < .05, as compared with ALI animals treated with MSCs at high dose (n = 10/group)

**FIGURE 4:**
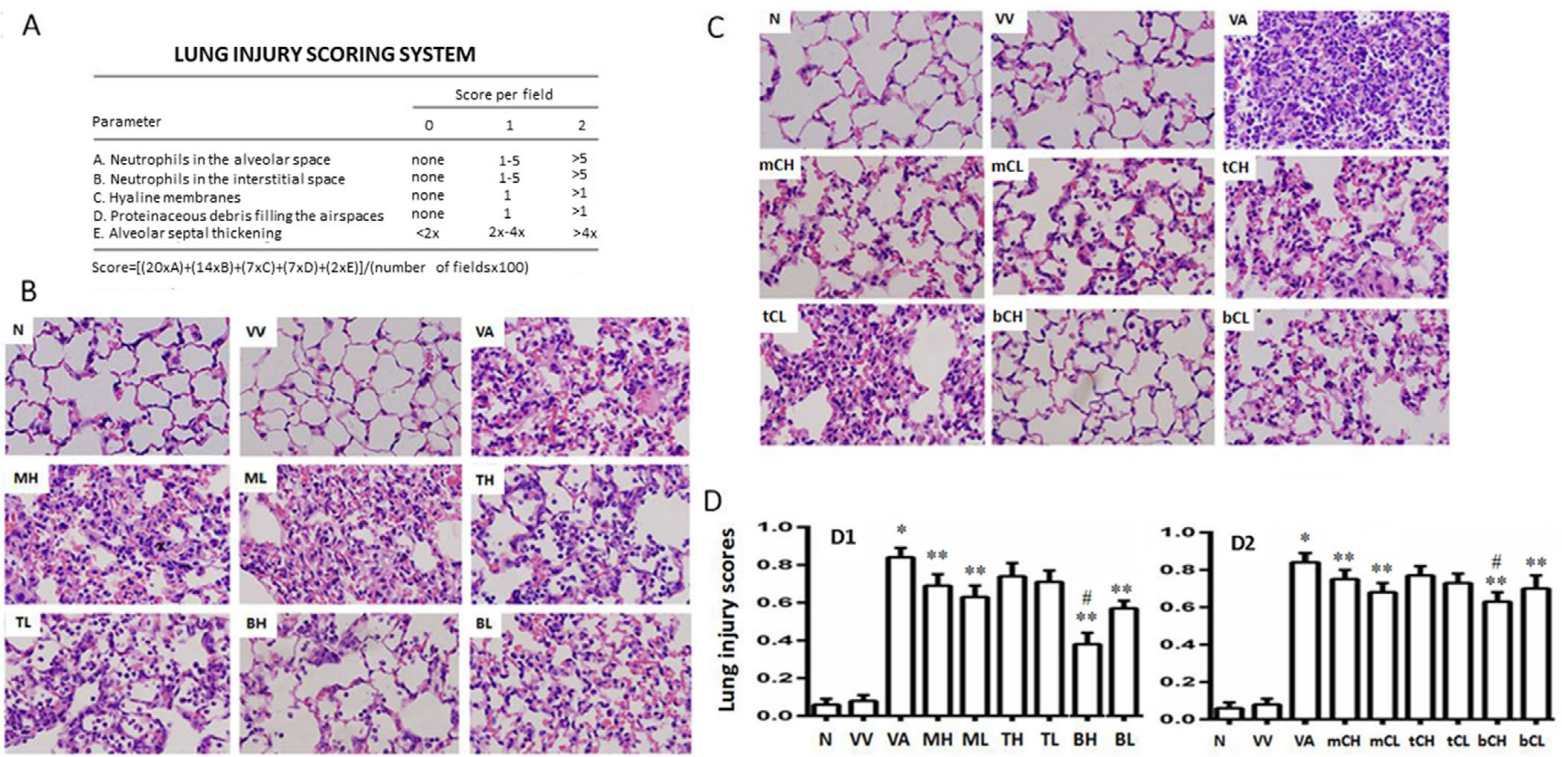
Therapeutic effects of TCs and MSCs co‐transplantation on lung pathological alterations. According to histopathological evaluation score system of acute lung injury (A), Study 1 (B) included animals without any treats (N), with vehicles (VV), with vehicle and LPS‐induced ALI (VA), with MSCs at high (MH) or low doses (ML) and ALI, with TCs at high (TH) or low doses (TL) and ALI, with both at high (BH) or low doses (BL) and ALI. Study 2 (C) included animals without any treats (N), with vehicles (VV), with vehicle and LPS‐induced ALI (VA), with MSCs medium at high (mCH) or low doses (mCL) and ALI, with TCs medium at high (tCH) or low doses (tCL) and ALI, with both at high (bCH) or low doses (bCH) and ALI. Levels of histological scores in each group were presented (D). * and ** stand for *P* values < .05, as compared with animals treated with vehicle and ALI animals, and ^#^ *P* values < .05, as compared with ALI animals treated with MSCs at high dose (n = 10/group). Photomicrographs were obtained with a BX51 microscope DP71 camera (Olympus, Toyko, Japan) with ×40 and 20 μm scale bar

To investigate if the combination of MSCs and TCs may have therapeutic effects through cells per se or cell‐produced mediators, we pretreated animals with LPS‐induced ALI by MSCs, TCs, or both at low or high doses, or by the corresponding conditioned medium at low or high doses, respectively, as explained in Figure 3A. LPS‐induced ALI demonstrated pathological features similar to human ALI /ARDS,^22^ including lung inflammation reflected by leukocyte and neutrophil recruitment (Figure 3B,C), capillary barrier dysfunction by alveolar protein influx (Figure 3D), and plasma leakage (Figure 3E), and over‐production of inflammatory mediators, for example, IL‐1β (Figure 3F) or IL‐6 (Figure 3G), and tissue injury by pathological scores (Figure 4). The transplantation of MSCs prevented 30–40% LPS‐induced recruitment of leukocytes and neutrophils in a dose‐dependent pattern (Figure 3B1 and 3C1), while TCs showed slightly benefit (10‐20%). Co‐transplantation of MSCs and TCs improved 40–70% of leukocyte recruitment, leakage of total proteins (Figure 3D1) and albumin (Figure 3E1), or over‐production of IL‐1β (Figure 3F1) or IL‐6 (Figure 3G1), significantly better than either alone. To evaluate general therapeutic effects of co‐transplantation on lung injury and inflammation, we pooled inhibitory rates of those 6 measurements and found that co‐transplantation at low and high doses improved 49 and 74% of ALI severity, respectively, obviously higher than MSCs (27 and 40%) or TCs (14 and 19%) alone.

To investigate if nutritional factors produced from MSCs, TCs, or both may play the dominate roles in cell therapies as suggested previously,^23^ we injected the conditioned mediums harvested from MSCs, TCs, or both culture at low and high doses into the peritoneal cavity in mice as scheduled and performed in cell implantation. Among leukocyte (Figure 3B2) and neutrophil (Figure 3C2) recruitment, total protein (Figure 3D2) and albumin (Figure 3E2) leakage, and IL‐1β (Figure 3F2) and IL‐6 (Figure 3G2), the combination‐conditioned mediums at low or high doses could improve 45–50% or 60–70% of total protein or albumin leakage in ALI, respectively, superior to the mediums from MSCs or TCs. Conditioned medium from MSCs alone could restore sodium transport and preserve epithelial permeability probably through the regulation of growth factors.^24^ Our data indicates that the mixture or co‐appearance of some elements from MSCs and TCs may be necessary in restoration of endothelial barrier permeability.

Therapeutic effects of MSCs‐TCs co‐transplantation were further evidenced by pathological characteristics and scores of compromised lungs induced by LPS, according to the scoring systems in Figure 4A.^25^ MSC implantation improved lung interstitial inflammation, which still occurred in animals with TCs (Figure 4B). Conditioned medium of MSCs and TCs combination mainly reduced lung tissue edema and alveolar plasma exudation (Figure 4C). Lung injury scores demonstrated that the co‐transplantation at high doses prevented about 50–60% LPS‐induced lung injury, while MSCs alone could inhibited about 15–25% (Figure 4D1), like levels in conditioned medium of MSCs and TCs combination (Figure 4D2).

### 3.4 Effects of LPS‐activated TCs in regulation of MSCs migration

**FIGURE 5:**
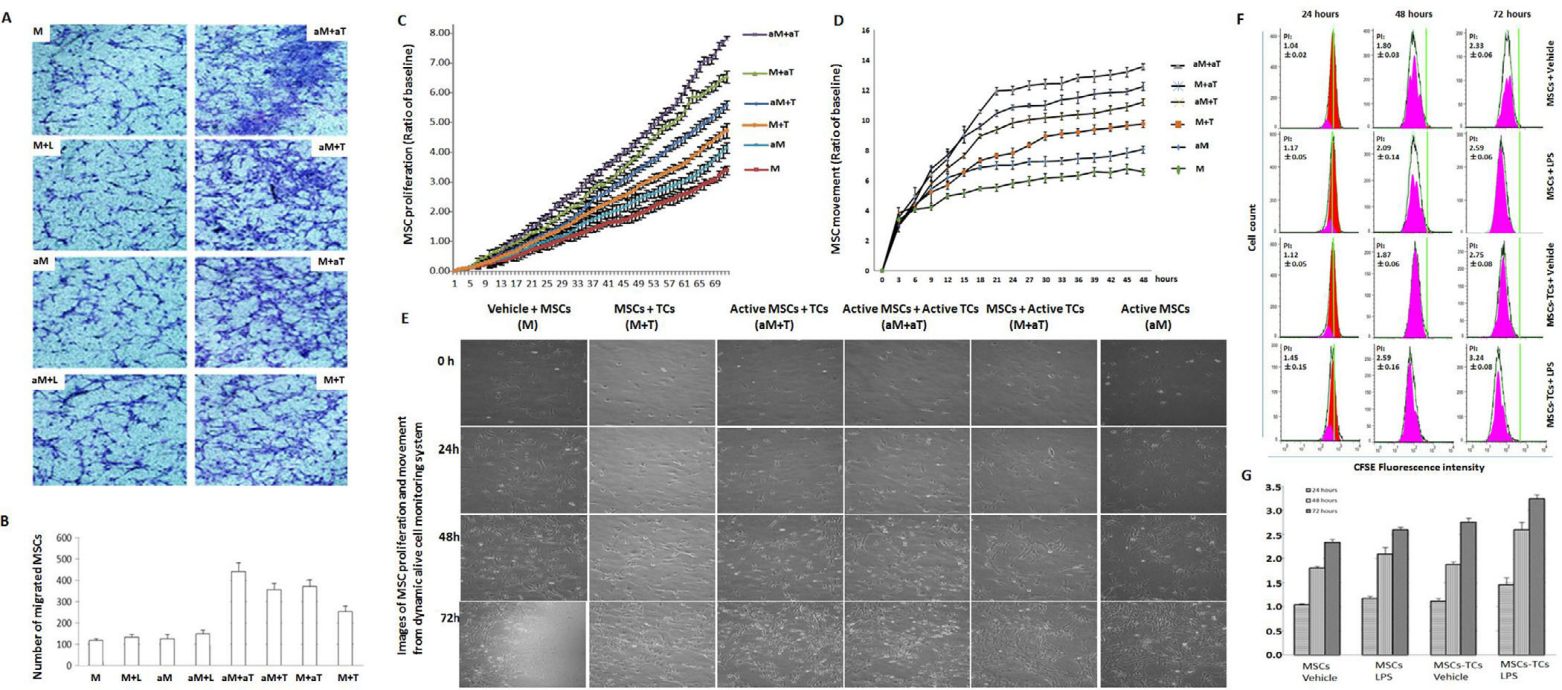
Effects of LPS‐activated TCs on migration and proliferation capacity of MSCs. Histo‐photographs (A) and number (B) of migrated MSCs from the upper chamber to the lower in the Transwell system 24 h after co‐culture MSCs pre‐activated by LPS (L) or vehicle with TCs pre‐activated by LPS or vehicle. Dynamic proliferation (C) and movement (D) as well as images (E) of MSCs preactivated and stimulated by vehicle (M), preactivated by vehicle and stimulated by LPS (M+L), preactivated by LPS and stimulated by (aM+L), after co‐culture of MSCs with TCs preactivated and stimulated by LPS (aM+aT), after co‐culture of MSCs preactivated by LPS with TCs preactivated by vehicle and stimulated by LPS (aM+T), after co‐culture of TCs pre‐activation by LPS with MSCs with vehicle and stimulated by LPS (M+aT), or after co‐culture of MSCs with TCs without pre‐activation and stimulated by LPS (M+T). Promoting effects of TCs on MSCs proliferation during LPS activation were evaluated by cell counting kit‐8 using flow cytometer (F) and cell counts (G), 24, 48, and 72 h after MSCs or TCs‐co‐cultured MSCs challenged with vehicle or LPS

To investigate whether TCs can increase the migration of MSCs from the peritoneal cavity to the lung, we observed the indirect interaction between MSCs and TCs by measuring migration of MSCs pre‐activated with LPS or vehicle from the upper chamber to the low one where TCs pre‐activated with LPS or vehicle. The better efficacy of MSCs and TCs may depend upon cell migration and specific homing towards the site where the cells are needed.^26^ To investigate whether TCs can increase MSCs capacities of migration and infiltration, we measured migrated numbers of MSCs from upper chamber to lower one where TCs were located, in conditions of pre‐activated MSCs or TCs cultured alone or co‐culture. Co‐cultured TCs could increase MSCs capacities of migration and infiltration, especially when either TCs or MSCs were pre‐activated with LPS (Figure 5A,B). The interaction of pre‐activated both showed maximal effects mainly through factors produced from TCs that did not contact with MSCs directly.

Effects of LPS‐activated TCs or MSCs on dynamic proliferation and movement of MSCs were furthermore evaluated during 48 hrs using the dynamic monitoring system of cell behaviors. To assess direct effects of TCs on MSCs, we mixed and cultured MSCs alone or with TCs with or without pre‐activation in the system (Figure 5E). The direct interaction between TCs and MSCs also increased MSCs proliferation (Figure 5C) and movement (Figure 5D), and became more obvious when both were pre‐activated with LPS. TCs could promote the proliferation of MSCs (Figure 5F,G), especially when both was activated.

### 3.5 Altered gene expression profiles of MSCs‐TCs interactions

**FIGURE 6:**
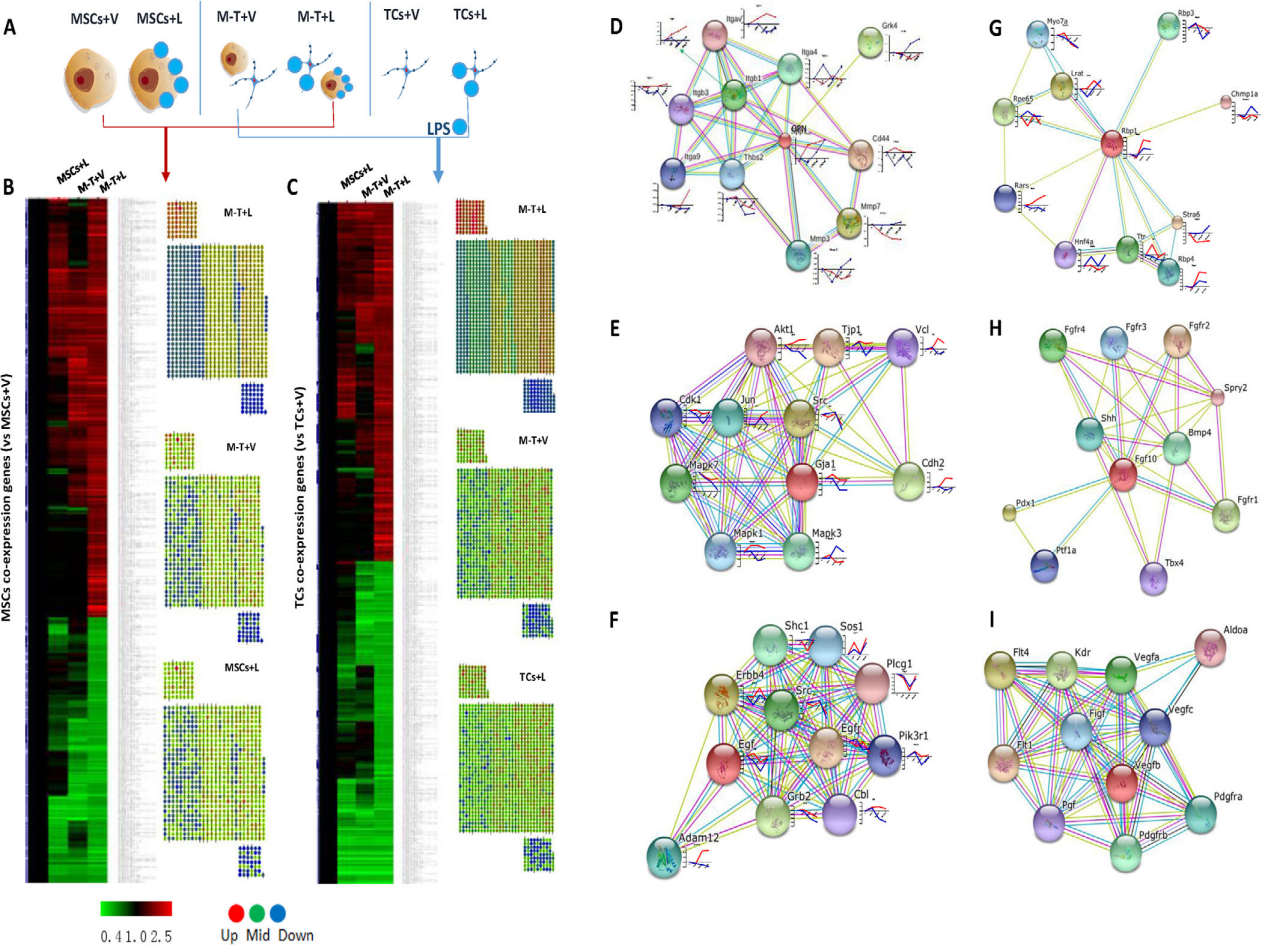
Gene expression profiles of MSCs or TCs after the interaction. MSCs or TCs were cultured alone or co‐cultured together with or without LPS challenge for 24 h and then terminated for measuring gene expression (A). Different gene expressions and interaction‐influenced genes of MSCs (B) or TCs (C) harvested from the co‐culture of MSCs and TCs with vehicle (M‐T‐V) or LPS (M‐T‐L), MSCs alone with vehicle (MSCs+V) or LPS (M‐L), or TCs alone with vehicle (TCs+V) or LPS (TCs+L) were evaluated and presented as compared with gene expression profiles of MSCs or TCs with vehicle. Interaction networks of key elements of MSCs or TCs with or without the co‐culture were evaluated, of which some key networks become active and different, for example, OPN‐ (D), Gja1‐ (E), EGF‐ (F), Rbp1‐ (G), FGF10‐ (H), or VEGF‐dominated networks (I), with alterations of individual elements

TCs could benefit MSCs function both directly and indirectly in either pre‐activated or activated condition, although the exact mechanism remains unclear. To investigate the interaction between MSCs and TCs, we measured gene expression profiles of MSCs or TCs, respectively, after culture of either or both challenged with LPS (Figure 6A). The interaction of TCs and MSCs altered gene expression profiles of MSCs (Table S1A) or TCs (Table S2A), which became more different after LPS challenge and was evidenced by the expression and distribution of co‐expressed genes in MSCs (Figure 6B) or TCs (Figure 6C). To overcome low coverage and high false‐positive rates or high false‐negative rates, molecular networks (or modules) were suggested to be a more robust form to characterize diseases than individual molecules.^27^ Of multi‐networks, we showed elements of OPN (Figure 6D), gap junction protein alpha 1 (Gja1, Figure 6E), epidermal growth factor (EGF, Figure 6F), or retinol binding protein 1 (Rbp1, Figure 6G), fibroblast growth factor 10 (FGF10, Figure 6H), or vascular endothelial growth factor (VEGF)‐dominated networks (Figure 6I). We also analyzed sub‐network elements of each network, interactions, and alterations of those six dominant gene family members, as detailed in Figure S2.

### 3.6 Roles of OPN in MSCs‐TCs communication

**FIGURE 7:**
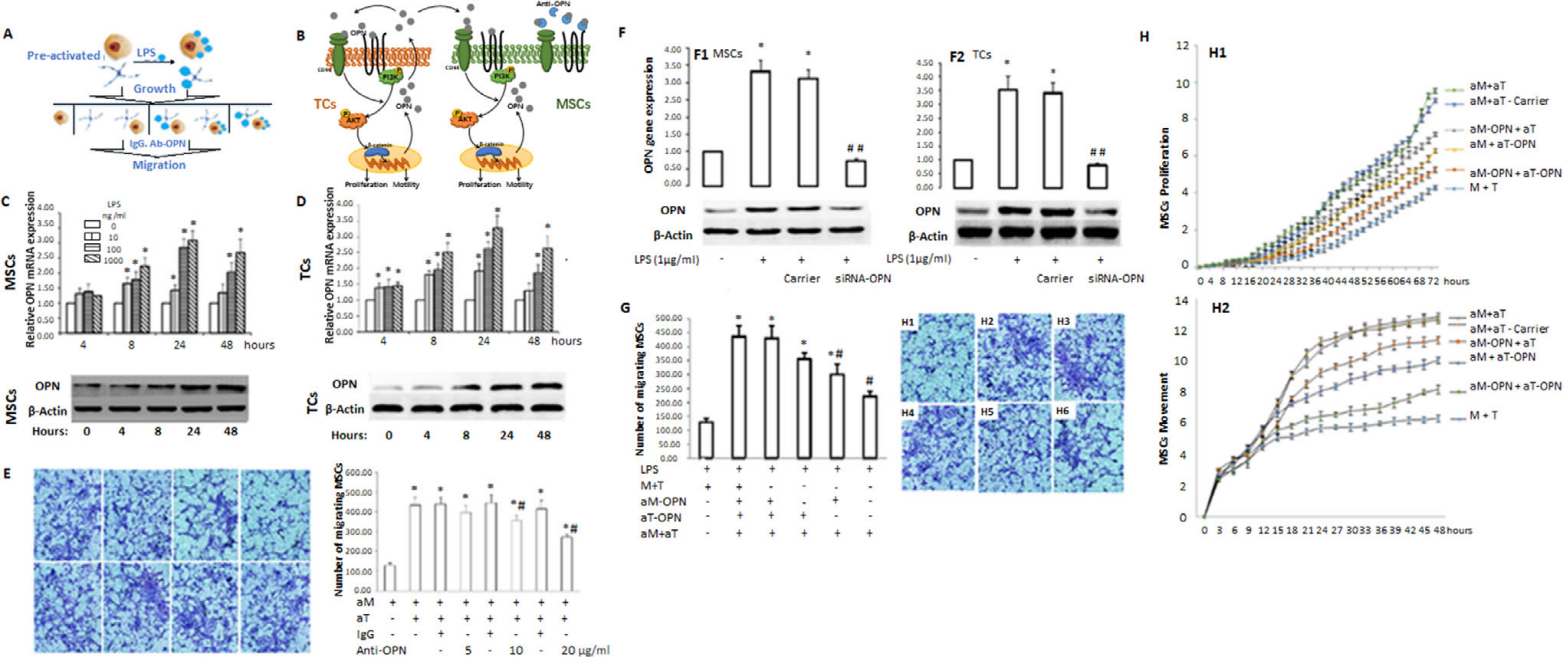
Potential roles of OPN in the interaction between TCs and MSCs. MSCs or TCs were pre‐activated with LPS or vehicle and then cultured alone or together (A). The principle of the present study is to evaluate potential effects of TCs on MSCs through TCs‐produced OPN as paracrine ways, MSCs‐produced OPN as autocrine ways, or external OPN using monoclonal antibody against OPN (B). The influence of inflammation in mRNA and protein expression of OPN was evaluated in MSCs (C) or TCs (D) 4, 8, 24, and 48 h after LPS stimulation at different doses. Effects of secreted OPN on MSCs migration was assessed (E) in MSCs preactivated by LPS (aM), TCs preactivated by LPS (aT), and both co‐culture by the mono‐antibody against OPN (Anti‐OPN) or nonspecific antibody (IgG) at different doses. * and ^#^ stand for *P* values < .05, as compared with aM group and aM+aT without IgG or Anti‐OPN, respectively. We screened and selected the high efficacy of siRNA against OPN genes (siRNA‐OPN) to inhibit OPN mRNA and protein expression in MSCs (F1) or TCs (F2) after LPS stimulation. * and ^##^ stands for *P* values < .05, as compared with cells with vehicle and cells stimulated by LPS and treated with siRNA carrier (Carrier), respectively. We further evaluated the migration capacity of co‐cultured MSCs and TCs without pre‐activation (M+T), MSCs*^OPN‐^* preactivated by LPS (aM‐OPN), TCs*^OPN‐^* preactivated by LPS (aT‐OPN), MSCs preactivated by LPS (aM), or TCs preactivated by LPS (aT) 24 h after LPS challenge (G). * and ^#^ stand for *P* values < .05, as compared with as compared with vehicle and animals treated with vehicle and aM+aT, respectively. The dynamic proliferation (H1) and movement (H2) of MSCs were measured by Cell‐IQ in groups of aM+aT, aM+aT‐Carrier, aM‐OPN+aT, aM+aT‐OPN, aM‐OPN+aT‐OPN, or M+T

OPN was found to regulate expansion, migration, and subsequent transformation of MSCs in the tissue.^28^ To validate OPN roles in the interaction between TCs and MSCs, pre‐activated TCs or MSCs (Figure 7A) were co‐cultured and OPN from either TCs as paracrine effects or MSCs as autocrine effects (Figure 7B) was neutralized using monoclonal antibody against OPN. LPS increased mRNA and protein expression of OPN in MSCs (Figure 7C) or TCs (Figure 7D) in a dose‐dependent pattern, and furthermore antibody against OPN presented MSC migration and infiltration when co‐culture with pre‐activated MSCs and TCs (Figure 7E). To define biological function and regulation either MSCs*^OPN–^* or TCs *^OPN–^*, siRNA against OPN gene inhibited OPN mRNA and protein expression in MSCs (Figure 7F1) or TCs (Figure 7F2). To further define the effective direction between MSCs and TCs, the migration capacity of MSCs was investigated in the co‐culture of MSCs with TCs under the condition of either MSCs*^OPN–^* or TCs *^OPN–^* or both. Figure 7G demonstrated that OPN contributed to the infiltration of MSCs, and OPN from activated TCs could influence MSCs even more, similar to the finding that external OPN increased survival, proliferation, migration, and differentiation of neural stem cells at a dose‐dependent pattern.^29^ OPN from MSCs or TCs played a decisive role in dynamic proliferation and movement of MSCs (Figure 7H). OPN from TCs played more important role in support of MSCs proliferation, and activated TCs could maintain dynamic proliferations of MSCs*^OPN–^*, while MSC*^OPN+^* proliferation was lower when co‐cultured with TCs*^OPN–^* (Figure 7H1). MSCs‐origin OPN is more dependent on the regulation of MSCs movement (Figure 7H2).

### 3.7 Roles of EGF‐EFGR axis in MSCs‐TCs interactions

**FIGURE 8:**
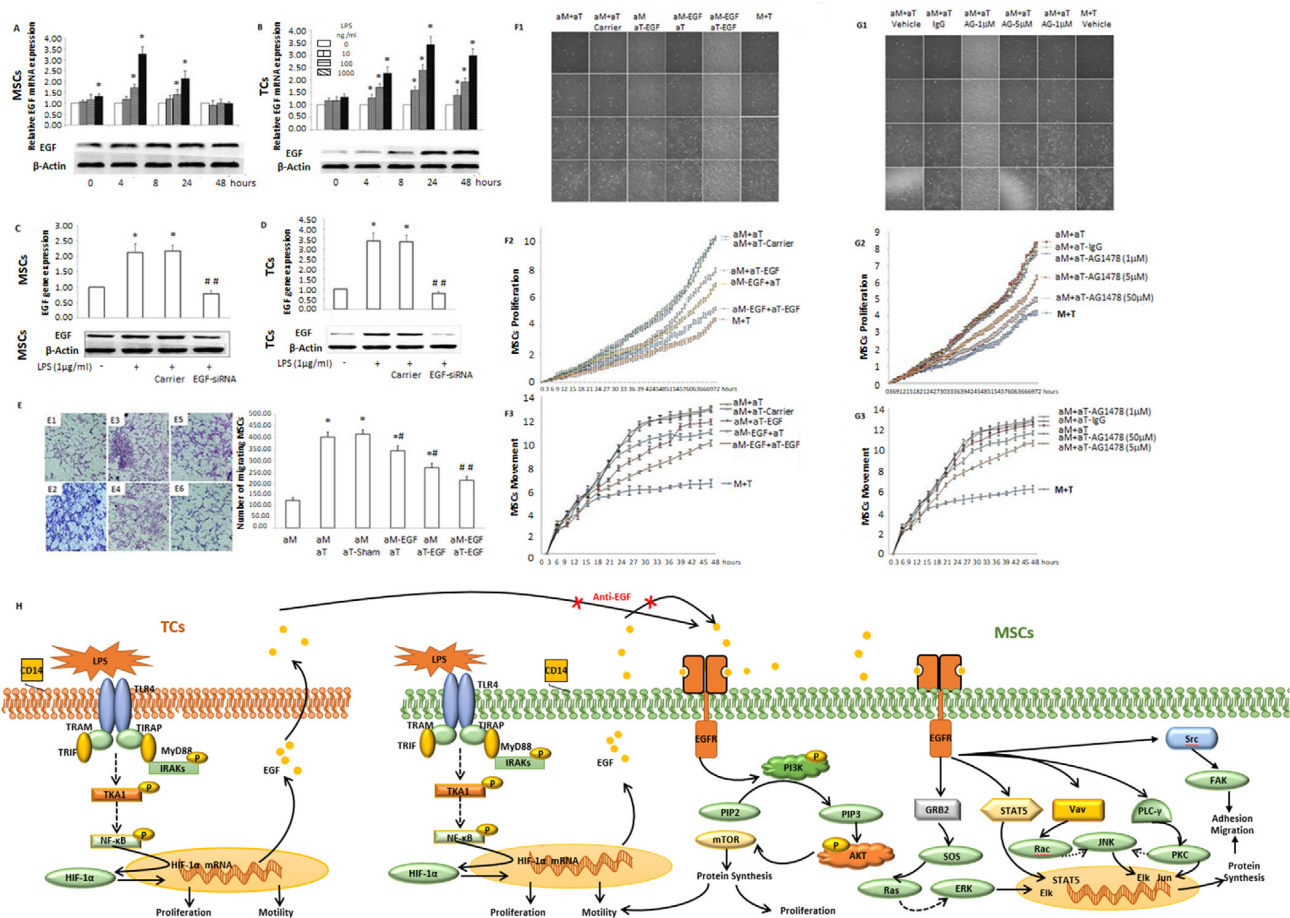
Roles of EGF‐EFGR axis in the interaction between TCs and MSCs. The expression of EGF mRNA and protein in MSCs (A) or TCs (B) were measured 0, 4, 8, 24, and 48 h after activated with LPS at different doses, while LPS‐induced mRNA and protein expressions of EGF in MSCs (C) or TCs (D) were blocked with siRNA‐EGF. * and ** stand for *P* values < .05, as compared to cells with vehicle, respectively. Effects of EGF from MSCs or TCs in the migration capacity of MSCs were evaluated (E) in capacity of the following groups: MSCs preactivated by LPS(aM), co‐cultured MSCs preactivated by LPS (aM) and TCs preactivated by LPS (aT), MSCs*^EGF‐^* preactivated by LPS (aM‐EGF), TCs*^EGF‐^* preactivated by LPS (aT‐EGF), MSCs preactivated by LPS (aM), or TCs preactivated by LPS (aT) 24 h after LPS challenge and co‐cultured MSCs preactivated by LPS (aM) and TCs preactivated by LPS without LPS challenge (aT‐Sham). * and ^#^ stand for *P* values < .05, as compared with as compared with groups aM and aM+aT, respectively. Dynamic effects of EGF originated from MSCs‐TCs contacts were investigated in the alive cell‐monitoring system (F1), including dynamic proliferation (F2) and movement (F3) of MSCs in the coculture of aM+aT, aM+aT with carrier, aM+aT‐EGF, am‐EGF+aT, aM‐EGF+aT‐EGF, or MSCs and TCs without pre‐activation (M+T). The role of EGFR in MSCs‐TCs interaction was evaluated by imaging alive cell behaviors (G1) and dynamic proliferation (G2) and movement (G3) in the co‐culture of aM+aT with vehicle, aM+aT with IgG, aM+aT with AG1478 at different doses, or M+T. Potential mechanisms of the EGF‐EFGR axis regulation in the MSCs‐TCs interaction are proposed (H)

The concentrations of growth factors (eg, EGF, VEGF) in TCs are higher than in MSCs or fibroblasts,^30^ responsible for the regulation and support of other cell growth and the formation of angiogenesis.^31^ EGF‐ or VEGF‐dominant networks in TCs became more active during the interaction with MSCs after the activation (Figure 6F and 6I). LPS could stimulate the expression of EGF mRNA and protein in MSCs with a peak at 8 h (Figure 8A) or TCs at 24 h (Figure 8B) in a dose‐dependent pattern. After selecting efficient and inhibitive effects of siRNA to block mRNA and protein expression of EGF in MSCs (Figure 8C) or TCs (Figure 8D). EGF from MSCs or TCs could regulate MSCs migration and infiltration (Figure 8E), as compared with those from TCs, and blocking EGF from both MSCs and TCs appeared synergetic inhibitory effects on MSCs migration. It was also evidenced by the finding (Figure 8F1) on dynamic proliferation (Figure 8F2) and movement (Figure 8F3) of MSCs. The EGFR is a member of human HER‐1 family and allocated on the cell surface to be recognized by EGF‐family and to over‐express in lung cancer associated with therapeutic sensitivity and prognosis in patients with lung cancer.^32^ To further investigate the role of EGFR in MSCs‐TCs interaction, we applied AG‐1478 as an EGFR kinase inhibitor to block EGFR phosphorylation and superoxide anion production (Figure 8G1) and inhibit dynamic proliferation (Figure 8G2) and movement (Figure 8G3) of MSCs during the interaction between activated both. EGF from activated MSCs through intracellular signals mainly regulated MSCs proliferation, movement, and migration, where EGF from TCs had additive effects. Molecular mechanisms of the EGF‐EFGR axis in the MSCs‐TCs interaction are summarized in Figure 8H.

## 4 DISCUSSION

Stem cell transplantation was suggested as an efficient alternative of clinical therapies for ALI /ARDS in translational medicine, although there are still some challenges to be solved.^33^ Several genes and proteins may play, at least partially, a key role in the regulation of stem cells to ameliorate experimental ALI. Transplantation of stem cells per se was found to partially prevent and treat the occurrence of ALI, while other supportive cells may benefit therapeutic effects of stem cells^34^ or MSCs efficacy might come from the paracrine activity rather than cell differentiation.^35^ Our studies provided the evidence that the key paracrine factors including OPN and EGF might be originated from both TCs and MSCs, of which TCs origins seem more supportive. In addition, platelet‐derived growth factor (PDGF) is crucial for cell survival and proliferation including MSCs and other progenitors.^36^ The co‐transplantation of MSCs and TCs had better therapeutic effects on ALI development, during which TCs could promote the migration and infiltration of MSCs into the lung. The conditioned medium containing nutrient factors also showed effectiveness in protecting lung against ALI. Intrabronchial or intravenous administration of MSCs decreased pulmonary edema, restored alveolar fluid clearance, and improved inflammation in an acute lung injury animal model.^37^ The transplantation of cardiac telocytes could reduce on severity of myocardial injury and proposed the effects of TCs on stem cells.^38^ Our data proved additional and /or synergic effects of TCs on the therapy of MSCs for experimental ALI. The interaction of MSCs and TCs plays important and direct roles in MSCs therapy for lung tissue inflammation, edema, and injury, at least partially, through mediators produced during the interaction between MSCs and TCs.

TCs could influence MSCs movement to the lung tissue and increase the appearance of MSCs in local tissues through direct interactions with MSCs or mediators released from activated TCs. Previous studies have postulated that TCs can support tissue stem cells through their extensive telopodes and 3D network.^39^ Stem cells and TCs resided in niches together where TCs may guide and nurse tissue precursors, in order to form the correct three‐dimensional tissue, embrace the precursors, and contribute to the aggregation of tissue cell clusters.^39^ The transplantation of TCs alone was found to relatively improve organ injury and inhibit about 15–25% of LPS‐induced ALI through the nutrient factors produced from TCs. Our findings suggest that factors produced from TCs or other cells interacted with TCs play the dominant role and the supportive role in therapeutic effects of co‐transplantation with MSCs and increase the distribution of MSCs into compromised lung tissues. TCs‐pre‐nutrimental and LPS‐activated MSCs appeared more efficient in the movement and therapeutic effects in ALI animals. The molecules produced from activated MSCs and TCs showed about 20–40% inhibitory effects against ALI during MSCs‐TCs transplantation.

TCs communicate with multi‐cells and support other cell functions through the connections of telopodes.^40^ CD34 positive TCs may act as progenitor cells, a nurse of stem cells, and a source of fibroblasts and myofibroblasts during tissue repair in fibrosis, granulation tissue, and tumor stroma.^6^, ^41^ We found that LPS activated TCs during the “first hit” could promote the direct effects to support and increase MSCs movement and proliferation. The activation of MSCs and LPS increased MSCs sensitivity to the message from activated TCs, leading to the optimal function of MSCs.

It is still a challenge to define how TCs interact with and benefit MSCs directly or indirectly. Previous studies demonstrated that TCs might interact with tissue stem cells through paracrine effects of extracellular vesicles released from TCs.^42^ Our data evidenced that inflammatory mediators and growth factors in TCs secretory vesicles produced during the interaction between tissue stem cells and TCs could play a modulatory role in stem cell proliferation and differentiation. TCs may act as inductors/regulators of stem cell differentiation during morphogenesis through the signal of TCs‐released molecules.^43^ We noticed that OPN‐dominant network was active in MSCs or TCs and might play supportive and nutrimental roles in the interaction, especially from activated TCs, and decisive roles in the regulation of MSCs movement, consistent with previous findings on OPN.^44^ TCs‐originated OPN was more active than MSCs, for example, endogenous and exogenous OPN increased the sensitivity of MSCs and regulated the proliferation, survival, and differentiation of MSCs, resulting in the additive treatment of co‐transplantation against ALI. In addition, we also found that the EGF‐EGFR axis signal pathway was one of mechanisms by which EGF regulated the interaction between MSCs and TCs in the therapeutic and repairing processes, although there are other signal pathways involved.^45^ LPS induced a transit or consistent overexpression of EGF gene in MSCs or TCs, respectively, and a gradual increase of EGF protein in a time‐dependent manner. MSCs and TCs act as the producer and receptor of secreted EGF and other factors (eg, KGF2, VEGF, RBP1, CX43), contributing to the cell‐cell interaction and therapeutic effects of implanted MSCs.

There are limitations of using adult bone marrow‐origined MSCs for therapy, e.g. variations of MSCs quality, stem cell senescence, and low proliferative potency. The parental pluripotent stem cells (PSC)‐derived MSCs can possess better cell quality, batch to‐batch consistency, and higher proliferative potential.^46^ GMP‐grade MSCs derived from PSCs were recently used for clinical trials to treat the refractory graft‐versus‐host‐disease.^47^ TCs‐MSCs co‐transplantation may provide another therapeutic scheme to overcome many limitations of MSCs. Another challenge is to select lung TCs‐specific biomarkers to differ from other cell types. TCs in the present study were mainly identified on basis of positive staining of CD34, c‐kit, vimentin, as well as morphology. Those markers and TCs culture conditions need to be furthermore improved to be more TCs‐specific, since those surface biomarkers (eg, Sca‐1, CD34, VCAMs, c‐kit, PDGFRα, or PDGFRβ) are also positive in pericytes or MSCs.^48^ In addition, some TCs‐like cells were observed in MSCs culture after challenge with LPS or TNF‐α.^49^ The TCs from our group have been proved for a long‐term culture as a mouse TCs line with genetic modification and used for understanding molecular mechanisms of TCs responses to drugs.^50^

In conclusion, therapeutic effects of MSCs‐TCs co‐transplantation on the severity of acute lung tissue inflammation, edema and injury critically depended upon MSCs proliferation and migration, and upon supportive roles of implanted TCs. Activated TCs promoted the proliferative and movement capacity of MSCs, and support MSCs function through the regulation of multiple factors. OPN‐dominant networks were active in both MSCs and TCs and could play supportive and nutrimental roles in cell‐cell interaction, especially from activated TCs. EGF‐EGFR axis in MSCs and TCs might play important roles in MSCs proliferation and movement during interaction, especially from activated MSCs. Our studies provide the evidence that TCs possess nutrimental and supportive roles in implanted MSCs and MSCs‐TCs co‐transplantation can be a new alternative in treatment of acute lung injury.

## AUTHOR CONTRIBUTIONS

DZH, DLS, LS, and XRS designed the study and completed the experimental process, literature search, and generation of figures. DZH, DLS, and XDW wrote and edited the manuscript. DLS, LS, XRS, YHZ, and XDW completed generation of figures. LS, XRS, and YMZ performed bioinformatics and statistical analysis. All authors reviewed the manuscript. All authors read and approved the final manuscript.

## CONFLICT OF INTEREST

The authors declare that they have no conflict of interest.

## Supporting information



Supporting Figure S1

Supporting Figure S2

Supporting Table S1

Supporting Table S2

Supporting Information
