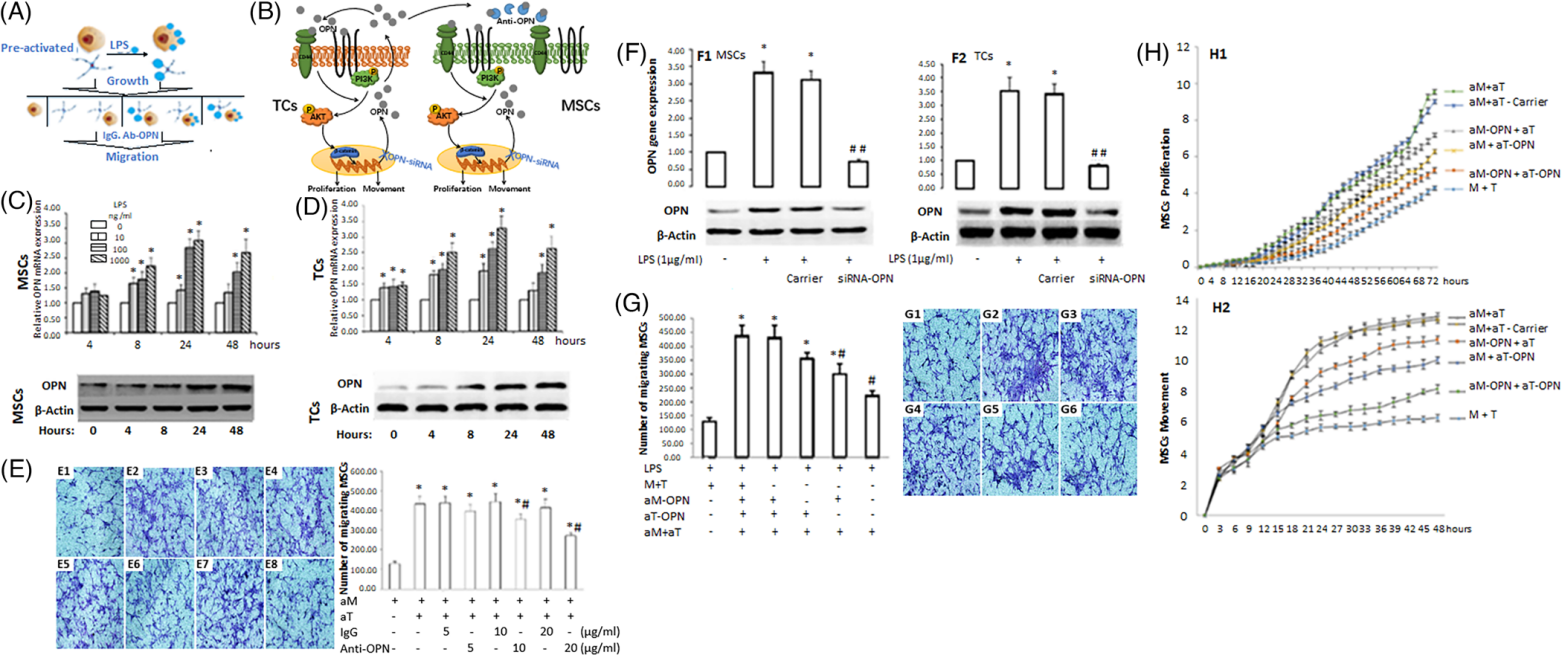# Correction to: Mechanisms of interactions between lung‐origin telocytes and mesenchymal stem cells to treat experimental acute lung injury

**DOI:** 10.1002/ctm2.1342

**Published:** 2023-07-28

Ding Zhang,^#^ Dongli Song,^#^ Lin Shi,^#^ Xiaoru Sun,^#^ Yonghua Zheng,^#^ Yiming Zeng and Xiangdong Wang

Following publication of the original article,^1^ the authors identified the errors in Figure 7E and 7G, where the images of OPN effects on MSCs migration were incorrect.

The updated Figure 7 is provided.

The authors apologize for this error.

**FIGURE 7:**