# Supplementary figures and images for: Mechanisms of interactions between lung‐origin telocytes and mesenchymal stem cells to treat experimental acute lung injury
Source: Clin Transl Med. 2020 Dec 8;10(8):e231. doi: 10.1002/ctm2.231 (PMC7724099; doi:10.1002/ctm2.231)

## Slide 1
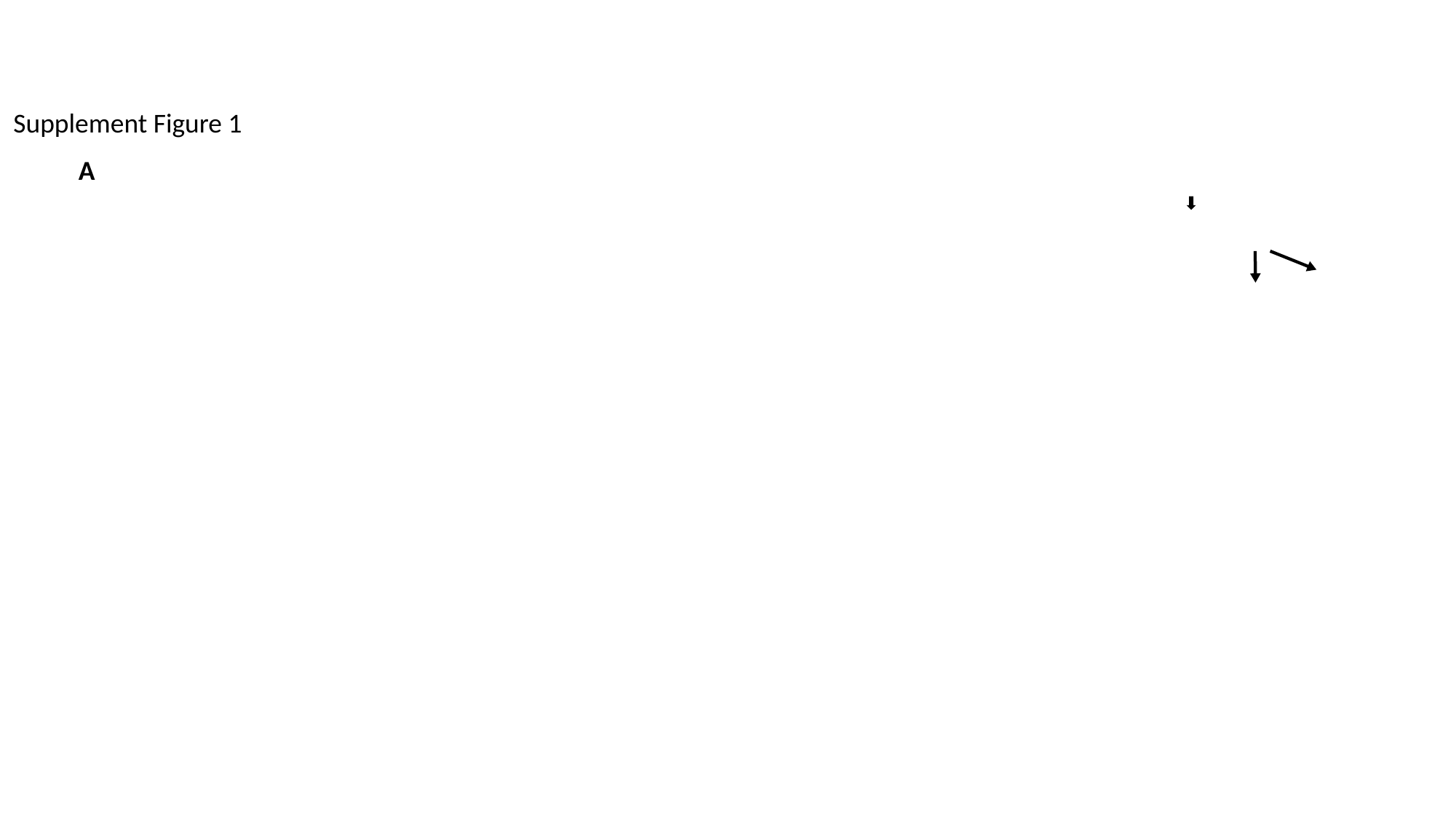

Supplement Figure 1

Supplement: Supplementary file 1 — Supporting Figure S1 [file CTM2-10-e231-s001.pptx]

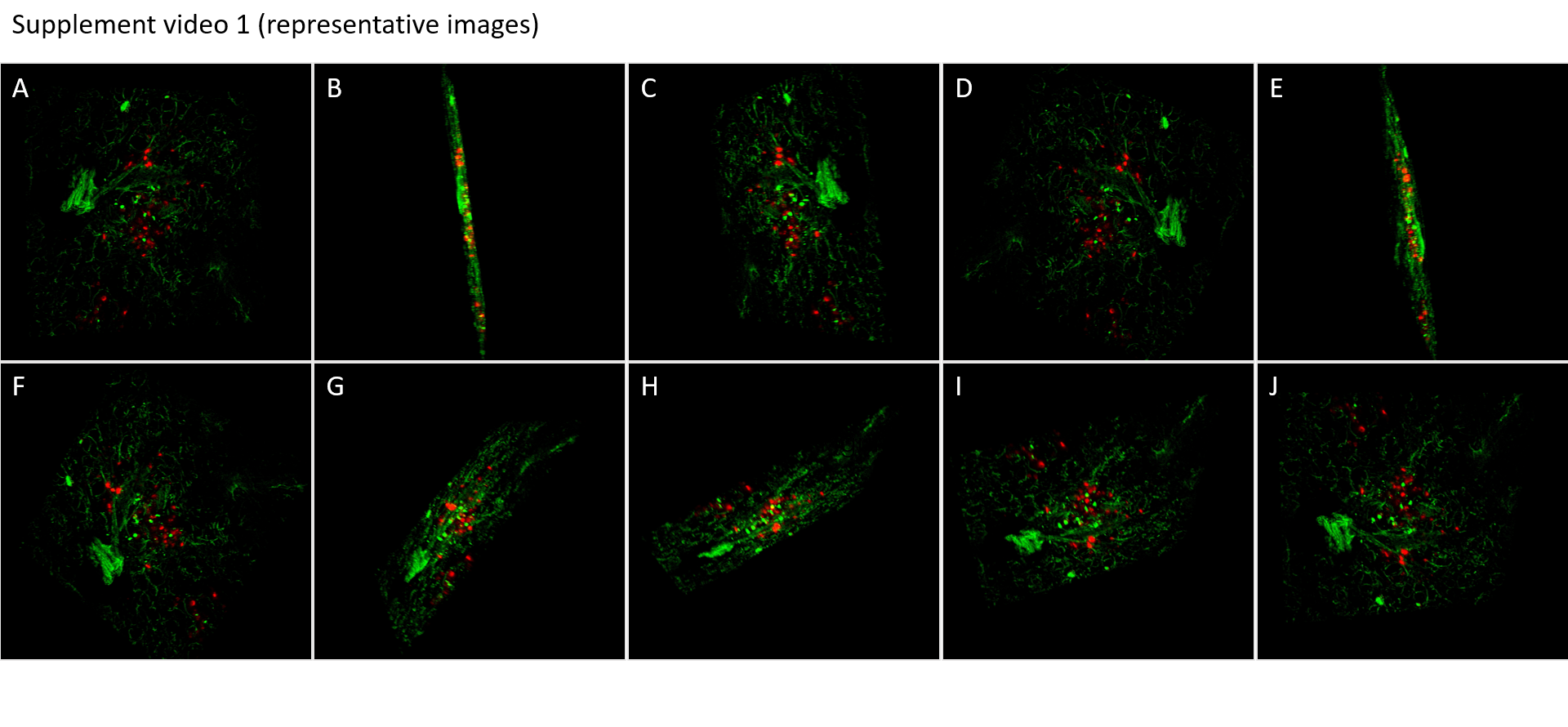

Supplement: Supplementary file 5 — Supporting Information [file CTM2-10-e231-s005.tif]
