# Supplementary material for: Mechanisms of interactions between lung‐origin telocytes and mesenchymal stem cells to treat experimental acute lung injury
Source: Clin Transl Med. 2020 Dec 8;10(8):e231. doi: 10.1002/ctm2.231 (PMC7724099; doi:10.1002/ctm2.231)
Supplement: Supplementary file 2 — Supporting Figure S2 [file CTM2-10-e231-s002.pdf]

Supplement Figure 2

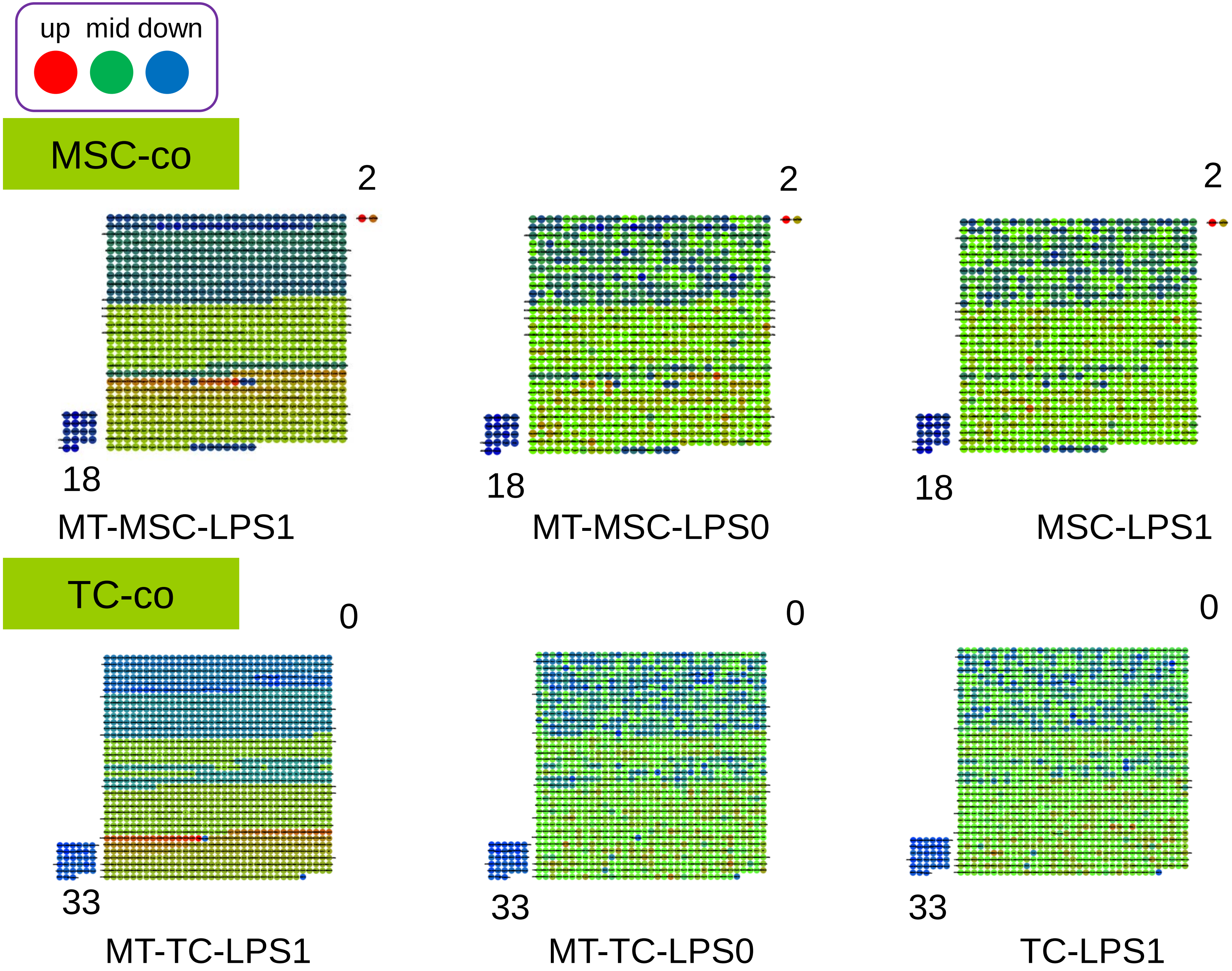

OPN-SPP1

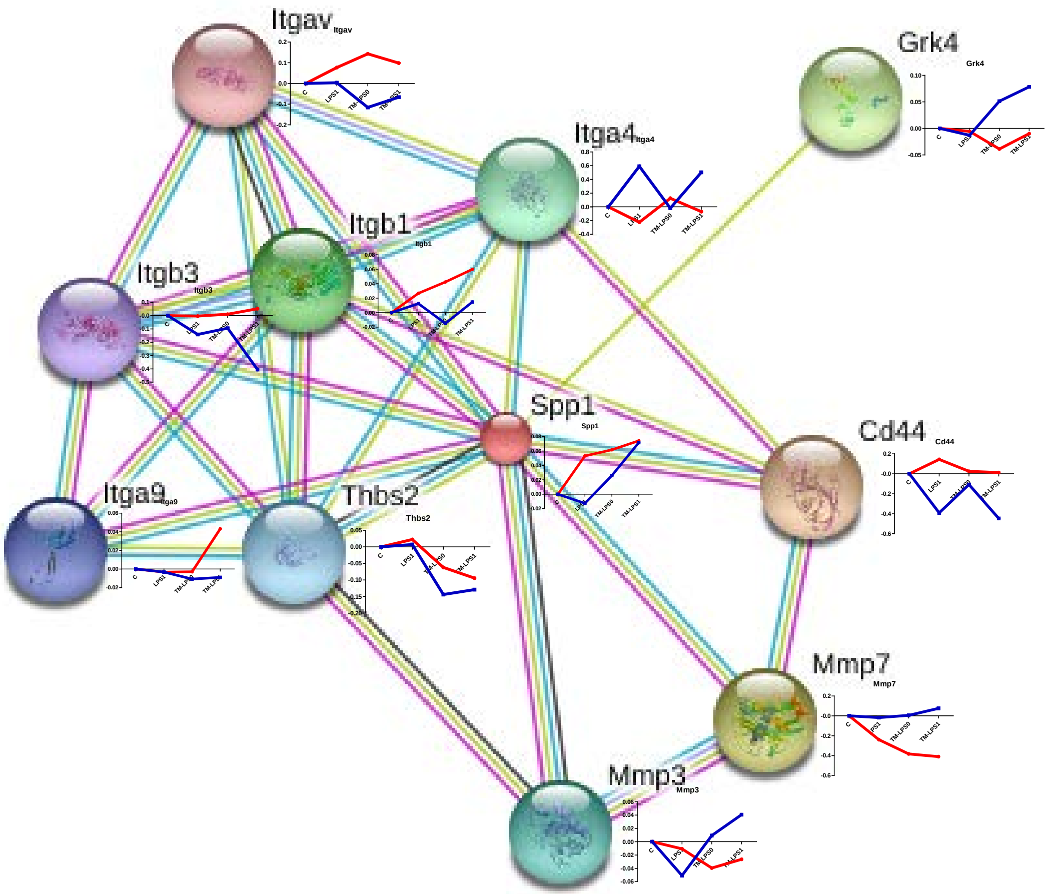

Itgav
Grk4
Itga4
Itgb1
Itgb3
Spp1
Cd44
Itga9
Thbs2
Mmp7
Mmp3

OPN-SPP1 family

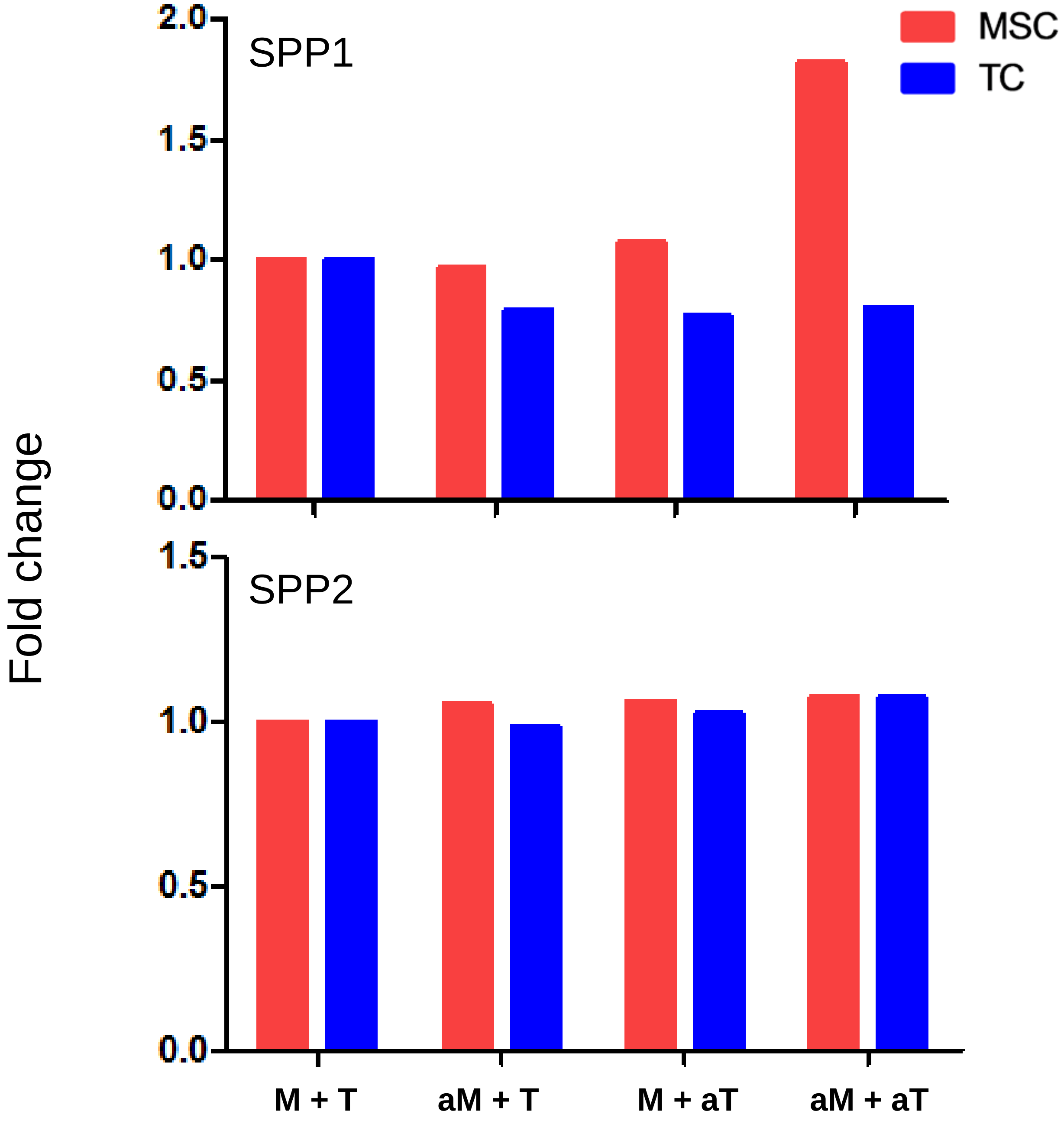

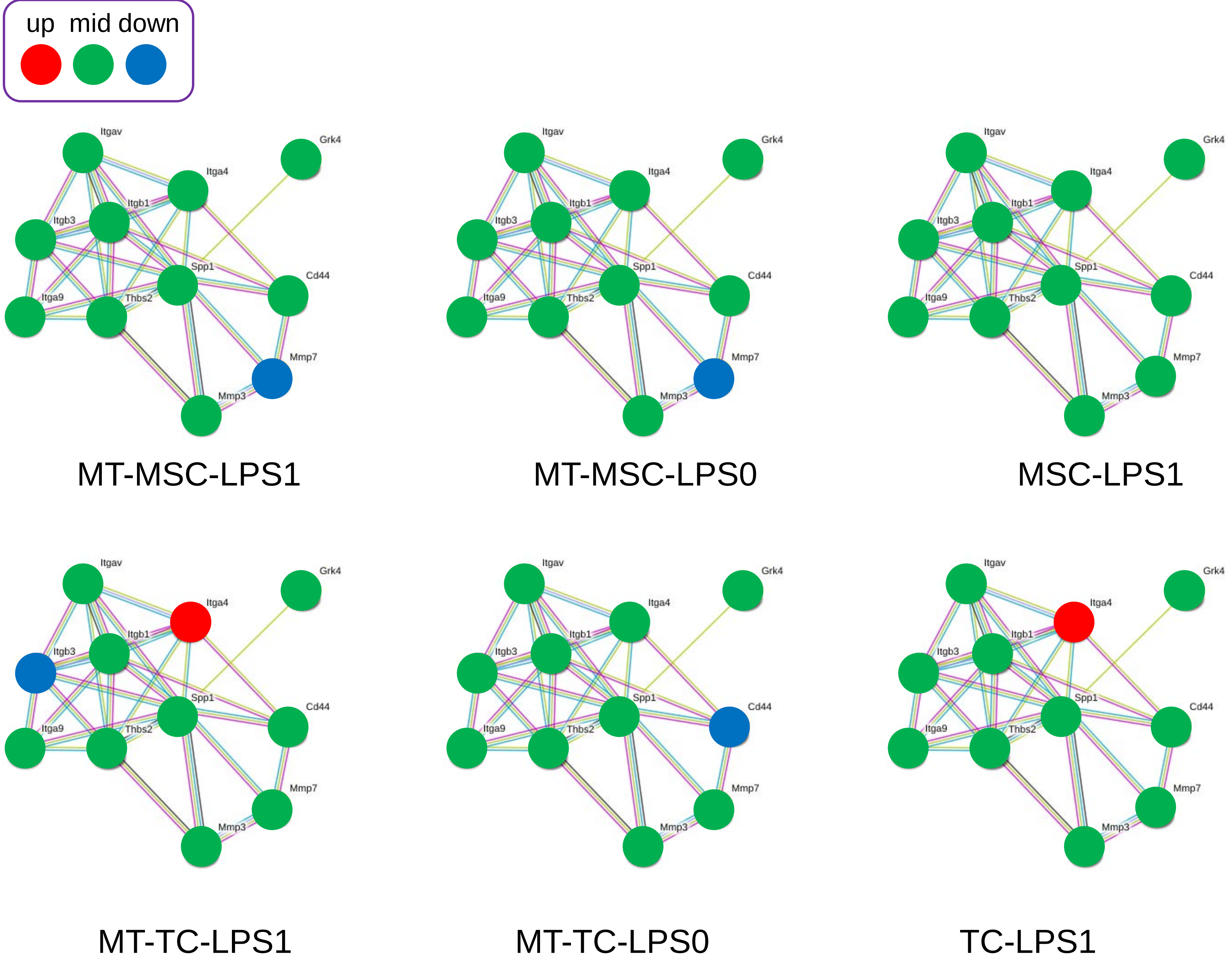
up mid down
Itgav
Grk4
Itga4
Itgb1
Itgb3
Spp1
Cd44
Itga9
Thbs2
Mmp7
Mmp3
MT-MSC-LPS1
Itgav
Grk4
Itga4
Itgb1
Itgb3
Spp1
Cd44
Itga9
Thbs2
Mmp7
Mmp3
MT-MSC-LPS0
Itgav
Grk4
Itga4
Itgb1
Itgb3
Spp1
Cd44
Itga9
Thbs2
Mmp7
Mmp3
MSC-LPS1
Itgav
Grk4
Itga4
Itgb1
Itgb3
Spp1
Cd44
Itga9
Thbs2
Mmp7
Mmp3
MT-TC-LPS1
Itgav
Grk4
Itga4
Itgb1
Itgb3
Spp1
Cd44
Itga9
Thbs2
Mmp7
Mmp3
MT-TC-LPS0
Itgav
Grk4
Itga4
Itgb1
Itgb3
Spp1
Cd44
Itga9
Thbs2
Mmp7
Mmp3
TC-LPS1

KGF2-FGF10

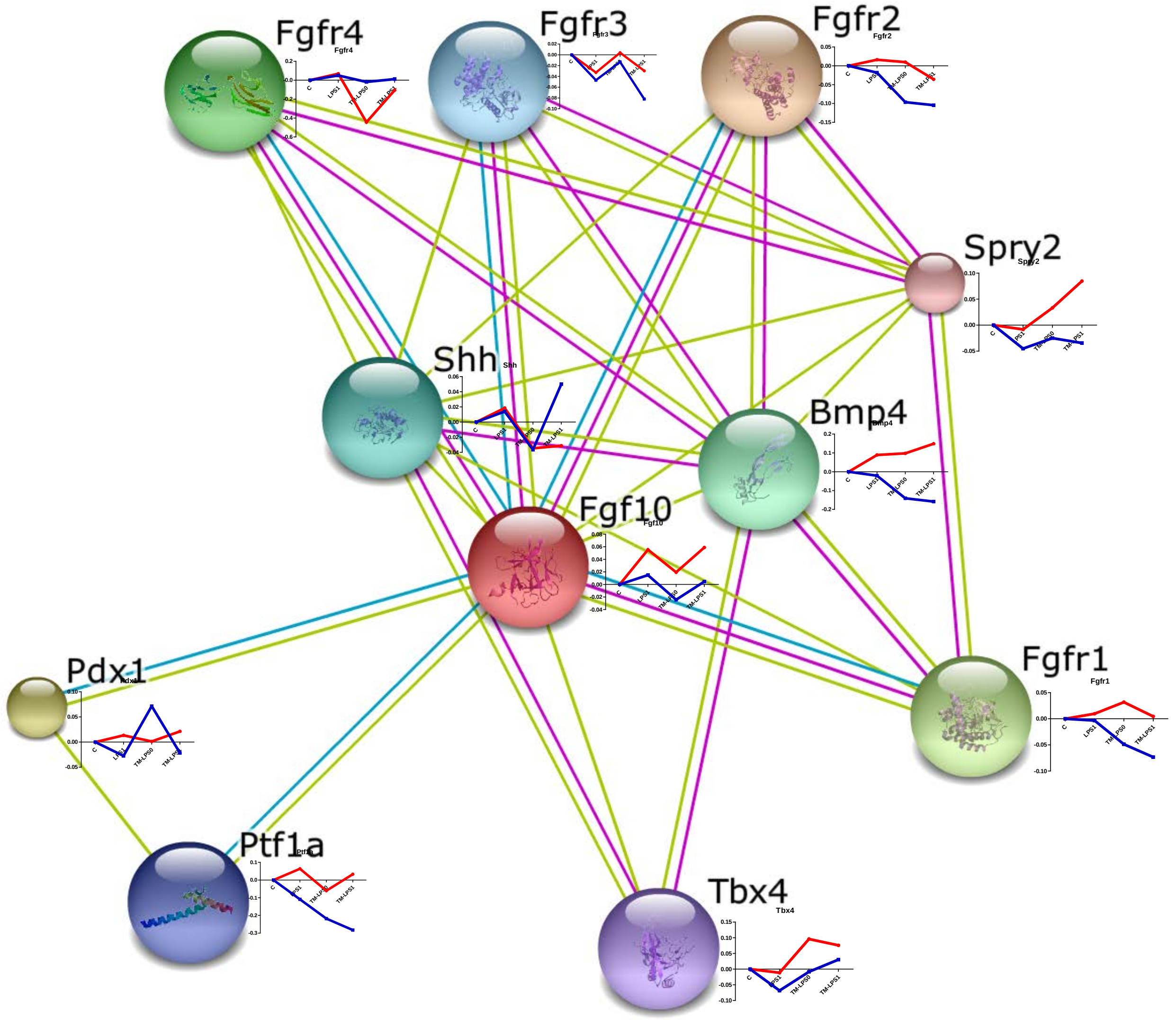
Fgfr4
Fgfr3
Fgfr2
Spry2
Shh
Bmp4
Fgf10
Pdx1
Fgfr1
Ptf1a
Tbx4

KGF2-FGF10 family

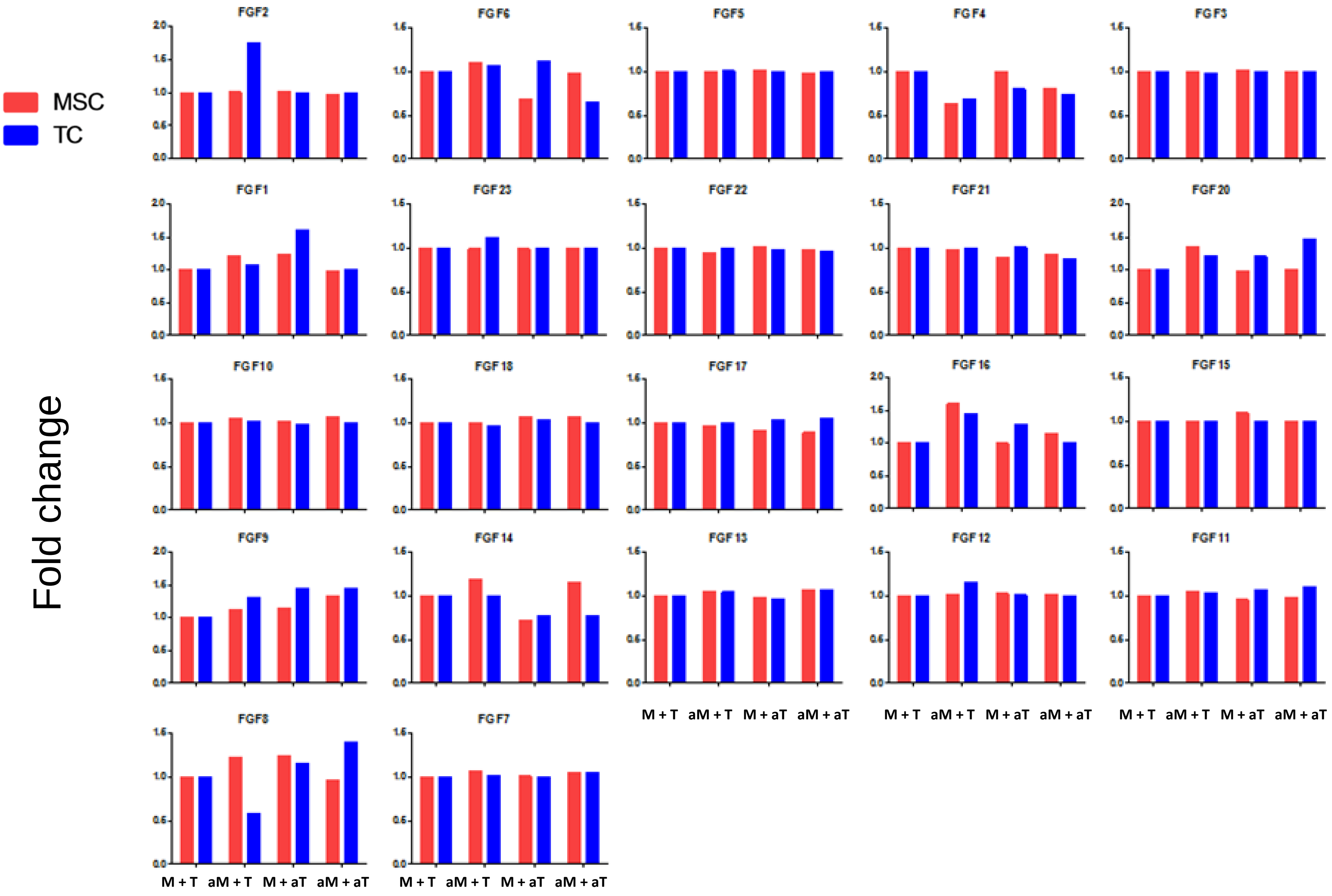

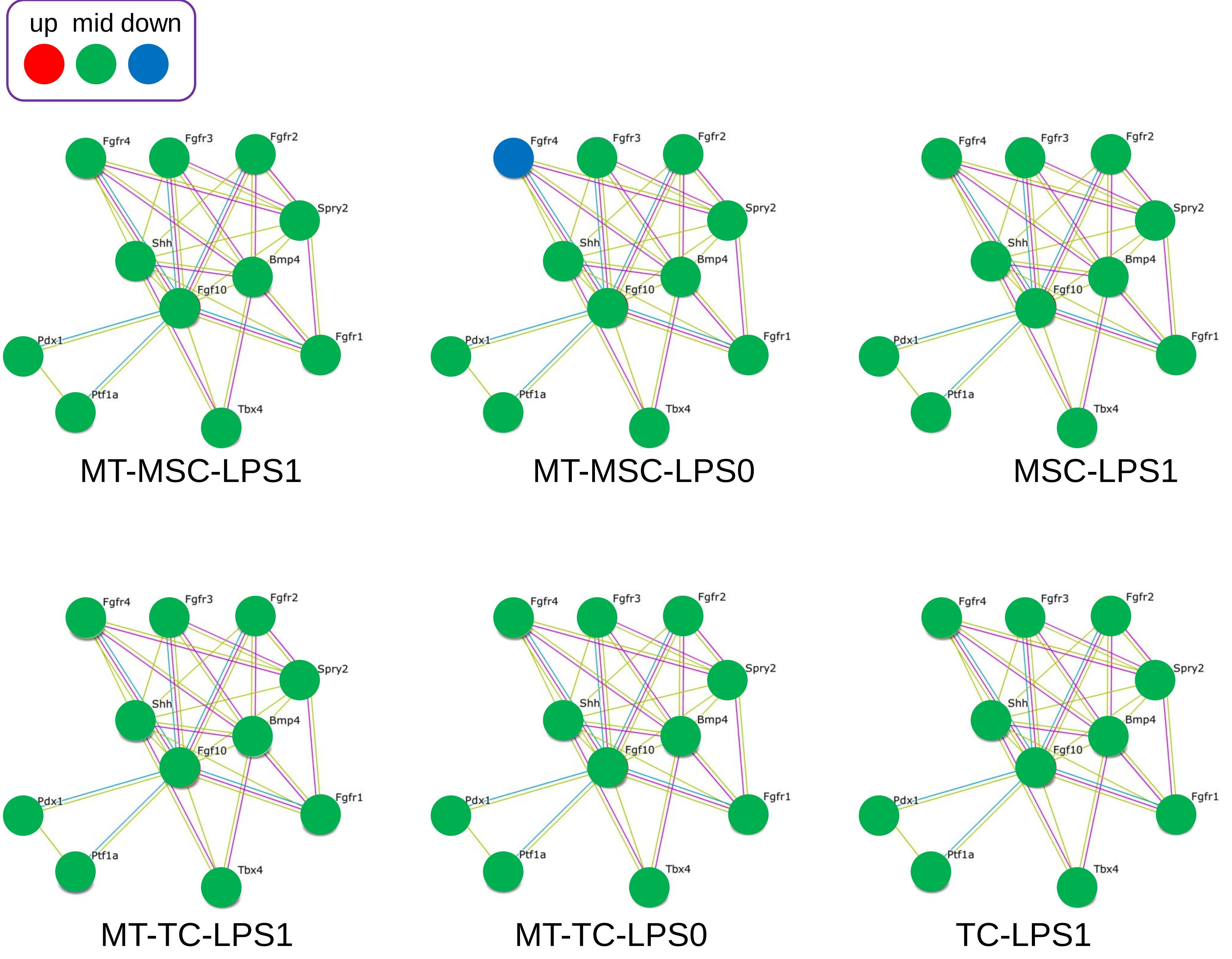
up mid down
Fgfr4
Fgfr3
Fgfr2
Spry2
Shh
Bmp4
Fgf10
Pdx1
Fgfr1
Ptf1a
Tbx4
MT-MSC-LPS1
MT-MSC-LPS0
MSC-LPS1
MT-TC-LPS1
MT-TC-LPS0
TC-LPS1

CX43-GJA1

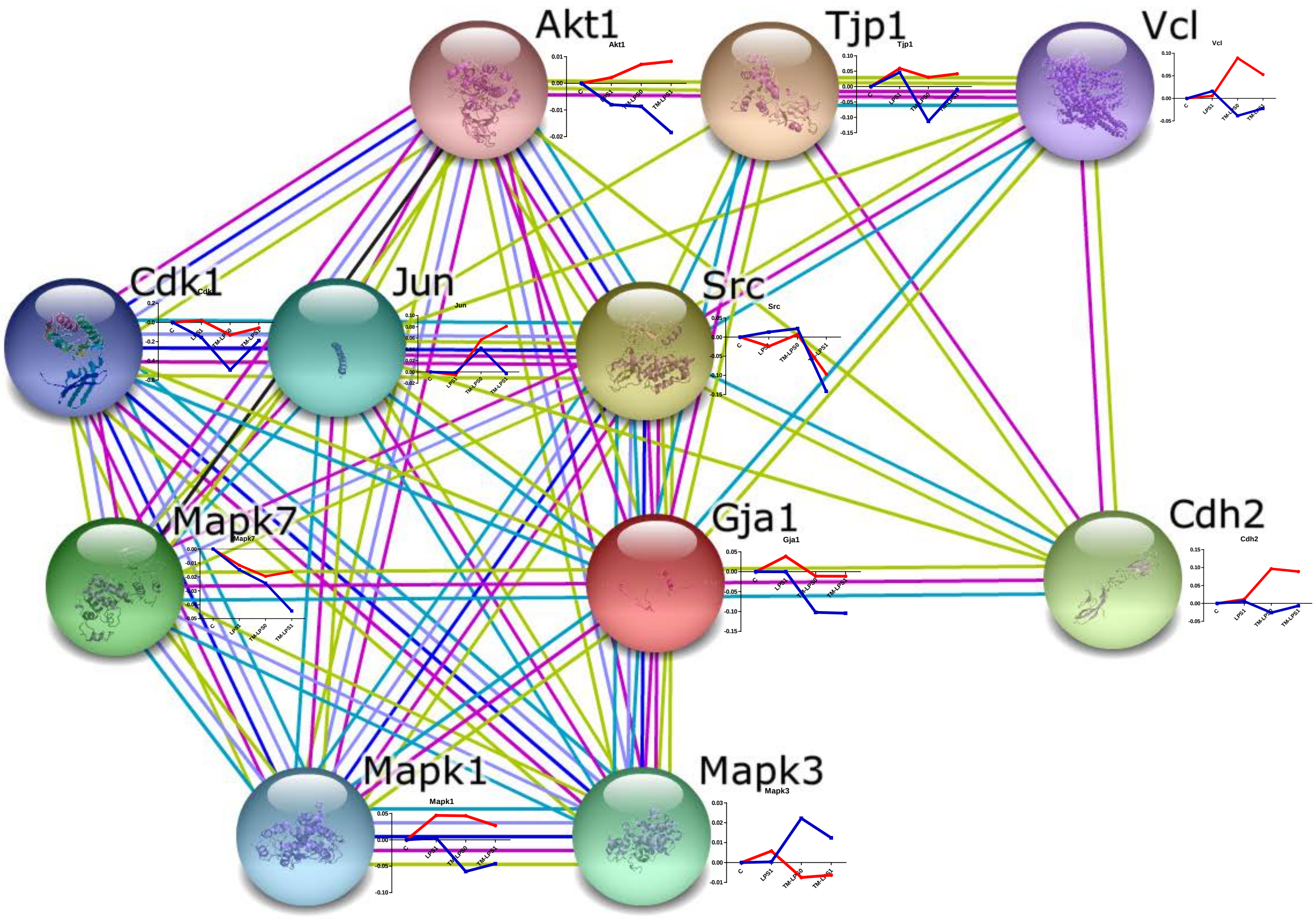

CX43-GJA1 family

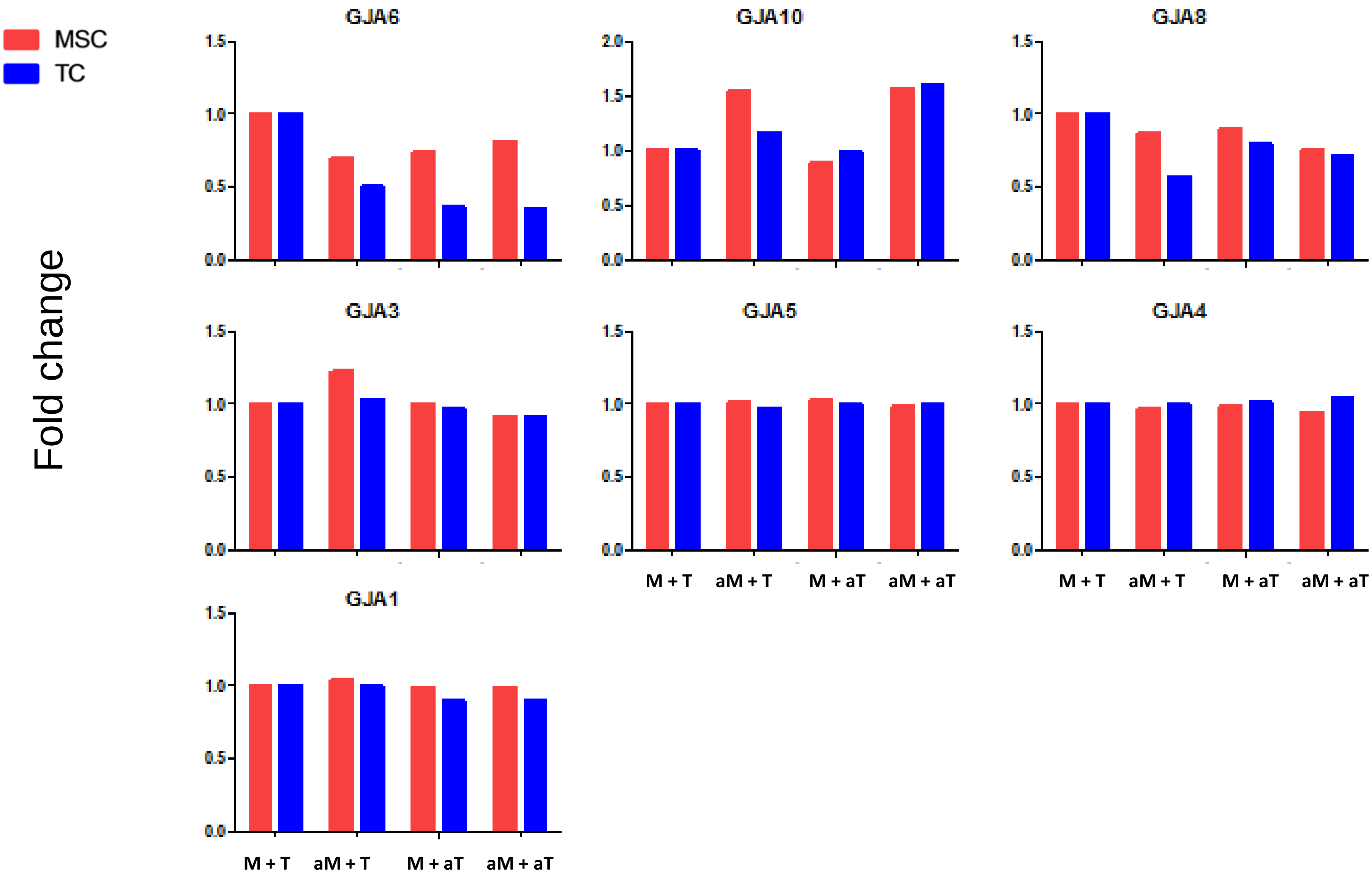

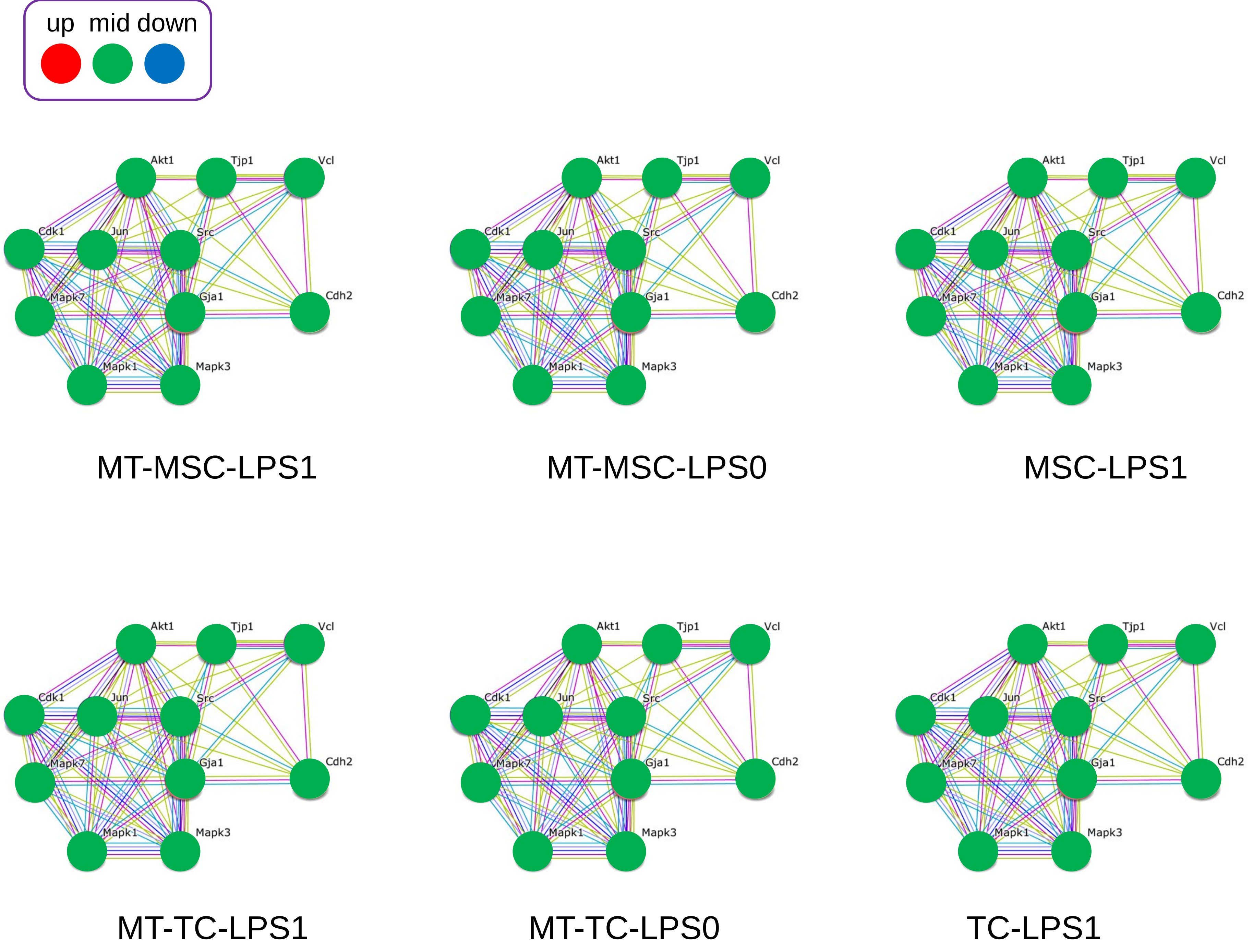
up mid down
Akt1
Tjp1
Vcl
Cdk1
Jun
Src
Mapk7
Gja1
Cdh2
Mapk1
Mapk3
MT-MSC-LPS1
MT-MSC-LPS0
MSC-LPS1
MT-TC-LPS1
MT-TC-LPS0
TC-LPS1

EGF

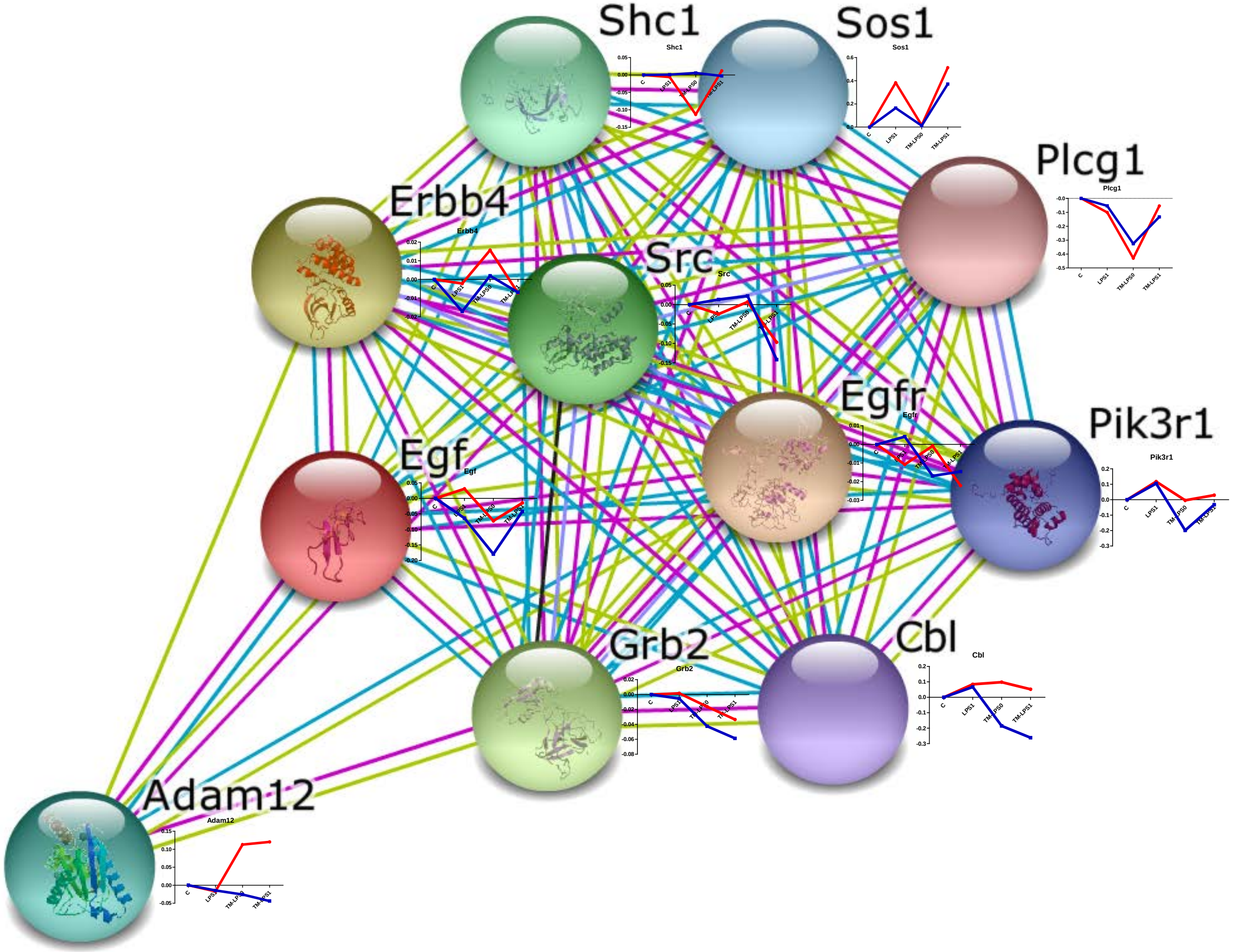

EGF family

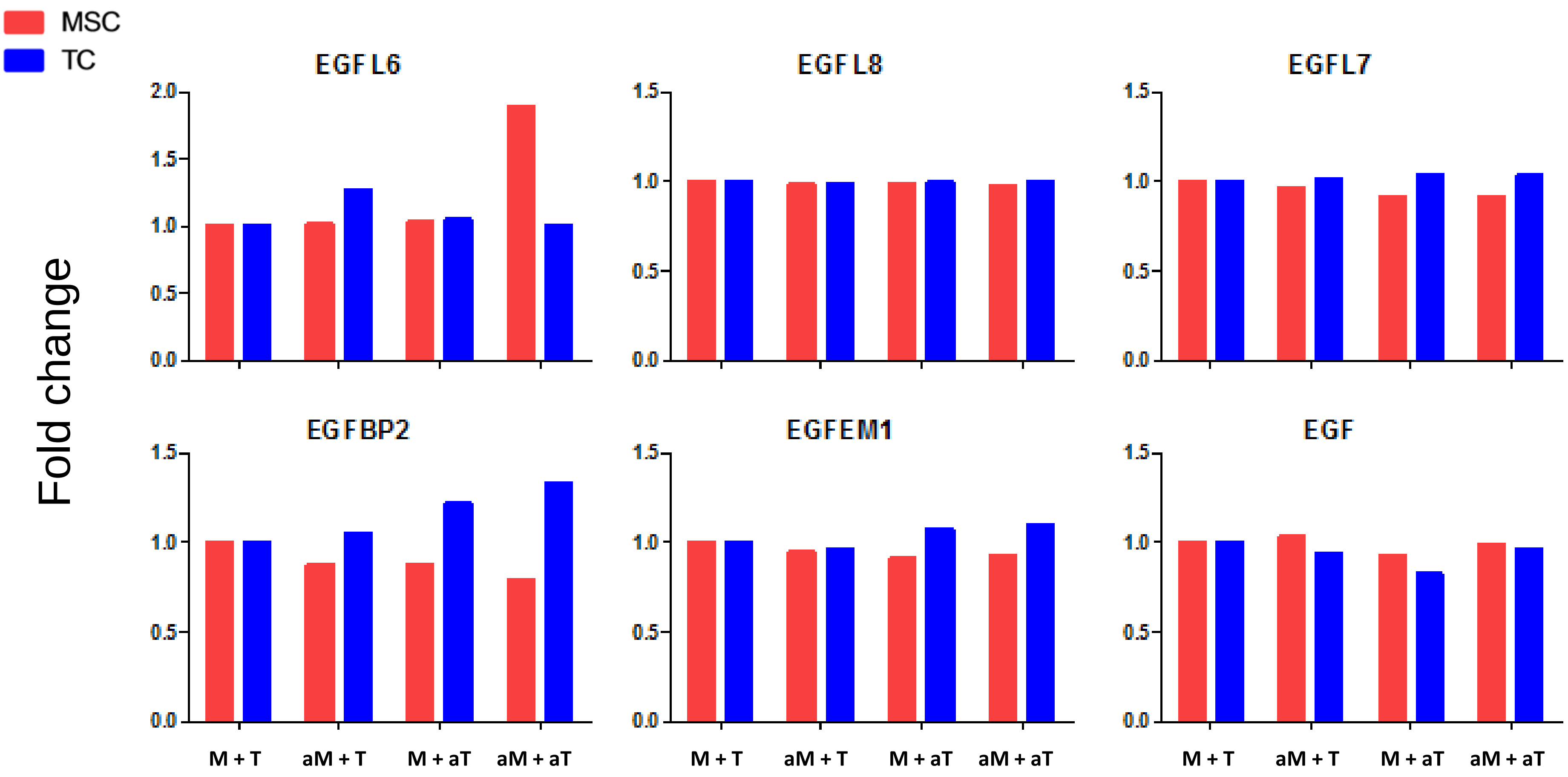
MSC
TC
Fold change
EGFL6
EGFL8
EGFL7
EGFBP2
EGFEM1
EGF
2.0
1.5
1.0
0.5
0.0
M + T
aM + T
M + aT
aM + aT

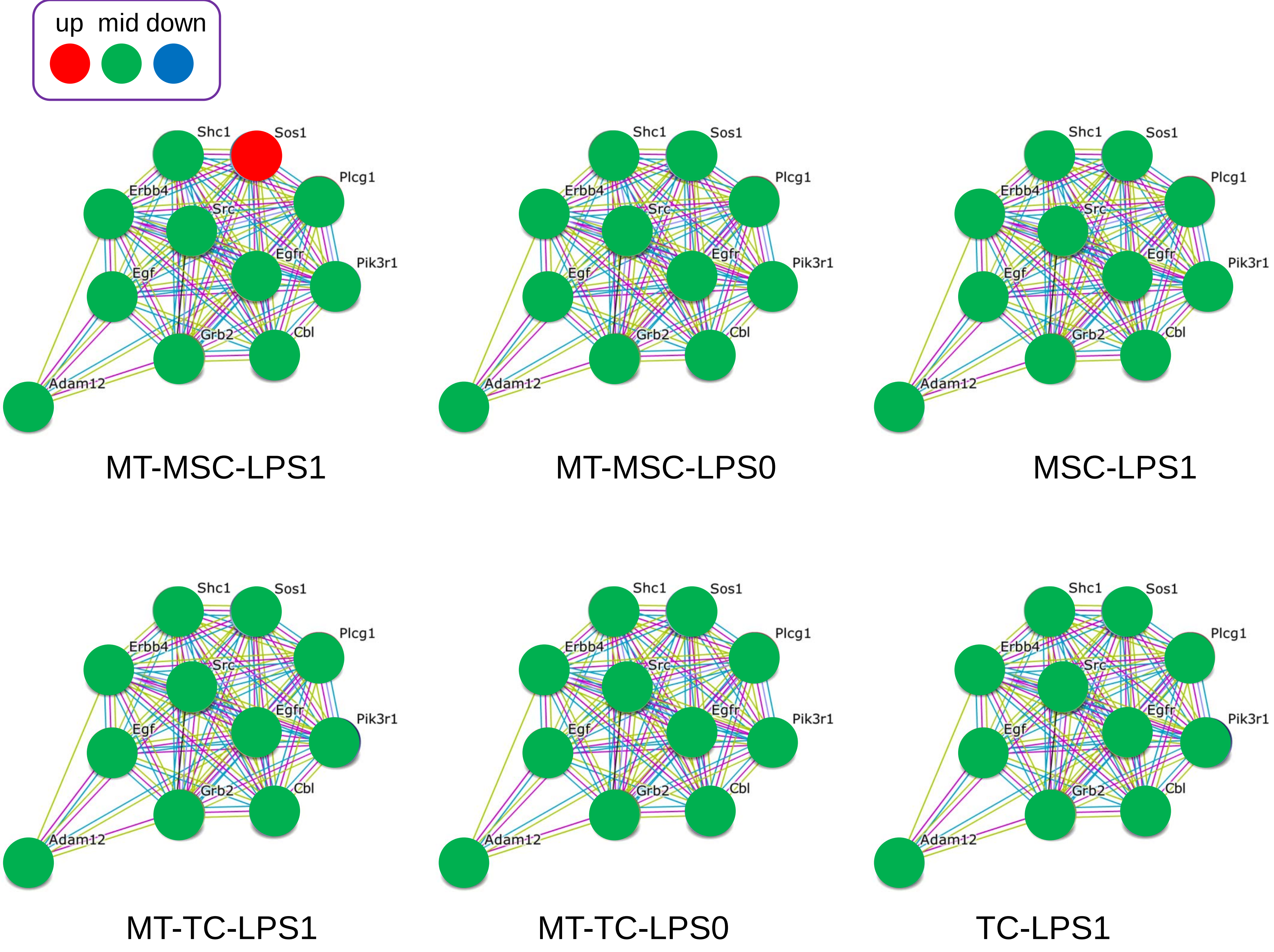
up mid down
Shc1
Sos1
Plcg1
Erbb4
Src
Egfr
Pik3r1
Egf
Grb2
Cbl
Adam12
MT-MSC-LPS1
Shc1
Sos1
Plcg1
Erbb4
Src
Egfr
Pik3r1
Egf
Grb2
Cbl
Adam12
MT-MSC-LPS0
Shc1
Sos1
Plcg1
Erbb4
Src
Egfr
Pik3r1
Egf
Grb2
Cbl
Adam12
MSC-LPS1
Shc1
Sos1
Plcg1
Erbb4
Src
Egfr
Pik3r1
Egf
Grb2
Cbl
Adam12
MT-TC-LPS1
Shc1
Sos1
Plcg1
Erbb4
Src
Egfr
Pik3r1
Egf
Grb2
Cbl
Adam12
MT-TC-LPS0
Shc1
Sos1
Plcg1
Erbb4
Src
Egfr
Pik3r1
Egf
Grb2
Cbl
Adam12
TC-LPS1

RBP1

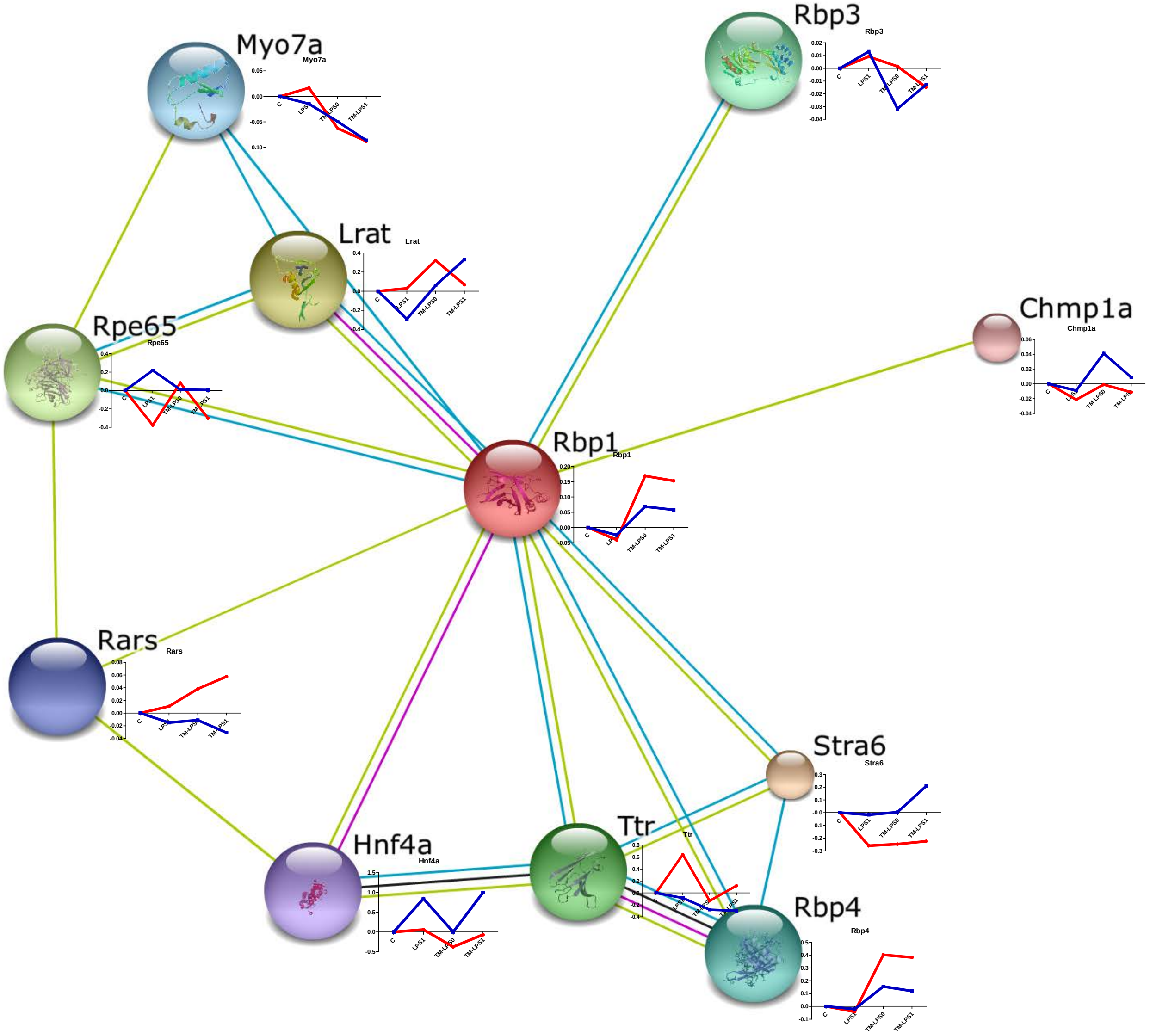

RBP family

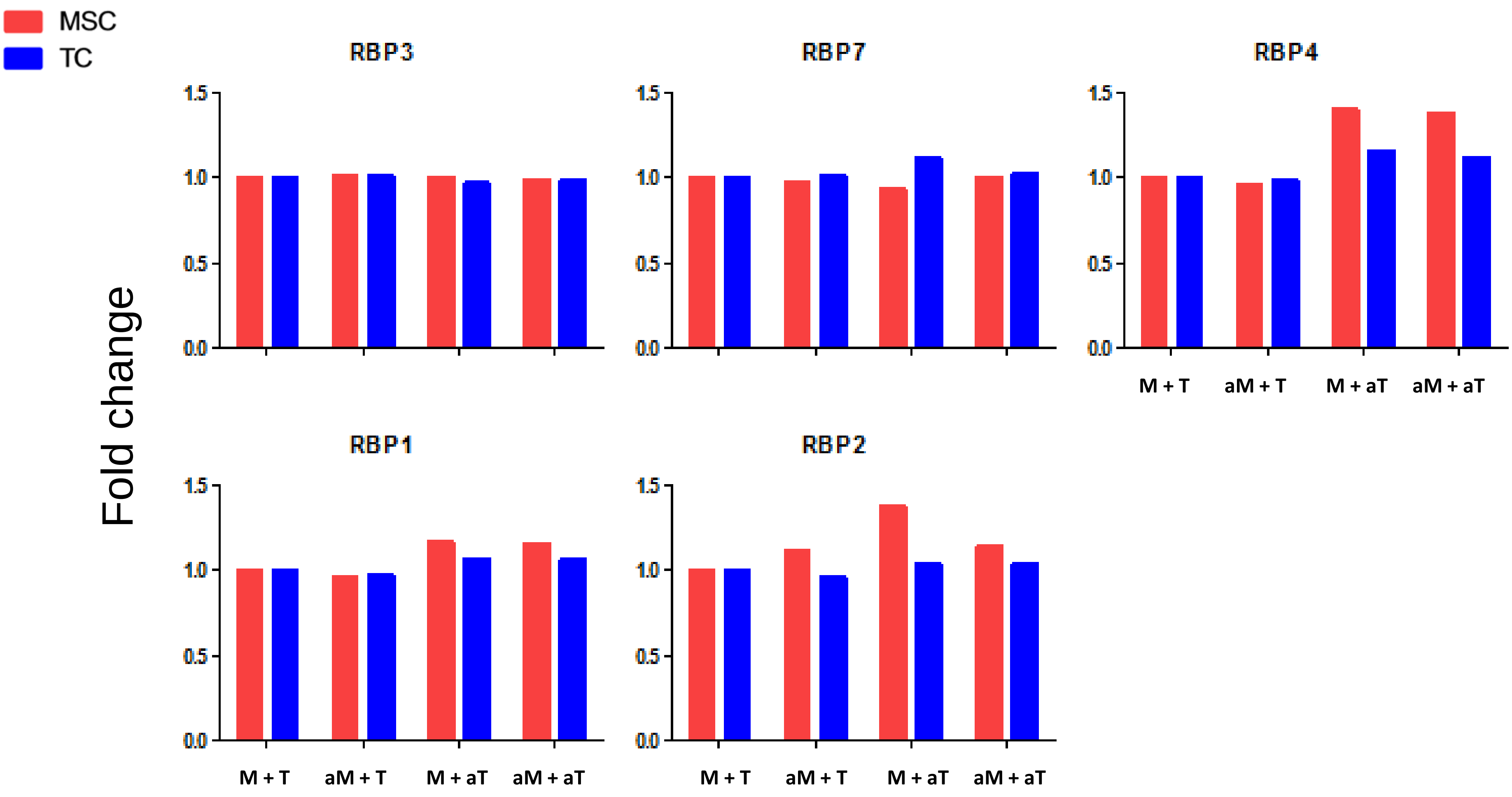

MSC
TC
Fold change
RBP3
RBP7
RBP4
RBP1
RBP2
0.0
0.5
1.0
1.5
M + T
aM + T
M + aT
aM + aT

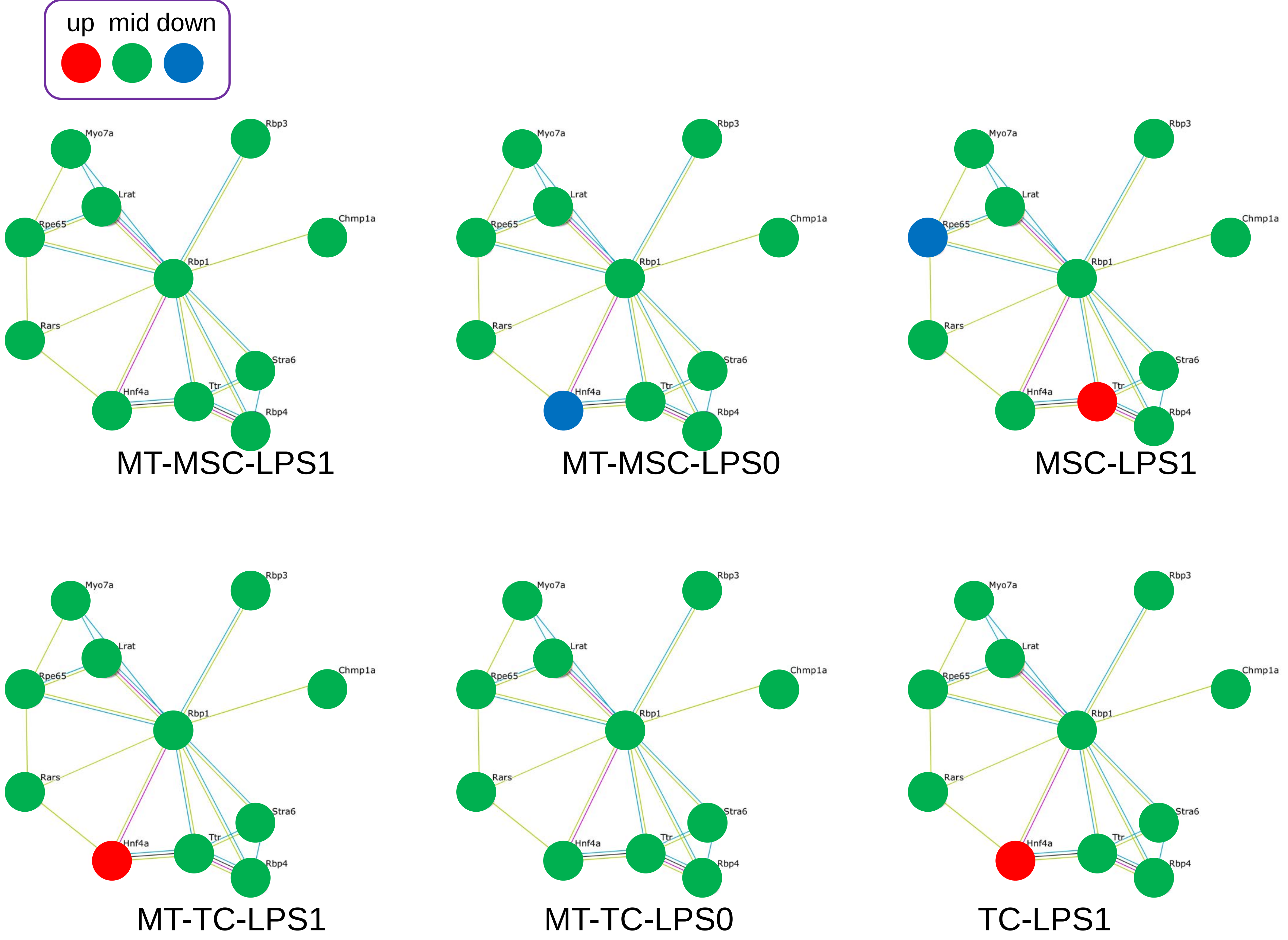
up mid down
Myo7a
Rbp3
Lrat
Chmp1a
Rpe65
Rbp1
Rars
Stra6
Hnf4a
Ttr
Rbp4
MT-MSC-LPS1
MT-MSC-LPS0
MSC-LPS1
MT-TC-LPS1
MT-TC-LPS0
TC-LPS1

VEGF

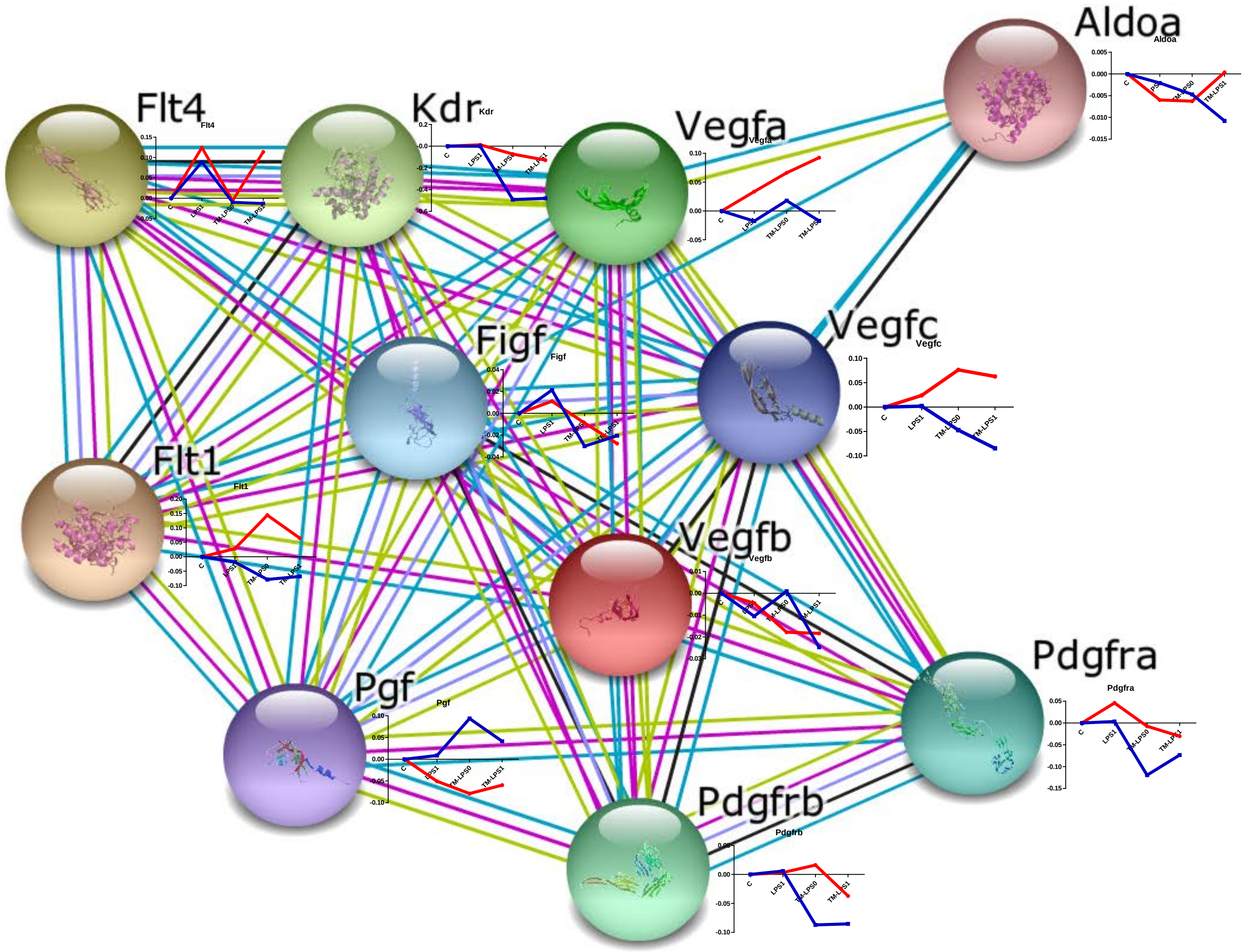
Aldoa
Flt4
Kdr
Vegfa
Vegfc
Figf
Flt1
Vegfb
Pdgfra
Pgf
Pdgfrb

VEGF family

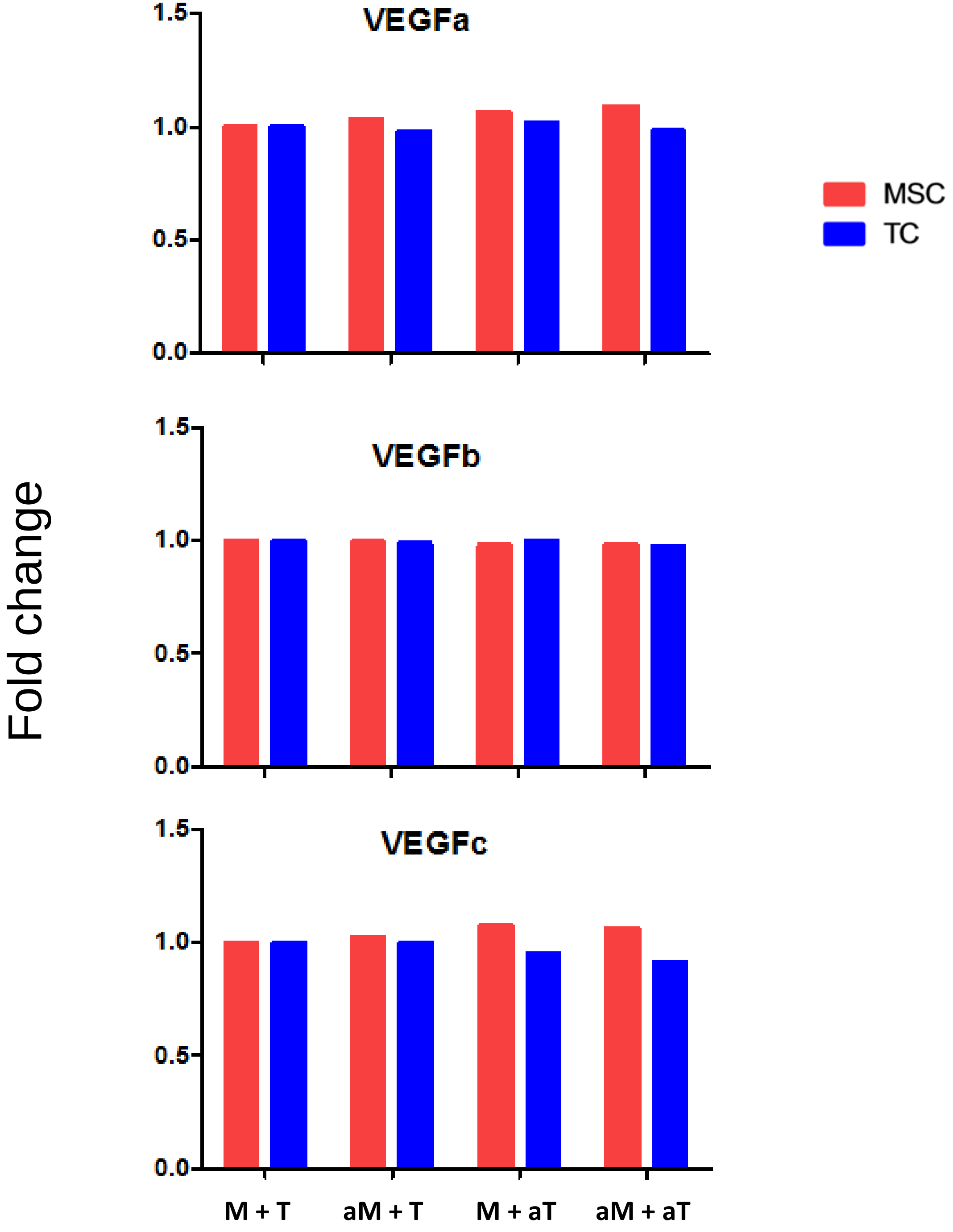

VEGFa
VEGFb
VEGFc
Fold change
1.5
1.0
0.5
0.0
MSC
TC
M + T
aM + T
M + aT
aM + aT

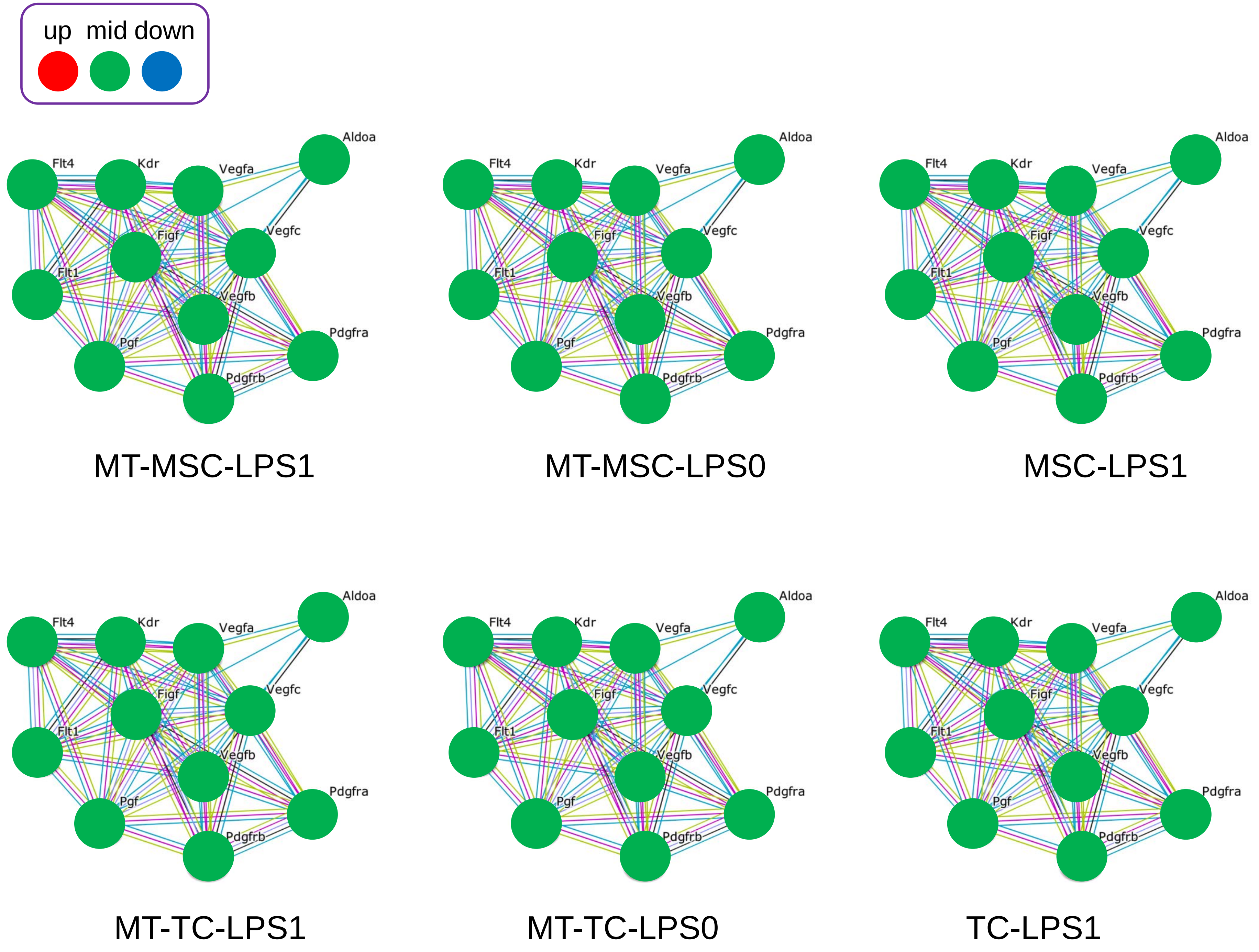
up mid down
Flt4
Kdr
Vegfa
Aldoa
Figf
Vegfc
Flt1
Vegfb
Pgf
Pdgfra
Pdgfrb
MT-MSC-LPS1
Flt4
Kdr
Vegfa
Aldoa
Figf
Vegfc
Flt1
Vegfb
Pgf
Pdgfra
Pdgfrb
MT-MSC-LPS0
Flt4
Kdr
Vegfa
Aldoa
Figf
Vegfc
Flt1
Vegfb
Pgf
Pdgfra
Pdgfrb
MSC-LPS1
Flt4
Kdr
Vegfa
Aldoa
Figf
Vegfc
Flt1
Vegfb
Pgf
Pdgfra
Pdgfrb
MT-TC-LPS1
Flt4
Kdr
Vegfa
Aldoa
Figf
Vegfc
Flt1
Vegfb
Pgf
Pdgfra
Pdgfrb
MT-TC-LPS0
Flt4
Kdr
Vegfa
Aldoa
Figf
Vegfc
Flt1
Vegfb
Pgf
Pdgfra
Pdgfrb
TC-LPS1
