# Supplementary material for: Mechanisms of interactions between lung‐origin telocytes and mesenchymal stem cells to treat experimental acute lung injury
Source: Clin Transl Med. 2020 Dec 8;10(8):e231. doi: 10.1002/ctm2.231 (PMC7724099; doi:10.1002/ctm2.231)
Supplement: Supplementary file 3 — Supporting Table S1 [file CTM2-10-e231-s003.docx]

Supplement table 1：Alterations of gene expression profiles of MSCs caused by the interaction of TCs and MSCs.

(A) Genes gene expression profiles in MSCs stimulated with 1μg/ml LPS compared with MSCs.

| GeneSymbol | MSCs | MSCs stimulated with 1μg/ml LPS | Fold Change |
| --- | --- | --- | --- |
| 4930435E12Rik | 6.8268404 | 2.0781744 | -0.69558767 |
| Gm10487 | 6.600509 | 2.1493344 | -0.67436839 |
| Hrasls | 6.436921 | 2.1206045 | -0.67055608 |
| 4930451I11Rik | 6.049219 | 2.110451 | -0.65112009 |
| Ermap | 5.751102 | 2.1026568 | -0.63439063 |
| 2610018G03Rik | 5.5079255 | 2.077877 | -0.6227478 |
| Vmn2r37 | 5.5317235 | 2.0938113 | -0.62149025 |
| Sycp3 | 5.473989 | 2.1174865 | -0.61317304 |
| Tlx3 | 5.407043 | 2.1085808 | -0.6100307 |
| 1700013N18Rik | 5.402881 | 2.1158738 | -0.60838046 |
| Igh-VJ558 | 5.359541 | 2.1584196 | -0.59727529 |
| Rsph10b2 | 5.158323 | 2.106323 | -0.59166516 |
| Mgam | 5.173423 | 2.127077 | -0.58884534 |
| Krt26 | 5.2349377 | 2.1846752 | -0.58267408 |
| Hrk | 5.1569633 | 2.2047193 | -0.57247722 |
| Olfr366 | 5.0788374 | 2.1948872 | -0.56783669 |
| Olfr1155 | 4.860658 | 2.1448534 | -0.55873188 |
| Rnase11 | 4.753851 | 2.1410358 | -0.54962076 |
| 2410004P03Rik | 4.760647 | 2.1675544 | -0.54469332 |
| 1700110I01Rik | 4.668014 | 2.1566918 | -0.53798515 |
| Olfr243 | 4.584425 | 2.133131 | -0.53470043 |
| Patl2 | 4.512514 | 2.1014848 | -0.53429844 |
| Gfi1 | 4.3826065 | 2.0918393 | -0.52269516 |
| Ncam2 | 4.4337535 | 2.1218765 | -0.5214266 |
| Zfp874a | 4.367638 | 2.101791 | -0.51878086 |
| B4galnt3 | 4.349327 | 2.1007776 | -0.51698789 |
| Actrt2 | 4.4131794 | 2.1579642 | -0.51101825 |
| Cd164l2 | 4.4725447 | 2.1883051 | -0.51072482 |
| Gm5941 | 4.388052 | 2.1862967 | -0.50176144 |
| Klrc2 | 4.318441 | 2.1569517 | -0.50052538 |
| Slc10a2 | 4.0603304 | 2.0689092 | -0.49045792 |
| Resp18 | 4.07533 | 2.0800588 | -0.48959746 |
| Ccdc147 | 4.352293 | 2.2246048 | -0.48886603 |
| Npffr1 | 4.2261415 | 2.1666262 | -0.48732758 |
| Bai3 | 4.6590962 | 2.3912654 | -0.48675338 |
| Snai3 | 4.042445 | 2.1091752 | -0.4782427 |
| 9330154K18Rik | 6.495067 | 3.4037662 | -0.47594595 |
| Prss56 | 7.5221634 | 3.969582 | -0.47228187 |
| Dtl | 5.5660005 | 2.9374049 | -0.47225932 |
| Gm2518 | 4.194664 | 2.2138062 | -0.47223277 |
| Agr2 | 6.4094253 | 3.3876734 | -0.47145442 |
| Eif4g3 | 4.0552316 | 2.1472232 | -0.47050541 |
| 9130204L05Rik | 4.030237 | 2.1404972 | -0.46889049 |
| Olfr1500 | 6.4071875 | 3.4033356 | -0.46882535 |
| Scnn1b | 3.8758435 | 2.077741 | -0.46392547 |
| AI747448 | 3.8584511 | 2.0792747 | -0.46111156 |
| Scnn1g | 3.9017239 | 2.108066 | -0.45970908 |
| Kbtbd12 | 3.948622 | 2.138823 | -0.45833686 |
| C1qa | 3.9156299 | 2.1273663 | -0.45669883 |
| Cxcr1 | 3.9328494 | 2.1386437 | -0.45621012 |
| Prdm1 | 3.9780588 | 2.1867344 | -0.45030114 |
| 1700123I01Rik | 3.889217 | 2.144485 | -0.44860752 |
| Txndc8 | 3.8820293 | 2.1423843 | -0.44812774 |
| Vmn1r56 | 3.8199887 | 2.1099834 | -0.44764669 |
| Ccna1 | 3.9183588 | 2.1805663 | -0.4435001 |
| Mybpc3 | 3.9065664 | 2.1844454 | -0.44082727 |
| Ms4a4a | 4.0488243 | 2.2686043 | -0.43968813 |
| Gm16486 | 6.2607384 | 3.510353 | -0.43930687 |
| Olfr1351 | 4.1025047 | 2.3063257 | -0.43782497 |
| Prkd3 | 5.811213 | 3.2763615 | -0.43620007 |
| 4930437M23Rik | 3.7544909 | 2.1170614 | -0.43612557 |
| Olfr1121 | 3.929918 | 2.2161634 | -0.43607897 |
| Spata17 | 4.010835 | 2.2656064 | -0.4351285 |
| Ror1 | 5.6363554 | 3.1885529 | -0.43428818 |
| Aldh1l1 | 3.7223668 | 2.1078339 | -0.43373826 |
| Rex2 | 3.6760347 | 2.0837517 | -0.43315233 |
| 1700012A16Rik | 3.7534332 | 2.1299615 | -0.4325298 |
| 4930579G18Rik | 3.8782582 | 2.220545 | -0.42743755 |
| Nkx2-9 | 3.6467752 | 2.094918 | -0.42554233 |
| Ngp | 3.590034 | 2.068805 | -0.42373666 |
| Tpm3 | 3.801676 | 2.193893 | -0.42291426 |
| Ptpru | 3.6675496 | 2.1234443 | -0.42101825 |
| Cmtm2a | 3.6671245 | 2.1315606 | -0.41873787 |
| Olfr1037 | 3.813589 | 2.2222254 | -0.41728765 |
| Olfr601 | 3.6474888 | 2.138458 | -0.41371773 |
| Uggt2 | 3.5893903 | 2.1060517 | -0.41325642 |
| 4930455J16Rik | 4.417872 | 2.597135 | -0.41212987 |
| Olah | 6.033617 | 3.5620544 | -0.409632 |
| Gfpt1 | 3.6222947 | 2.1391077 | -0.40946061 |
| 2700081L22Rik | 3.5408618 | 2.0946302 | -0.40844057 |
| Hsf5 | 3.8316069 | 2.2750523 | -0.40624068 |
| Chi3l4 | 3.466053 | 2.066873 | -0.40368108 |
| Lactbl1 | 3.520311 | 2.1033733 | -0.40250356 |
| Cyp2d34 | 3.496053 | 2.0915956 | -0.40172658 |
| Ccdc79 | 3.5982711 | 2.1574159 | -0.40042986 |
| Clcn1 | 3.4806597 | 2.0940852 | -0.39836543 |
| Ccdc64 | 3.7108853 | 2.2357721 | -0.39750978 |
| Iqch | 3.6304662 | 2.1878436 | -0.39736566 |
| 4933439N14Rik | 3.7418466 | 2.2594047 | -0.39617923 |
| 2500004C02Rik | 3.4476123 | 2.0822496 | -0.39603139 |
| 4930547C10Rik | 3.6151087 | 2.1853726 | -0.3954891 |
| AA408954 | 3.4369009 | 2.0786886 | -0.39518518 |
| Gm9902 | 3.7050142 | 2.2423358 | -0.39478348 |
| Olfr474 | 3.8809178 | 2.3536086 | -0.3935433 |
| Olfr497 | 3.4530542 | 2.1007118 | -0.3916366 |
| Il1rapl2 | 3.6407838 | 2.215353 | -0.39151756 |
| Defa23 | 3.4462013 | 2.1004226 | -0.39051076 |
| Nbeal1 | 5.2419024 | 3.196473 | -0.39020746 |
| Mmp1a | 5.679878 | 3.4639683 | -0.39013333 |
| Slc44a5 | 3.5831456 | 2.1918325 | -0.38829377 |
| 4930483K19Rik | 3.4599583 | 2.1341612 | -0.38318297 |
| Pla2g1b | 4.981492 | 3.0849776 | -0.38071212 |
| Hfm1 | 3.5857625 | 2.2319853 | -0.37754235 |
| Odf3 | 3.6153436 | 2.251596 | -0.37721106 |
| Neurog3 | 8.749576 | 5.452244 | -0.3768562 |
| Trav12-1 | 3.6520605 | 2.2758846 | -0.37682177 |
| AI848285 | 3.8987494 | 2.429812 | -0.37677143 |
| Crygn | 3.4273963 | 2.1417327 | -0.37511379 |
| Gm10450 | 3.3232536 | 2.0767362 | -0.37508946 |
| Gjd4 | 3.3905573 | 2.1213272 | -0.37434262 |
| LOC100861598 | 3.6297452 | 2.2712622 | -0.37426401 |
| Hs3st4 | 3.3171308 | 2.0810418 | -0.372638 |
| Klf8 | 3.526027 | 2.2240372 | -0.36925123 |
| Ivl | 3.2645783 | 2.0671272 | -0.36680116 |
| Ccdc64b | 3.3524752 | 2.1260874 | -0.36581562 |
| Mip | 3.2516963 | 2.0700455 | -0.36339519 |
| Pcdh15 | 3.5237687 | 2.2581153 | -0.35917607 |
| 5830444B04Rik | 3.3855183 | 2.1733356 | -0.35804937 |
| Gm10768 | 3.3451388 | 2.1540082 | -0.35607808 |
| Trp63 | 5.404841 | 3.4844432 | -0.35531069 |
| Rnf180 | 3.274458 | 2.1116676 | -0.35510927 |
| Magea3 | 3.2543583 | 2.098907 | -0.35504735 |
| LOC100041550 | 3.2674727 | 2.1125455 | -0.35346193 |
| Fabp1 | 3.417554 | 2.2118995 | -0.35278287 |
| Rxfp4 | 3.2809784 | 2.1334229 | -0.34976015 |
| Il1rl2 | 3.246375 | 2.1129324 | -0.349141 |
| 4930444G20Rik | 3.260392 | 2.1235137 | -0.34869375 |
| 2310015D24Rik | 3.2293286 | 2.107582 | -0.34736217 |
| Pom121l2 | 4.778116 | 3.1184223 | -0.34735316 |
| Cd177 | 3.3649871 | 2.2000902 | -0.34618168 |
| Olfr632 | 3.679048 | 2.4058537 | -0.34606624 |
| Vmn1r121 | 3.174033 | 2.0832102 | -0.34367091 |
| Rsph6a | 3.3712199 | 2.2147593 | -0.34303921 |
| Pnma1 | 3.949033 | 2.596856 | -0.34240712 |
| Krtap28-10 | 4.838746 | 3.1911309 | -0.34050457 |
| Olfr873 | 3.3032393 | 2.1823976 | -0.33931593 |
| Ccl25 | 3.2540765 | 2.151823 | -0.33873005 |
| 4932425I24Rik | 3.815295 | 2.524757 | -0.33825379 |
| Olfr113 | 4.3567524 | 2.8844123 | -0.33794441 |
| Gm5432 | 3.396185 | 2.2601705 | -0.33449724 |
| Vmn1r28 | 2.5402243 | 3.811312 | 0.500384041 |
| Pkd2l2 | 2.9855072 | 4.4866757 | 0.502818583 |
| B3gat2 | 2.177665 | 3.2907581 | 0.511140648 |
| Hif3a | 2.776981 | 4.206426 | 0.51474785 |
| Stxbp6 | 2.842564 | 4.314596 | 0.5178536 |
| Musk | 2.1924708 | 3.331255 | 0.519406781 |
| Sgk2 | 2.1575446 | 3.2799435 | 0.520220486 |
| 2610206C17Rik | 2.2987483 | 3.4959927 | 0.520824485 |
| Zc3h6 | 3.1126485 | 4.7472086 | 0.525134817 |
| Sox3 | 2.444526 | 3.734141 | 0.527552172 |
| Ppp1r2 | 5.273652 | 8.059172 | 0.528195641 |
| Zfp874b | 2.4323704 | 3.7259743 | 0.531828499 |
| Atp13a5 | 2.2313206 | 3.4194582 | 0.532481796 |
| Cd274 | 2.759736 | 4.253599 | 0.541306487 |
| Lce3c | 2.2501645 | 3.4711413 | 0.542616684 |
| Gm5796 | 2.095458 | 3.2349153 | 0.543774822 |
| Snca | 2.3715034 | 3.6615634 | 0.54398404 |
| Olfr836 | 2.1490917 | 3.3198085 | 0.54474958 |
| Speer5-ps1 | 2.1311276 | 3.2921486 | 0.544791874 |
| 6720407P12Rik | 3.194312 | 4.98651 | 0.561059158 |
| Btbd7 | 2.8163714 | 4.4059024 | 0.564389697 |
| Fam19a5 | 2.0931377 | 3.2892013 | 0.571421364 |
| Rasd1 | 2.1473644 | 3.3827965 | 0.575324849 |
| Gm6559 | 2.1102223 | 3.3430414 | 0.584212905 |
| Ldb3 | 2.7837696 | 4.424507 | 0.589394108 |
| Srsf12 | 2.1623847 | 3.438515 | 0.590149523 |
| A930006K02Rik | 2.1503396 | 3.4231973 | 0.591933339 |
| Fancm | 2.8156056 | 4.5013537 | 0.598715992 |
| Yod1 | 2.8251972 | 4.534269 | 0.60493894 |
| Gm10554 | 2.8593428 | 4.5940394 | 0.606676681 |
| 1700040N02Rik | 2.332089 | 3.7830389 | 0.622167464 |
| Tmprss11f | 2.2978761 | 3.7407587 | 0.627920104 |
| Fpr-rs6 | 2.1226175 | 3.473301 | 0.636329202 |
| 4833420D23Rik | 2.076229 | 3.4140553 | 0.644353922 |
| Klra9 | 2.5865164 | 4.2649107 | 0.648901472 |
| Pnlip | 2.1391582 | 3.5280645 | 0.649277038 |
| Cnga2 | 2.2006578 | 3.636188 | 0.652318684 |
| Tcf4 | 2.2421043 | 3.709677 | 0.654551485 |
| Zfp85-rs1 | 2.093985 | 3.4682913 | 0.656311435 |
| 5033403F01Rik | 2.1610985 | 3.6004145 | 0.66601129 |
| Lipc | 2.1413317 | 3.5679965 | 0.666251193 |
| Zfp446 | 2.1503046 | 3.584693 | 0.66706289 |
| Bach2 | 2.6730514 | 4.470415 | 0.672401436 |
| 2410002O22Rik | 2.2063766 | 3.6925423 | 0.67357753 |
| Gm10561 | 2.435295 | 4.1110687 | 0.688119386 |
| Mrpl47 | 2.315041 | 3.925583 | 0.695686167 |
| Sox30 | 2.1573064 | 3.6645632 | 0.698675348 |
| Nbea | 2.6196184 | 4.4955144 | 0.716095138 |
| 2810471M01Rik | 2.211711 | 3.8093061 | 0.722334473 |
| 5031434C07Rik | 2.3668678 | 4.081982 | 0.724634557 |
| Cep72 | 2.0733292 | 3.5944698 | 0.733670562 |
| Klra1 | 2.183711 | 3.802447 | 0.741277577 |
| Acp1 | 2.305737 | 4.0409665 | 0.752570436 |
| Gm9725 | 2.1391714 | 3.7603104 | 0.75783502 |
| Fam70b | 2.1830056 | 3.8507977 | 0.763988924 |
| Ece2 | 2.4211013 | 4.2791557 | 0.767441825 |
| Gm12758 | 2.3732188 | 4.199936 | 0.769721359 |
| Per3 | 2.6405284 | 4.6973515 | 0.778943752 |
| Rbm41 | 2.2752693 | 4.0615997 | 0.78510724 |
| Jph1 | 2.0947685 | 3.7554545 | 0.792777818 |
| Kitl | 2.674532 | 4.7956543 | 0.793081668 |
| Popdc2 | 2.0623372 | 3.7475424 | 0.817133687 |
| Itga1 | 2.1159585 | 3.854554 | 0.821658601 |
| Lyst | 2.1176054 | 3.8588636 | 0.822276993 |
| Dgkk | 2.169744 | 3.9590101 | 0.824643875 |
| 4931406P16Rik | 2.134399 | 3.9023957 | 0.828334674 |
| Gm10375 | 2.1021214 | 3.8536372 | 0.833213439 |
| Luc7l3 | 2.1742697 | 3.9865026 | 0.83349039 |
| Yipf6 | 2.4983795 | 4.593412 | 0.838556552 |
| Bicd1 | 2.0948758 | 3.8578506 | 0.841565309 |
| Ostb | 2.2380977 | 4.138494 | 0.849112306 |
| Crebbp | 2.1352015 | 3.9580855 | 0.853729262 |
| Gm3161 | 2.1860466 | 4.052736 | 0.853911074 |
| Thoc1 | 2.3011732 | 4.274261 | 0.857426899 |
| Gm807 | 2.2368324 | 4.163067 | 0.86114391 |
| Sec14l3 | 2.076511 | 3.9256337 | 0.890495018 |
| Pbrm1 | 2.2527454 | 4.268445 | 0.894774705 |
| Il17re | 2.1253014 | 4.108692 | 0.933227918 |
| Tspan13 | 2.25259 | 4.388052 | 0.948002965 |
| Abhd15 | 2.224064 | 4.3828187 | 0.970635153 |
| Csprs | 2.0828261 | 4.2065682 | 1.019644463 |
| A230006K03Rik | 2.2355769 | 4.605414 | 1.060056176 |
| A630081J09Rik | 2.174829 | 4.4831977 | 1.061402391 |
| Dopey1 | 2.0958662 | 4.583559 | 1.186952106 |
| Spaca1 | 2.1858408 | 5.680703 | 1.598864016 |
| Fbxo30 | 2.230653 | 7.337605 | 2.289442598 |
| AU020094 | 2.2394578 | 8.159487 | 2.64351005 |
| Prpmp5 | 2.2372317 | 8.615651 | 2.851032059 |

(B) Genes gene expression profiles in MSCs cocultured TCs compared with MSCs.

| GeneSymbol | MSCs | MSCs cocultured TCs | Fold Change |
| --- | --- | --- | --- |
| 4930435E12Rik | 6.8268404 | 2.0802398 | -0.695285128 |
| Gm10487 | 6.600509 | 2.1191456 | -0.678942094 |
| Olfr1500 | 6.4071875 | 2.0589964 | -0.678642712 |
| Hrasls | 6.436921 | 2.141373 | -0.667329613 |
| 4930451I11Rik | 6.049219 | 2.0977826 | -0.653214307 |
| Ermap | 5.751102 | 2.092471 | -0.63616173 |
| Ror1 | 5.6363554 | 2.0829847 | -0.630437658 |
| Mmp1a | 5.679878 | 2.103681 | -0.629625672 |
| Vmn2r37 | 5.5317235 | 2.103701 | -0.619702431 |
| Sycp3 | 5.473989 | 2.1061685 | -0.615240641 |
| Tlx3 | 5.407043 | 2.0973034 | -0.612116382 |
| Dtl | 5.5660005 | 2.195966 | -0.605467876 |
| 1700013N18Rik | 5.402881 | 2.1409936 | -0.60373112 |
| Rsph10b2 | 5.158323 | 2.1258252 | -0.587884435 |
| Igh-VJ558 | 5.359541 | 2.22146 | -0.585513013 |
| Mgam | 5.173423 | 2.2106516 | -0.572690731 |
| Krtap28-10 | 4.838746 | 2.072321 | -0.571723542 |
| Rnase11 | 4.753851 | 2.0686133 | -0.564855251 |
| Krt26 | 5.2349377 | 2.2805529 | -0.564359114 |
| Olfr1155 | 4.860658 | 2.119642 | -0.563918712 |
| Olfr366 | 5.0788374 | 2.2267737 | -0.561558379 |
| Hrk | 5.1569633 | 2.2627397 | -0.561226333 |
| Pom121l2 | 4.778116 | 2.153718 | -0.549253723 |
| 1700110I01Rik | 4.668014 | 2.123818 | -0.5450275 |
| Olfr243 | 4.584425 | 2.135035 | -0.534285107 |
| Patl2 | 4.512514 | 2.1023731 | -0.534101589 |
| Ncam2 | 4.4337535 | 2.0691936 | -0.533308832 |
| Zfp874a | 4.367638 | 2.0592895 | -0.528511864 |
| 2410004P03Rik | 4.760647 | 2.2531629 | -0.526710781 |
| Gfi1 | 4.3826065 | 2.0830944 | -0.524690524 |
| Actrt2 | 4.4131794 | 2.1244671 | -0.518608489 |
| Olfr113 | 4.3567524 | 2.1200237 | -0.513393577 |
| B4galnt3 | 4.349327 | 2.1179094 | -0.513048938 |
| Cd164l2 | 4.4725447 | 2.1859899 | -0.51124247 |
| Gm5941 | 4.388052 | 2.2475798 | -0.487795541 |
| Klrc2 | 4.318441 | 2.219754 | -0.485982557 |
| Slc10a2 | 4.0603304 | 2.092869 | -0.484556971 |
| 9330154K18Rik | 6.495067 | 3.3592994 | -0.482792187 |
| Ccdc147 | 4.352293 | 2.2615788 | -0.480370738 |
| Npffr1 | 4.2261415 | 2.2066448 | -0.477858278 |
| Kbtbd12 | 3.948622 | 2.0678387 | -0.476313838 |
| Olfr1351 | 4.1025047 | 2.150562 | -0.475792922 |
| Eif4g3 | 4.0552316 | 2.1588905 | -0.46762831 |
| Agr2 | 6.4094253 | 3.4156883 | -0.467083531 |
| Prss56 | 7.5221634 | 4.042914 | -0.462533079 |
| Resp18 | 4.07533 | 2.1938548 | -0.461674318 |
| 2610018G03Rik | 5.5079255 | 2.9809456 | -0.458789775 |
| Cxcr1 | 3.9328494 | 2.1290803 | -0.458641793 |
| Scnn1g | 3.9017239 | 2.1231055 | -0.455854501 |
| Txndc8 | 3.8820293 | 2.1167364 | -0.454734564 |
| AI747448 | 3.8584511 | 2.1056285 | -0.454281408 |
| 9130204L05Rik | 4.030237 | 2.2068455 | -0.45242786 |
| C1qa | 3.9156299 | 2.1483755 | -0.45133336 |
| Ccna1 | 3.9183588 | 2.1572664 | -0.449446437 |
| Csprs | 4.631054 | 2.5639515 | -0.446356812 |
| Gm2518 | 4.194664 | 2.3281884 | -0.444964269 |
| Vmn1r56 | 3.8199887 | 2.132271 | -0.441812223 |
| Prdm1 | 3.9780588 | 2.220605 | -0.441786783 |
| 4930579G18Rik | 3.8782582 | 2.165822 | -0.441547755 |
| Mybpc3 | 3.9065664 | 2.186251 | -0.440365074 |
| Ms4a4a | 4.0488243 | 2.28241 | -0.436278329 |
| Rex2 | 3.6760347 | 2.0761719 | -0.435214281 |
| 4930437M23Rik | 3.7544909 | 2.1342547 | -0.431546178 |
| Spata17 | 4.010835 | 2.2859106 | -0.430066158 |
| Aldh1l1 | 3.7223668 | 2.129115 | -0.428021172 |
| 1700012A16Rik | 3.7534332 | 2.1547565 | -0.425923845 |
| Gfpt1 | 3.6222947 | 2.0842688 | -0.424599881 |
| Scnn1b | 3.8758435 | 2.240255 | -0.421995496 |
| Nkx2-9 | 3.6467752 | 2.1137059 | -0.420390404 |
| Ptpru | 3.6675496 | 2.136268 | -0.417521715 |
| Hfm1 | 3.5857625 | 2.0944345 | -0.415902615 |
| Ngp | 3.590034 | 2.1006618 | -0.414862979 |
| 4930455J16Rik | 4.417872 | 2.5901458 | -0.413711896 |
| Uggt2 | 3.5893903 | 2.1057286 | -0.413346439 |
| Olfr601 | 3.6474888 | 2.1451976 | -0.411869997 |
| Olfr1121 | 3.929918 | 2.3114371 | -0.411835794 |
| Hsf5 | 3.8316069 | 2.2630389 | -0.409376024 |
| 2700081L22Rik | 3.5408618 | 2.097559 | -0.407613423 |
| Olfr474 | 3.8809178 | 2.306371 | -0.40571506 |
| Lactbl1 | 3.520311 | 2.0929835 | -0.405454944 |
| Olah | 6.033617 | 3.5997248 | -0.403388581 |
| Trp63 | 3.7342207 | 2.2418945 | -0.399635244 |
| Olfr1037 | 3.813589 | 2.291107 | -0.399225506 |
| Neurog3 | 8.749576 | 5.2591715 | -0.398922702 |
| Chi3l4 | 3.466053 | 2.0889726 | -0.397305061 |
| 4930547C10Rik | 3.6151087 | 2.1874135 | -0.394924556 |
| Snai3 | 4.042445 | 2.4564612 | -0.392332808 |
| Defa23 | 3.4462013 | 2.094853 | -0.39212692 |
| Ccdc79 | 3.5982711 | 2.1882718 | -0.39185466 |
| Slc44a5 | 3.5831456 | 2.1871293 | -0.389606356 |
| Gm9902 | 3.7050142 | 2.2649634 | -0.388676189 |
| Crygn | 3.4273963 | 2.0961585 | -0.388410818 |
| Tpm3 | 3.801676 | 2.3262246 | -0.388105509 |
| Olfr497 | 3.4530542 | 2.1143696 | -0.387681317 |
| Pcdh15 | 3.5237687 | 2.1578183 | -0.387639064 |
| AA408954 | 3.4369009 | 2.1052442 | -0.387458568 |
| Il1rapl2 | 3.6407838 | 2.233386 | -0.386564508 |
| 2500004C02Rik | 3.4476123 | 2.1163309 | -0.386145913 |
| Trav12-1 | 3.6520605 | 2.2433677 | -0.385725483 |
| Cyp2d34 | 3.496053 | 2.159805 | -0.382216173 |
| Iqch | 3.6304662 | 2.2492805 | -0.380443068 |
| Cmtm2a | 3.6671245 | 2.2750175 | -0.379618145 |
| Gjd4 | 3.3905573 | 2.116872 | -0.375656621 |
| 4933439N14Rik | 3.7418466 | 2.338482 | -0.375046 |
| AI848285 | 3.8987494 | 2.4406998 | -0.373978794 |
| Bai3 | 4.6590962 | 2.9378364 | -0.369440708 |
| Odf3 | 3.6153436 | 2.2834086 | -0.368411733 |
| Cd177 | 3.3649871 | 2.1279066 | -0.367633059 |
| Gm16486 | 6.2607384 | 3.97341 | -0.365344829 |
| Klf8 | 3.526027 | 2.239871 | -0.364760678 |
| Gm5432 | 3.396185 | 2.161789 | -0.363465477 |
| Ccdc64 | 3.7108853 | 2.3729985 | -0.360530356 |
| Ivl | 3.2645783 | 2.0914776 | -0.359342185 |
| 4930483K19Rik | 3.4599583 | 2.2179527 | -0.358965482 |
| Rsph6a | 3.3712199 | 2.1627502 | -0.358466589 |
| Ccdc64b | 3.3524752 | 2.1529255 | -0.357810164 |
| Mip | 3.2516963 | 2.0933113 | -0.356240218 |
| Magea3 | 3.2543583 | 2.0965269 | -0.355778711 |
| LOC100861598 | 3.6297452 | 2.3446376 | -0.354048984 |
| 4930444G20Rik | 3.260392 | 2.1136556 | -0.35171734 |
| Rnf180 | 3.274458 | 2.1306956 | -0.349298235 |
| Rxfp4 | 3.2809784 | 2.1412609 | -0.347371229 |
| Gm10768 | 3.3451388 | 2.183604 | -0.347230674 |
| Prkd3 | 5.811213 | 3.7936282 | -0.347188238 |
| LOC100041550 | 3.2674727 | 2.1366472 | -0.346085677 |
| Il1rl2 | 3.246375 | 2.1275015 | -0.34465319 |
| Pnma1 | 3.949033 | 2.5923076 | -0.343558892 |
| Vmn1r121 | 3.174033 | 2.0883927 | -0.342038126 |
| 2310015D24Rik | 3.2293286 | 2.1276317 | -0.341153545 |
| Clcn1 | 3.4806597 | 2.296232 | -0.340288279 |
| Pla2g1b | 4.981492 | 3.28917 | -0.339721915 |
| Fabp1 | 3.417554 | 2.259246 | -0.338928953 |
| Olfr632 | 3.679048 | 2.4333131 | -0.338602513 |
| Gm10450 | 3.3232536 | 2.1980453 | -0.338586348 |
| Hs3st4 | 3.3171308 | 2.1953678 | -0.338172676 |
| Olfr873 | 3.3032393 | 2.1921444 | -0.336365246 |
| 4932425I24Rik | 3.815295 | 2.5363007 | -0.335228154 |
| Ccl25 | 3.2540765 | 2.1664784 | -0.334226347 |
| 5830444B04Rik | 3.3855183 | 2.256562 | -0.333466311 |
| Il17re | 2.1253014 | 3.2053998 | 0.508209518 |
| Cd274 | 2.759736 | 4.165384 | 0.509341473 |
| Speer5-ps1 | 2.406009 | 3.634553 | 0.510614881 |
| 4833420D23Rik | 2.076229 | 3.1507144 | 0.517517769 |
| 1700123I01Rik | 2.5174112 | 3.8309112 | 0.52176617 |
| 2810471M01Rik | 2.211711 | 3.3780584 | 0.527350725 |
| Gm10554 | 2.8593428 | 4.3758287 | 0.530361697 |
| Zc3h6 | 3.1126485 | 4.7687907 | 0.532068494 |
| Luc7l3 | 2.1742697 | 3.3501527 | 0.540817452 |
| Ppp1r2 | 5.273652 | 8.131607 | 0.541930905 |
| Sgk2 | 2.1575446 | 3.34568 | 0.550688686 |
| Thoc1 | 2.3011732 | 3.5855343 | 0.558133173 |
| Olfr836 | 2.1490917 | 3.3526435 | 0.560028127 |
| Fam70b | 2.1830056 | 3.4106462 | 0.562362552 |
| Fpr-rs6 | 2.1226175 | 3.318167 | 0.563243024 |
| Lyst | 2.1176054 | 3.329803 | 0.572437906 |
| Tcf4 | 2.2421043 | 3.5310597 | 0.574886458 |
| Cnga2 | 2.2006578 | 3.4934406 | 0.587452897 |
| Lipc | 2.1413317 | 3.4123375 | 0.593558579 |
| Vmn1r28 | 2.5402243 | 4.052983 | 0.595521703 |
| Yipf6 | 2.4983795 | 3.987705 | 0.596116603 |
| 2410002O22Rik | 2.2063766 | 3.5451715 | 0.606784399 |
| A230006K03Rik | 2.2355769 | 3.5930452 | 0.607211633 |
| 6720407P12Rik | 3.194312 | 5.1376786 | 0.608383464 |
| Zfp446 | 2.1503046 | 3.472195 | 0.61474565 |
| Pnlip | 2.1391582 | 3.4662843 | 0.620396425 |
| Sec14l3 | 2.076511 | 3.372192 | 0.623970208 |
| Gm6559 | 2.1102223 | 3.433424 | 0.627043748 |
| Gm5796 | 2.095458 | 3.4109702 | 0.627792206 |
| Cep72 | 2.0733292 | 3.390312 | 0.635201974 |
| Kitl | 2.674532 | 4.377865 | 0.636871423 |
| Fancm | 2.8156056 | 4.617351 | 0.639913985 |
| Rbm41 | 2.2752693 | 3.734551 | 0.641366585 |
| Per3 | 2.6405284 | 4.35175 | 0.64806029 |
| Nbeal1 | 2.2009544 | 3.633991 | 0.651097815 |
| Yod1 | 2.8251972 | 4.6672583 | 0.652011513 |
| Gm10561 | 2.435295 | 4.0668483 | 0.669961257 |
| Klra9 | 2.5865164 | 4.324294 | 0.671860267 |
| Ostb | 2.2380977 | 3.770674 | 0.684767381 |
| Gm10375 | 2.1021214 | 3.5454457 | 0.686603685 |
| 2610206C17Rik | 2.2987483 | 3.9041224 | 0.698368804 |
| Hif3a | 2.776981 | 4.719483 | 0.699501365 |
| A930006K02Rik | 2.1503396 | 3.6821759 | 0.712369479 |
| A630081J09Rik | 2.174829 | 3.7423418 | 0.720752206 |
| Gm9725 | 2.1391714 | 3.6891892 | 0.724587941 |
| Btbd7 | 2.8163714 | 4.8639584 | 0.727030178 |
| Sox3 | 2.444526 | 4.2334533 | 0.73180948 |
| Jph1 | 2.0947685 | 3.6501093 | 0.742488156 |
| 5033403F01Rik | 2.1610985 | 3.770365 | 0.744652083 |
| Acp1 | 2.305737 | 4.0374784 | 0.751057644 |
| 1700040N02Rik | 2.332089 | 4.103784 | 0.759702996 |
| Pkd2l2 | 2.9855072 | 5.2711782 | 0.765588842 |
| Zfp85-rs1 | 2.093985 | 3.707372 | 0.770486417 |
| Rasd1 | 2.1473644 | 3.833295 | 0.78511621 |
| Gm12758 | 2.3732188 | 4.2687283 | 0.798708278 |
| Tmprss11f | 2.2978761 | 4.15259 | 0.807142691 |
| Sox30 | 2.1573064 | 3.9036748 | 0.809513382 |
| Pbrm1 | 2.2527454 | 4.088317 | 0.81481538 |
| 5031434C07Rik | 2.3668678 | 4.3160305 | 0.823519886 |
| Fam19a5 | 2.0931377 | 3.8401606 | 0.834643082 |
| Itga1 | 2.1159585 | 3.888968 | 0.837922625 |
| Dgkk | 2.169744 | 4.0057077 | 0.846166045 |
| Mrpl47 | 2.315041 | 4.296132 | 0.855747695 |
| Lce3c | 2.2501645 | 4.218047 | 0.874550505 |
| Ldb3 | 2.7837696 | 5.2252727 | 0.877049272 |
| 4931406P16Rik | 2.134399 | 4.0153804 | 0.881269809 |
| Gm807 | 2.2368324 | 4.208757 | 0.881570117 |
| Zfp874b | 2.6149125 | 4.9283714 | 0.884717519 |
| Srsf12 | 2.1623847 | 4.1286283 | 0.909294077 |
| Musk | 2.1924708 | 4.1877775 | 0.910072189 |
| Klra1 | 2.183711 | 4.177766 | 0.91314968 |
| Gm3161 | 2.1860466 | 4.199839 | 0.921202869 |
| Nbea | 2.6196184 | 5.047906 | 0.926962339 |
| Bach2 | 2.6730514 | 5.1759214 | 0.936334408 |
| Stxbp6 | 2.842564 | 5.561162 | 0.956389372 |
| B3gat2 | 2.177665 | 4.274131 | 0.962712814 |
| Crebbp | 2.1352015 | 4.2000346 | 0.967043672 |
| Dopey1 | 2.0958662 | 4.1268044 | 0.969020923 |
| Tspan13 | 2.25259 | 4.443609 | 0.972666575 |
| Spaca1 | 2.1858408 | 4.3843865 | 1.005812363 |
| Abhd15 | 2.224064 | 4.544505 | 1.043333735 |
| Bicd1 | 2.0948758 | 4.282593 | 1.044318331 |
| Ece2 | 2.4211013 | 5.080883 | 1.098583401 |
| Atp13a5 | 2.2313206 | 4.8051367 | 1.153494527 |
| Popdc2 | 2.0623372 | 4.683003 | 1.270726145 |
| Snca | 2.3715034 | 5.6984863 | 1.402900329 |
| Fbxo30 | 2.230653 | 7.188835 | 2.222749123 |
| AU020094 | 2.2394578 | 7.954835 | 2.55212543 |
| Prpmp5 | 2.2372317 | 8.497708 | 2.798313782 |

(C) Genes gene expression profiles in MSCs cocultured TCs and stimulated with 1μg/ml LPS compared with MSCs.

| GeneSymbol | MSCs | MSCs cocultured TCs and stimulated with 1μg/ml LPS | Fold Change |
| --- | --- | --- | --- |
| 4930435E12Rik | 6.8268404 | 2.0733368 | -0.696296284 |
| Hrasls | 6.436921 | 2.1049495 | -0.672988141 |
| Gm10487 | 6.600509 | 2.2525194 | -0.658735501 |
| 4930451I11Rik | 6.049219 | 2.0934331 | -0.653933326 |
| 2610018G03Rik | 5.5079255 | 2.0729237 | -0.623647106 |
| Vmn2r37 | 5.5317235 | 2.113878 | -0.617862679 |
| Sycp3 | 5.473989 | 2.1013966 | -0.616112382 |
| Tlx3 | 5.407043 | 2.1145282 | -0.608930759 |
| Igh-VJ558 | 5.359541 | 2.1677532 | -0.595533797 |
| Rsph10b2 | 5.158323 | 2.0981436 | -0.59325083 |
| Prss56 | 7.5221634 | 3.14599 | -0.581770585 |
| Olfr366 | 5.0788374 | 2.1275342 | -0.581098186 |
| Krt26 | 5.2349377 | 2.2047362 | -0.578841941 |
| 9330154K18Rik | 6.495067 | 2.7857423 | -0.571098758 |
| Krtap28-10 | 4.838746 | 2.0787795 | -0.570388795 |
| Trp63 | 5.404841 | 2.37742 | -0.560131371 |
| Pom121l2 | 4.778116 | 2.105237 | -0.55940019 |
| Rnase11 | 4.753851 | 2.0986125 | -0.558544746 |
| Olfr1500 | 6.4071875 | 2.890347 | -0.548889899 |
| Olfr243 | 4.584425 | 2.106568 | -0.540494609 |
| 2410004P03Rik | 4.760647 | 2.1882625 | -0.540343466 |
| Patl2 | 4.512514 | 2.088483 | -0.537179718 |
| 1700013N18Rik | 5.402881 | 2.502141 | -0.536887635 |
| Hrk | 5.1569633 | 2.4124823 | -0.53218936 |
| Csprs | 4.631054 | 2.1665092 | -0.532177945 |
| Ncam2 | 4.4337535 | 2.0761838 | -0.531732244 |
| Mgam | 5.173423 | 2.4508438 | -0.526262631 |
| Gfi1 | 4.3826065 | 2.0786808 | -0.525697596 |
| Zfp874a | 4.367638 | 2.0765233 | -0.52456607 |
| Cd164l2 | 4.4725447 | 2.1359131 | -0.52243896 |
| Olfr113 | 4.3567524 | 2.102963 | -0.517309499 |
| B4galnt3 | 4.349327 | 2.1021526 | -0.516671752 |
| Ermap | 5.751102 | 2.7908483 | -0.514728082 |
| Actrt2 | 4.4131794 | 2.1417725 | -0.514687189 |
| Ccdc147 | 4.352293 | 2.1516984 | -0.505617292 |
| Gm5941 | 4.388052 | 2.1821725 | -0.502701313 |
| Agr2 | 6.4094253 | 3.2048092 | -0.499984936 |
| Dtl | 5.5660005 | 2.7926445 | -0.498267293 |
| Klrc2 | 4.318441 | 2.166894 | -0.498223086 |
| Npffr1 | 4.2261415 | 2.138339 | -0.494020965 |
| Snai3 | 4.042445 | 2.0903084 | -0.482909873 |
| 9130204L05Rik | 4.030237 | 2.1032305 | -0.478137266 |
| Scnn1b | 3.8758435 | 2.0720656 | -0.465389766 |
| Spata17 | 4.010835 | 2.150794 | -0.463754056 |
| Hsf5 | 3.8316069 | 2.076111 | -0.458161796 |
| Gm16486 | 6.2607384 | 3.4030075 | -0.456452693 |
| 1700123I01Rik | 3.889217 | 2.1186469 | -0.455251044 |
| Mybpc3 | 3.9065664 | 2.129339 | -0.454933366 |
| Txndc8 | 3.8820293 | 2.1176412 | -0.45450149 |
| Ccna1 | 3.9183588 | 2.1376982 | -0.454440415 |
| C1qa | 3.9156299 | 2.1510243 | -0.450656892 |
| 4930579G18Rik | 3.8782582 | 2.1507432 | -0.445435789 |
| Olfr1121 | 3.929918 | 2.223757 | -0.434146718 |
| AI848285 | 3.8987494 | 2.207236 | -0.433860509 |
| Olfr474 | 3.8809178 | 2.2043748 | -0.431996524 |
| Aldh1l1 | 3.7223668 | 2.1155546 | -0.431664123 |
| Gm2518 | 4.194664 | 2.4004242 | -0.42774339 |
| Gm9902 | 3.7050142 | 2.1417403 | -0.421934658 |
| Pnma1 | 3.949033 | 2.2841625 | -0.421589412 |
| Olfr601 | 3.6474888 | 2.1102273 | -0.421457497 |
| Olfr1037 | 3.813589 | 2.2108867 | -0.420260888 |
| Ngp | 3.590034 | 2.0841906 | -0.419451014 |
| Slc10a2 | 4.0603304 | 2.3577871 | -0.419311517 |
| Uggt2 | 3.5893903 | 2.0877237 | -0.418362584 |
| Tpm3 | 3.801676 | 2.2206926 | -0.415864845 |
| 4930437M23Rik | 3.7544909 | 2.2006612 | -0.41385896 |
| Ptpru | 3.6675496 | 2.1509554 | -0.413517025 |
| 4930547C10Rik | 3.6151087 | 2.129918 | -0.410828781 |
| 4930455J16Rik | 4.417872 | 2.6032495 | -0.41074583 |
| Cxcr1 | 3.9328494 | 2.3270333 | -0.408308566 |
| Lactbl1 | 3.520311 | 2.0873094 | -0.407066762 |
| Ccdc79 | 3.5982711 | 2.1343958 | -0.406827407 |
| Cmtm2a | 3.6671245 | 2.1761181 | -0.406587341 |
| Trav12-1 | 3.6520605 | 2.1742697 | -0.404645761 |
| 2500004C02Rik | 3.4476123 | 2.0573287 | -0.403259845 |
| Odf3 | 3.6153436 | 2.1588821 | -0.402855623 |
| Slc44a5 | 3.5831456 | 2.1398082 | -0.402812936 |
| 4933439N14Rik | 3.7418466 | 2.2374315 | -0.402051516 |
| Defa23 | 3.4462013 | 2.0608273 | -0.402000313 |
| Hfm1 | 3.5857625 | 2.1490293 | -0.400677178 |
| Eif4g3 | 4.0552316 | 2.4345293 | -0.399657149 |
| Iqch | 3.6304662 | 2.1831975 | -0.398645414 |
| Prdm1 | 3.9780588 | 2.4052138 | -0.395380028 |
| Klf8 | 3.526027 | 2.1370764 | -0.393913773 |
| Clcn1 | 3.4806597 | 2.1097639 | -0.393860911 |
| Rex2 | 3.6760347 | 2.2385998 | -0.391028654 |
| Cyp2d34 | 3.496053 | 2.1303823 | -0.39063215 |
| Olfr632 | 3.679048 | 2.242928 | -0.390350982 |
| Mmp1a | 5.679878 | 3.4712722 | -0.388847401 |
| AA408954 | 3.4369009 | 2.1006286 | -0.388801522 |
| 1700110I01Rik | 4.668014 | 2.863859 | -0.386493057 |
| Ror1 | 5.6363554 | 3.4661372 | -0.385039276 |
| Crygn | 3.4273963 | 2.1093616 | -0.384558593 |
| Pla2g1b | 4.981492 | 3.0670667 | -0.384307613 |
| Olfr497 | 3.4530542 | 2.1274602 | -0.383890296 |
| LOC100861598 | 3.6297452 | 2.2395492 | -0.383000989 |
| Gjd4 | 3.3905573 | 2.0997863 | -0.380695822 |
| Neurog3 | 8.749576 | 5.4505267 | -0.377052477 |
| Nkx2-9 | 3.6467752 | 2.2788925 | -0.375093781 |
| Olfr1155 | 4.860658 | 3.0413194 | -0.37429883 |
| Gm5432 | 3.396185 | 2.1276248 | -0.373525058 |
| Olfr1351 | 4.1025047 | 2.590436 | -0.368572082 |
| Ivl | 3.2645783 | 2.066278 | -0.367061283 |
| Ms4a4a | 4.0488243 | 2.5641093 | -0.366702749 |
| Ccdc64b | 3.3524752 | 2.1235576 | -0.366570228 |
| 1700012A16Rik | 3.7534332 | 2.380201 | -0.365860301 |
| Gm10450 | 3.3232536 | 2.1103034 | -0.364988757 |
| Cd177 | 3.3649871 | 2.1399274 | -0.364060742 |
| Fabp1 | 3.417554 | 2.1748655 | -0.363619273 |
| Gfpt1 | 3.6222947 | 2.3058653 | -0.363424158 |
| AI747448 | 3.8584511 | 2.4567256 | -0.363287097 |
| Kbtbd12 | 3.948622 | 2.5141745 | -0.363277999 |
| Rsph6a | 3.3712199 | 2.1477964 | -0.362902313 |
| Gm10768 | 3.3451388 | 2.1313174 | -0.362861296 |
| Hs3st4 | 3.3171308 | 2.1195014 | -0.361043767 |
| Mip | 3.2516963 | 2.0790975 | -0.360611414 |
| Olfr873 | 3.3032393 | 2.1179726 | -0.358819508 |
| Rnf180 | 3.274458 | 2.103499 | -0.357603915 |
| 4930444G20Rik | 3.260392 | 2.0971265 | -0.356787006 |
| Magea3 | 3.2543583 | 2.0981147 | -0.355290811 |
| Il1rapl2 | 3.6407838 | 2.3497221 | -0.354610922 |
| Il1rl2 | 3.246375 | 2.0963926 | -0.354235848 |
| Ccl25 | 3.2540765 | 2.1018584 | -0.354084515 |
| Olah | 6.033617 | 3.8977523 | -0.35399408 |
| 5830444B04Rik | 3.3855183 | 2.1909952 | -0.352833154 |
| 2700081L22Rik | 3.5408618 | 2.2921486 | -0.352657989 |
| Bai3 | 4.6590962 | 3.018729 | -0.352078414 |
| Prkd3 | 5.811213 | 3.7772236 | -0.350011159 |
| 2310015D24Rik | 3.2293286 | 2.0997093 | -0.349800048 |
| LOC100041550 | 3.2674727 | 2.1246312 | -0.349763137 |
| Vmn1r121 | 3.174033 | 2.080357 | -0.344569826 |
| Pcdh15 | 3.36736 | 2.2126274 | -0.34291926 |
| Ccdc64 | 3.7108853 | 2.4387565 | -0.342810057 |
| 4932425I24Rik | 3.3822334 | 2.223472 | -0.342602435 |
| Resp18 | 4.07533 | 2.6796055 | -0.342481345 |
| Rxfp4 | 3.2809784 | 2.1578434 | -0.342317097 |
| Chi3l4 | 3.466053 | 2.2822208 | -0.341550519 |
| Sec14l3 | 3.3531084 | 2.2135582 | -0.3398489 |
| Vmn1r56 | 3.8199887 | 2.524576 | -0.339114275 |
| Scnn1g | 3.9017239 | 2.59541 | -0.33480429 |
| Gm5796 | 2.095458 | 3.1473033 | 0.501964392 |
| 1700040N02Rik | 2.332089 | 3.5304017 | 0.513836607 |
| Yipf6 | 2.4983795 | 3.8185382 | 0.528405993 |
| Gm10375 | 2.1021214 | 3.2148623 | 0.529341883 |
| Sox3 | 2.444526 | 3.7470517 | 0.532833645 |
| 4930483K19Rik | 3.4599583 | 5.3310328 | 0.540779494 |
| Hif3a | 2.776981 | 4.2836547 | 0.542558159 |
| A930006K02Rik | 2.1503396 | 3.3406699 | 0.553554564 |
| Ppp1r2 | 5.273652 | 8.207878 | 0.556393558 |
| Olfr836 | 2.1490917 | 3.3459044 | 0.556892337 |
| Vmn1r28 | 2.5402243 | 3.9561281 | 0.557393219 |
| 6720407P12Rik | 3.194312 | 4.9751077 | 0.557489594 |
| Gm3161 | 2.1860466 | 3.4262557 | 0.567329672 |
| Gm6559 | 2.1102223 | 3.3202329 | 0.573404328 |
| 2810471M01Rik | 2.211711 | 3.4990633 | 0.582061716 |
| Fam70b | 2.1830056 | 3.4539852 | 0.582215456 |
| Ostb | 2.2380977 | 3.5499394 | 0.586141391 |
| Zfp874b | 2.4323704 | 3.8950253 | 0.601329016 |
| Zc3h6 | 3.1126485 | 5.0203557 | 0.612888734 |
| Gm807 | 2.2368324 | 3.6113882 | 0.614509965 |
| Klra1 | 2.183711 | 3.5262547 | 0.614799165 |
| 2610206C17Rik | 2.2987483 | 3.733302 | 0.624058624 |
| Dgkk | 2.169744 | 3.533253 | 0.628419297 |
| 4833420D23Rik | 2.076229 | 3.3856995 | 0.630696566 |
| Rbm41 | 2.2752693 | 3.7699697 | 0.656933401 |
| Lipc | 2.1413317 | 3.5687315 | 0.666594437 |
| Crebbp | 2.1352015 | 3.5652878 | 0.669766437 |
| Pnlip | 2.1391582 | 3.575078 | 0.671254608 |
| Rasd1 | 2.1473644 | 3.6055665 | 0.679065975 |
| Cnga2 | 2.2006578 | 3.7017386 | 0.682105505 |
| 5033403F01Rik | 2.1610985 | 3.6601696 | 0.693661626 |
| Cd274 | 2.759736 | 4.6828246 | 0.696837886 |
| Fpr-rs6 | 2.1226175 | 3.607363 | 0.699488014 |
| Musk | 2.1924708 | 3.7418013 | 0.706659582 |
| Jph1 | 2.0947685 | 3.5918915 | 0.714696159 |
| 4931406P16Rik | 2.134399 | 3.6602776 | 0.71489848 |
| Yod1 | 2.8251972 | 4.855712 | 0.718716131 |
| A630081J09Rik | 2.174829 | 3.7505336 | 0.724518847 |
| Atp13a5 | 2.2313206 | 3.8511348 | 0.725944179 |
| Pkd2l2 | 2.9855072 | 5.177125 | 0.734085585 |
| Per3 | 2.6405284 | 4.614517 | 0.747573327 |
| Fancm | 2.8156056 | 4.9861736 | 0.770906266 |
| Ldb3 | 2.7837696 | 4.938508 | 0.774036185 |
| Popdc2 | 2.0623372 | 3.664906 | 0.777064391 |
| Acp1 | 2.305737 | 4.1414404 | 0.796146048 |
| Zfp446 | 2.1503046 | 3.864195 | 0.797045405 |
| Gm10561 | 2.435295 | 4.3977313 | 0.805831039 |
| Btbd7 | 2.8163714 | 5.090517 | 0.807473617 |
| Il17re | 2.1253014 | 3.8866494 | 0.828752101 |
| Spaca1 | 2.1858408 | 4.0082216 | 0.833720736 |
| Ece2 | 2.4211013 | 4.4415913 | 0.834533441 |
| B3gat2 | 2.177665 | 3.9971426 | 0.835517676 |
| Lyst | 2.1176054 | 3.900875 | 0.8421161 |
| Dopey1 | 2.0958662 | 3.8725512 | 0.847709172 |
| Sgk2 | 2.1575446 | 3.9873722 | 0.8481065 |
| Speer5-ps1 | 2.406009 | 4.4761963 | 0.860423756 |
| Lce3c | 2.2501645 | 4.2027893 | 0.867769801 |
| Gm10554 | 2.8593428 | 5.4223304 | 0.896355484 |
| Klra9 | 2.5865164 | 4.910617 | 0.898544699 |
| Gm12758 | 2.3732188 | 4.521149 | 0.905070447 |
| Nbea | 2.6196184 | 5.0152707 | 0.914504303 |
| Tcf4 | 2.2421043 | 4.3292556 | 0.930889477 |
| A230006K03Rik | 2.2355769 | 4.317862 | 0.931430764 |
| Kitl | 2.674532 | 5.166814 | 0.931857237 |
| Sox30 | 2.1573064 | 4.202837 | 0.948187332 |
| Itga1 | 2.1159585 | 4.128008 | 0.950892704 |
| Bach2 | 2.6730514 | 5.2419024 | 0.961018183 |
| Luc7l3 | 2.1742697 | 4.2802176 | 0.968577127 |
| Srsf12 | 2.1623847 | 4.2847414 | 0.981488955 |
| Cep72 | 2.0733292 | 4.1096573 | 0.982153775 |
| Gm9725 | 2.1391714 | 4.28313 | 1.002237876 |
| Mrpl47 | 2.315041 | 4.6474695 | 1.007510666 |
| Zfp85-rs1 | 2.093985 | 4.3714433 | 1.087619205 |
| Thoc1 | 2.3011732 | 4.817175 | 1.093356119 |
| 2410002O22Rik | 2.2063766 | 4.6554813 | 1.11001209 |
| Tspan13 | 2.25259 | 4.806278 | 1.133667467 |
| Fam19a5 | 2.0931377 | 4.4943395 | 1.147178134 |
| Pbrm1 | 2.2527454 | 4.866432 | 1.160222811 |
| Stxbp6 | 2.842564 | 6.172481 | 1.171448383 |
| Nbeal1 | 2.2009544 | 4.786094 | 1.174553912 |
| Tmprss11f | 2.2978761 | 5.05893 | 1.201567787 |
| Bicd1 | 2.0948758 | 4.618641 | 1.204732615 |
| Abhd15 | 2.224064 | 4.9440627 | 1.222985804 |
| Snca | 2.3715034 | 6.017893 | 1.537585651 |
| 5031434C07Rik | 2.3668678 | 6.2121415 | 1.624625465 |
| Fbxo30 | 2.230653 | 7.704474 | 2.453909685 |
| AU020094 | 2.2394578 | 7.9983425 | 2.571553123 |
| Prpmp5 | 2.2372317 | 8.317036 | 2.717556836 |

(D) Genes up-regulated over one fold in MSCs caused by the interaction of TCs and MSCs.

| GeneSymbol | MSCs cocultured TCs and stimulated with 1μg/ml LPS compared with MSCs | MSCs cocultured TCs compared with MSCs | MSCs stimulated with 1μg/ml LPS compared with MSCs |
| --- | --- | --- | --- |
| AU020094 | 2.571553123 | 2.55212543 | 2.64351005 |
| Fbxo30 | 2.453909685 | 2.222749123 | 2.289442598 |
| Prpmp5 | 2.717556836 | 2.798313782 | 2.851032059 |

(E) Genes down-regulated over 0.5 fold in MSCs caused by the interaction of TCs and MSCs.

| GeneSymbol | MSCs cocultured TCs and stimulated with 1μg/ml LPS compared with MSCs | MSCs cocultured TCs compared with MSCs | MSCs stimulated with 1μg/ml LPS compared with MSCs |
| --- | --- | --- | --- |
| 1700013N18Rik | -0.536887635 | -0.60373112 | -0.608380455 |
| 2410004P03Rik | -0.540343466 | -0.526710781 | -0.544693316 |
| 4930435E12Rik | -0.696296284 | -0.695285128 | -0.695587669 |
| 4930451I11Rik | -0.653933326 | -0.653214307 | -0.651120087 |
| Actrt2 | -0.514687189 | -0.518608489 | -0.511018247 |
| B4galnt3 | -0.516671752 | -0.513048938 | -0.516987893 |
| Cd164l2 | -0.52243896 | -0.51124247 | -0.510724823 |
| Ermap | -0.514728082 | -0.63616173 | -0.634390626 |
| Gfi1 | -0.525697596 | -0.524690524 | -0.522695159 |
| Gm10487 | -0.658735501 | -0.678942094 | -0.674368386 |
| Hrasls | -0.672988141 | -0.667329613 | -0.670556078 |
| Hrk | -0.53218936 | -0.561226333 | -0.572477217 |
| Igh-VJ558 | -0.595533797 | -0.585513013 | -0.597275289 |
| Krt26 | -0.578841941 | -0.564359114 | -0.582674078 |
| Mgam | -0.526262631 | -0.572690731 | -0.588845335 |
| Ncam2 | -0.531732244 | -0.533308832 | -0.521426597 |
| Olfr243 | -0.540494609 | -0.534285107 | -0.534700426 |
| Olfr366 | -0.581098186 | -0.561558379 | -0.567836686 |
| Patl2 | -0.537179718 | -0.534101589 | -0.534298442 |
| Rnase11 | -0.558544746 | -0.564855251 | -0.54962076 |
| Rsph10b2 | -0.59325083 | -0.587884435 | -0.591665159 |
| Sycp3 | -0.616112382 | -0.615240641 | -0.613173044 |
| Tlx3 | -0.608930759 | -0.612116382 | -0.610030695 |
| Vmn2r37 | -0.617862679 | -0.619702431 | -0.621490246 |
| Zfp874a | -0.52456607 | -0.528511864 | -0.518780861 |
