# Supplementary material for: Mechanisms of interactions between lung‐origin telocytes and mesenchymal stem cells to treat experimental acute lung injury
Source: Clin Transl Med. 2020 Dec 8;10(8):e231. doi: 10.1002/ctm2.231 (PMC7724099; doi:10.1002/ctm2.231)
Supplement: Supplementary file 4 — Supporting Table S2 [file CTM2-10-e231-s004.docx]

Supplement table 2：Alterations of gene expression profiles of TCs caused by the interaction of MSCs and TCs.

(A) Genes gene expression profiles in TCs stimulated with 1μg/ml LPS compared with TCs.

| GeneSymbol | TCs | TCs stimulated with 1μg/ml LPS | Fold Change |
| --- | --- | --- | --- |
| Dpep3 | 6.430064 | 2.073431 | -0.677541157 |
| Gm5463 | 7.044654 | 2.296011 | -0.674077535 |
| Cbln3 | 6.252815 | 2.0794046 | -0.667445047 |
| Lrrc39 | 6.210599 | 2.1079419 | -0.660589599 |
| 4930435F18Rik | 6.1014457 | 2.0870926 | -0.657934742 |
| AI464131 | 17.903744 | 6.226561 | -0.652220172 |
| LOC100505038 | 6.056363 | 2.1352596 | -0.647435334 |
| Raver2 | 5.758087 | 2.0610814 | -0.642054488 |
| Fam81b | 5.758379 | 2.080831 | -0.638642924 |
| Slco6d1 | 5.634415 | 2.065624 | -0.633391577 |
| Kcnd3 | 6.79689 | 2.4994912 | -0.632259578 |
| Vmn1r45 | 5.639136 | 2.0870533 | -0.629898392 |
| Muc6 | 5.6815057 | 2.1034737 | -0.629768267 |
| AI661453 | 5.575461 | 2.0810905 | -0.626741089 |
| Alox15 | 5.6285334 | 2.106031 | -0.625829528 |
| Gtdc1 | 5.7290435 | 2.156893 | -0.623516037 |
| Olfr1335 | 5.4793525 | 2.0684457 | -0.622501801 |
| 1700014D04Rik | 5.369945 | 2.0827262 | -0.612151298 |
| Cdx2 | 5.6121144 | 2.1978965 | -0.608365699 |
| St8sia6 | 5.2329755 | 2.065159 | -0.605356647 |
| Mbd3l1 | 5.334397 | 2.1245325 | -0.601729586 |
| D17Ertd648e | 5.336972 | 2.1414115 | -0.59875909 |
| 1110032F04Rik | 5.255561 | 2.1242414 | -0.595810723 |
| Rdh13 | 5.6265993 | 2.335013 | -0.585004569 |
| E130309F12Rik | 4.9763527 | 2.0687447 | -0.584284952 |
| Olfr45 | 5.137594 | 2.1369994 | -0.584046657 |
| Sowahb | 5.0441766 | 2.101557 | -0.583369662 |
| Olfr19 | 5.0832157 | 2.1202176 | -0.582898361 |
| A730089K16Rik | 5.1043677 | 2.1298056 | -0.582748398 |
| Vmn1r31 | 5.011687 | 2.1016574 | -0.580648712 |
| 4930447N08Rik | 5.277951 | 2.2458632 | -0.574481991 |
| Copg2as2 | 4.8978653 | 2.1177864 | -0.567610322 |
| Wdr86 | 6.059076 | 2.664195 | -0.560296818 |
| Heph | 4.8206644 | 2.1217072 | -0.559872452 |
| F8a | 6.936492 | 3.057147 | -0.559266125 |
| 4933402N03Rik | 5.8885784 | 2.6124067 | -0.556360377 |
| Hnf1a | 5.5805426 | 2.4878314 | -0.554195429 |
| Cela3b | 4.8282223 | 2.1622026 | -0.552174182 |
| Nlrp4a | 4.683074 | 2.0979683 | -0.552010432 |
| Olfr687 | 4.801122 | 2.1710296 | -0.547807867 |
| Klra22 | 5.0092196 | 2.2680275 | -0.547229373 |
| Vmn1r200 | 4.7452054 | 2.1912131 | -0.538225869 |
| AI314760 | 4.500931 | 2.0870357 | -0.536310221 |
| Dusp15 | 4.540866 | 2.1106305 | -0.535192076 |
| 6330409D20Rik | 4.514279 | 2.1160865 | -0.531245964 |
| Gm4319 | 5.3845706 | 2.542585 | -0.527801716 |
| Akap6 | 4.4553313 | 2.1044867 | -0.52764754 |
| 1700119I11Rik | 4.4879813 | 2.122018 | -0.52717762 |
| Olfr345 | 4.612158 | 2.188507 | -0.525491755 |
| AI465300 | 5.735914 | 2.7229214 | -0.525285526 |
| BC046401 | 4.5418205 | 2.161135 | -0.524169879 |
| Gm9325 | 7.303095 | 3.4848013 | -0.522832265 |
| Ntn5 | 4.505425 | 2.1620474 | -0.52012354 |
| Rdh1 | 5.29203 | 2.5553427 | -0.517133746 |
| Calhm1 | 4.38077 | 2.1449125 | -0.510380025 |
| Adamtsl3 | 4.5159636 | 2.2291467 | -0.506385149 |
| Gm5712 | 5.2287316 | 2.5978286 | -0.503162756 |
| Acsm3 | 5.4640894 | 2.7217734 | -0.501879783 |
| Gja6 | 6.24381 | 3.128211 | -0.49899004 |
| LOC100041223 | 4.17028 | 2.0959935 | -0.497397417 |
| Wnt8a | 4.149054 | 2.0868988 | -0.497018164 |
| Tctex1d1 | 5.452116 | 2.7439601 | -0.496716486 |
| Lcmt2 | 4.411225 | 2.2223134 | -0.496214 |
| Kpna7 | 4.259743 | 2.1479576 | -0.49575418 |
| Vmn1r168 | 4.2043247 | 2.120415 | -0.495658601 |
| Cpeb2 | 5.723275 | 2.8872945 | -0.495517077 |
| 2900011O08Rik | 4.199082 | 2.122591 | -0.494510705 |
| Pf4 | 4.234008 | 2.1442738 | -0.493559341 |
| Mageb5 | 4.088491 | 2.0712254 | -0.493401013 |
| Prpf31 | 4.1288223 | 2.0982835 | -0.491796123 |
| Gabra5 | 4.093771 | 2.0811152 | -0.491638589 |
| Cd55 | 4.247733 | 2.1643066 | -0.490479604 |
| Pot1a | 5.3715153 | 2.7616282 | -0.485875392 |
| Olfr975 | 6.465602 | 3.3248122 | -0.485769121 |
| Gm364 | 4.239618 | 2.1895728 | -0.483544791 |
| A430054B03 | 4.034481 | 2.0970755 | -0.480211829 |
| Mtap7d2 | 5.7907515 | 3.010098 | -0.480188711 |
| Olfr1462 | 4.118645 | 2.1464052 | -0.478856469 |
| Ppp1r17 | 4.3850474 | 2.3170052 | -0.471612279 |
| 2810404F17Rik | 3.9580534 | 2.093702 | -0.471027349 |
| Gm7849 | 3.9399326 | 2.0851395 | -0.470767723 |
| Mup5 | 4.085096 | 2.1653461 | -0.469939972 |
| B4galnt3 | 3.9283855 | 2.1033542 | -0.464575409 |
| Kirrel3 | 3.8426025 | 2.0648234 | -0.462649754 |
| Ccl25 | 4.181516 | 2.261198 | -0.459239663 |
| Dnahc10 | 3.9457304 | 2.139664 | -0.457726762 |
| Gpat2 | 6.1075644 | 3.34115 | -0.452948871 |
| Bcl2a1d | 3.8340144 | 2.1129584 | -0.448891376 |
| Alox8 | 3.9176161 | 2.1726208 | -0.445422741 |
| LOC100041550 | 3.876818 | 2.1606548 | -0.442673141 |
| Syt14 | 5.5834956 | 3.1419764 | -0.437274313 |
| Vat1l | 3.8028202 | 2.1627188 | -0.43128555 |
| Pcdhb4 | 3.8626637 | 2.1997027 | -0.430521818 |
| Gm10579 | 3.6035252 | 2.060199 | -0.428282339 |
| Ern1 | 3.7139072 | 2.1234076 | -0.428255073 |
| Npsr1 | 6.4140406 | 3.6755855 | -0.426946954 |
| Entpd3 | 3.8591979 | 2.2209384 | -0.424507771 |
| Gm2670 | 3.987846 | 2.298503 | -0.423622928 |
| Fgf8 | 3.592833 | 2.0720882 | -0.423271775 |
| Havcr2 | 5.7137804 | 3.2956798 | -0.423205029 |
| Nlrp4e | 3.636526 | 2.099507 | -0.422661353 |
| Reg4 | 3.6096725 | 2.0939436 | -0.419907595 |
| C030017G13Rik | 6.2505717 | 3.6262884 | -0.419846924 |
| Srcin1 | 3.657994 | 2.1244254 | -0.419237593 |
| Tas2r117 | 5.3297343 | 3.0981176 | -0.418710685 |
| Zfp853 | 3.8712857 | 2.2577825 | -0.416787425 |
| BC048671 | 5.086596 | 2.9680948 | -0.416487018 |
| Ccdc8 | 4.139486 | 2.4234946 | -0.414542144 |
| Jag2 | 3.5771368 | 2.0952008 | -0.414279935 |
| Fut4 | 3.5308366 | 2.0741155 | -0.412571089 |
| 4930528H21Rik | 3.632256 | 2.136243 | -0.411868822 |
| Olfr146 | 3.5766542 | 2.10556 | -0.411304565 |
| Gm1043 | 3.6312258 | 2.1407986 | -0.410447403 |
| Olfr804 | 3.6781287 | 2.1753411 | -0.408573958 |
| Serpinb3c | 3.545411 | 2.097669 | -0.408342502 |
| Vmn2r98 | 6.6983566 | 3.9691215 | -0.407448463 |
| Rpl10l | 3.695322 | 2.20029 | -0.404574216 |
| Ints6 | 3.6041982 | 2.1492817 | -0.403672723 |
| Olfr181 | 3.5244634 | 2.102063 | -0.40357928 |
| Ticam2 | 3.8010352 | 2.2725117 | -0.402133477 |
| Hand1 | 4.2849674 | 2.5646942 | -0.401467045 |
| Rbm3 | 3.5063045 | 2.1031067 | -0.400192796 |
| 4930426I24Rik | 3.7310517 | 2.2384162 | -0.400057576 |
| Olfr503 | 4.406626 | 2.6571345 | -0.397013838 |
| Ahsg | 3.6679893 | 2.2153804 | -0.396023211 |
| Wdr65 | 3.6971414 | 2.2367659 | -0.395001257 |
| Prrt2 | 5.758792 | 3.484582 | -0.394910947 |
| Glyctk | 4.5019445 | 2.7288024 | -0.393861386 |
| Timm8a2 | 3.5041666 | 2.1241972 | -0.39380816 |
| Gm10802 | 3.6274495 | 2.2029152 | -0.392709616 |
| H2-M9 | 3.7653387 | 2.287809 | -0.392402867 |
| Dnajc6 | 3.460143 | 2.1048281 | -0.39169332 |
| Cd44 | 3.8335366 | 2.3339567 | -0.391174014 |
| A330023F24Rik | 3.8923447 | 2.3711636 | -0.390813563 |
| Gm534 | 3.4822392 | 2.1240249 | -0.390040495 |
| Bmp10 | 3.4150665 | 2.0840843 | -0.389738296 |
| Olfr448 | 3.9480047 | 2.4198053 | -0.387081454 |
| Chrnb2 | 3.413835 | 2.0962596 | -0.385951694 |
| 0610012H03Rik | 3.3498535 | 2.077603 | -0.379792878 |
| Gm11783 | 3.833106 | 2.384232 | -0.377989547 |
| Olfr130 | 3.383409 | 2.1153092 | -0.37479944 |
| Zfp185 | 3.523439 | 2.2068264 | -0.373672597 |
| 4932416K20Rik | 3.4134593 | 2.140631 | -0.372885155 |
| Lrp8 | 4.42931 | 2.7782612 | -0.372755305 |
| Elf5 | 3.3910384 | 2.1288629 | -0.372209144 |
| Cyp2c38 | 3.423481 | 2.1500497 | -0.371969729 |
| Car3 | 3.3409548 | 2.1196394 | -0.365558792 |
| Cyb5rl | 5.7440596 | 3.65624 | -0.363474571 |
| A330033J07Rik | 3.2436934 | 2.0673935 | -0.362642135 |
| Gm13547 | 4.073487 | 2.5965972 | -0.36256156 |
| Olfr1161 | 3.9789093 | 2.5384545 | -0.362022527 |
| C030034L19Rik | 3.3303394 | 2.1295602 | -0.360557606 |
| Olfr39 | 6.4883547 | 4.159631 | -0.358908199 |
| Btnl1 | 3.3026605 | 2.1182454 | -0.358624539 |
| Tlr3 | 3.4314172 | 2.2010858 | -0.358549057 |
| Rabep1 | 3.8080394 | 2.44438 | -0.358100129 |
| 1190003K10Rik | 3.4031928 | 2.1848054 | -0.35801304 |
| C8a | 3.2798629 | 2.1069155 | -0.357620863 |
| Tmprss7 | 3.4761868 | 2.234547 | -0.35718443 |
| 2210408I21Rik | 3.5039127 | 2.2544754 | -0.356583456 |
| Bpifb2 | 3.1993878 | 2.0655112 | -0.354404239 |
| Olfr140 | 3.2421663 | 2.098227 | -0.352831778 |
| Kcna2 | 3.269738 | 2.1212718 | -0.351241047 |
| Ctnna2 | 3.5914855 | 2.3362527 | -0.349502399 |
| Pdia2 | 3.2647057 | 2.1372757 | -0.345338938 |
| AI854517 | 3.2302816 | 2.1163354 | -0.34484492 |
| St6galnac5 | 3.1937962 | 2.0930822 | -0.344641277 |
| 4930554N03Rik | 3.2030501 | 2.1070151 | -0.342184782 |
| Vmn1r56 | 3.3089879 | 2.196593 | -0.336173759 |
| Clcn6 | 7.261939 | 4.822648 | -0.335900784 |
| Hus1b | 3.918069 | 2.60854 | -0.334228162 |
| Gm12052 | 2.413618 | 3.6255264 | 0.502112762 |
| Gm6117 | 2.156596 | 3.2397027 | 0.502229764 |
| Olfr827 | 2.0888608 | 3.1389592 | 0.502713441 |
| Gm44 | 2.9211938 | 4.407498 | 0.508800272 |
| Pou2af1 | 2.7522168 | 4.160872 | 0.511825667 |
| Olfr1322 | 2.3054862 | 3.4907804 | 0.514118974 |
| LOC100044193 | 2.2830126 | 3.465331 | 0.517876423 |
| Galnt13 | 3.7099242 | 5.6343555 | 0.518725234 |
| Il7 | 2.2153368 | 3.3668642 | 0.519797893 |
| 2310003L06Rik | 2.2881734 | 3.4794796 | 0.520636329 |
| Zfp369 | 2.6492 | 4.0777636 | 0.539243394 |
| Spon1 | 2.1975265 | 3.3912902 | 0.543230628 |
| Zic4 | 2.4023445 | 3.7079537 | 0.543472928 |
| Tnfaip8l3 | 2.2801516 | 3.5446818 | 0.554581634 |
| Fam47e | 2.248822 | 3.5040183 | 0.558157249 |
| Prrt3 | 2.1062489 | 3.2987518 | 0.566173779 |
| Kcnq1ot1 | 2.2085295 | 3.47785 | 0.574735588 |
| Gm14124 | 2.232318 | 3.5179734 | 0.57592843 |
| Gpr26 | 3.6293297 | 5.7284493 | 0.578376663 |
| Gm7480 | 2.2149203 | 3.5030656 | 0.581576366 |
| Rbm41 | 2.8973792 | 4.5853558 | 0.582587395 |
| Zfp677 | 3.1494436 | 4.9918237 | 0.5849859 |
| Sytl3 | 2.3736968 | 3.7720163 | 0.589089348 |
| 1700011E24Rik | 2.1424935 | 3.4148862 | 0.593884042 |
| Olfr592 | 2.095842 | 3.346179 | 0.596579799 |
| Gm12522 | 3.528911 | 5.634415 | 0.596644121 |
| Rp1l1 | 2.0778227 | 3.3222563 | 0.598912313 |
| Ankrd33b | 2.1089997 | 3.388122 | 0.60650663 |
| D630028G08Rik | 2.2038186 | 3.5406466 | 0.606596205 |
| Dmd | 2.3603277 | 3.7977738 | 0.609002767 |
| Zscan4c | 2.0820904 | 3.3505883 | 0.609242471 |
| LOC100862611 | 2.527308 | 4.0676336 | 0.609472846 |
| Olfr1065 | 2.16364 | 3.488351 | 0.612260358 |
| Gm10002 | 2.4146678 | 3.8998015 | 0.615046799 |
| Ano5 | 2.1130176 | 3.4638007 | 0.639267321 |
| Olfr629 | 2.1171026 | 3.4743938 | 0.641107899 |
| Lcorl | 2.1639042 | 3.5606916 | 0.645494103 |
| Mei4 | 2.1734483 | 3.5918753 | 0.652615938 |
| B830008H07Rik | 2.3014624 | 3.806474 | 0.653937079 |
| B230307C23Rik | 2.0931494 | 3.4739785 | 0.6596897 |
| Mis18bp1 | 2.7923234 | 4.694275 | 0.68113586 |
| Uba6 | 2.5082812 | 4.2479334 | 0.693563465 |
| Gm11826 | 2.2815976 | 3.9454257 | 0.729238188 |
| Adora3 | 2.1499732 | 3.7216964 | 0.731043159 |
| Ccdc48 | 2.421556 | 4.1939483 | 0.731922904 |
| Mlc1 | 2.1946793 | 3.8136601 | 0.737684453 |
| Olfr433 | 2.4939818 | 4.3434186 | 0.741559862 |
| Vps13b | 2.2548957 | 3.9369457 | 0.74595468 |
| Lrrc48 | 2.219398 | 3.878977 | 0.747760879 |
| Padi6 | 2.1045527 | 3.7349322 | 0.774691696 |
| Gm6712 | 2.4253793 | 4.3088193 | 0.776554826 |
| Samd12 | 2.3432486 | 4.16611 | 0.777920618 |
| Ly9 | 2.3078728 | 4.116399 | 0.783633396 |
| Efcab3 | 2.4088285 | 4.455032 | 0.849460018 |
| Olfr453 | 2.3424168 | 4.333922 | 0.850192502 |
| C79562 | 2.2019753 | 4.092624 | 0.858614854 |
| Kcnh7 | 2.4951067 | 4.639031 | 0.85925155 |
| Gm9926 | 2.3376193 | 4.4388614 | 0.898881225 |
| Nfasc | 2.6960902 | 5.1243167 | 0.900647352 |
| Ctcfl | 2.134423 | 4.07034 | 0.906997816 |
| Olfr476 | 2.2050493 | 4.207048 | 0.907915619 |
| 1700009J07Rik | 2.1259675 | 4.085047 | 0.921500211 |
| Pdcd1 | 2.1960406 | 4.232701 | 0.927423837 |
| BB287469 | 2.1526918 | 4.1757293 | 0.939771081 |
| Ccdc50 | 2.180384 | 4.231349 | 0.940643942 |
| 4930529I22Rik | 2.085304 | 4.048613 | 0.941497738 |
| Olfr1431 | 2.1081872 | 4.11242 | 0.950690147 |
| Gm10554 | 2.6059313 | 5.14412 | 0.974004457 |
| Olfr767 | 2.2505574 | 4.4771557 | 0.989354148 |
| Phip | 2.0911555 | 4.210945 | 1.013692908 |
| Rfx4 | 2.2704115 | 4.70218 | 1.071069496 |
| Olfr113 | 2.1112175 | 4.406697 | 1.087277602 |
| Txlng | 2.196222 | 4.748718 | 1.162221305 |

(B) Genes gene expression profiles in TCs cocultured MSCs compared with TCs.

| GeneSymbol | TCs | TCs cocultured MSCs | Fold Change |
| --- | --- | --- | --- |
| AI464131 | 17.903744 | 5.101181 | -0.715077416 |
| Dpep3 | 6.430064 | 2.103184 | -0.672913987 |
| Gm5463 | 7.044654 | 2.329484 | -0.669325988 |
| C030017G13Rik | 6.2505717 | 2.1145124 | -0.66170896 |
| Lrrc39 | 6.210599 | 2.134728 | -0.656276633 |
| 4930435F18Rik | 6.1014457 | 2.1147897 | -0.653395309 |
| 4933402N03Rik | 5.8885784 | 2.1001582 | -0.643350558 |
| Gja6 | 6.24381 | 2.2352767 | -0.642001166 |
| Raver2 | 5.758087 | 2.0783644 | -0.63905297 |
| LOC100505038 | 6.056363 | 2.2040145 | -0.636082827 |
| Muc6 | 5.6815057 | 2.0861125 | -0.632824006 |
| Slco6d1 | 5.634415 | 2.0741725 | -0.631874383 |
| Cpeb2 | 5.723275 | 2.111124 | -0.631133573 |
| Vmn1r45 | 5.639136 | 2.1133249 | -0.625239593 |
| Olfr1335 | 5.4793525 | 2.0672288 | -0.622723889 |
| AI661453 | 5.575461 | 2.1052058 | -0.622415833 |
| Alox15 | 5.6285334 | 2.1347773 | -0.620722283 |
| Cdx2 | 5.6121144 | 2.1492975 | -0.617025359 |
| Npsr1 | 6.4140406 | 2.4803927 | -0.613287028 |
| Hnf1a | 5.5805426 | 2.1622186 | -0.612543304 |
| Gtdc1 | 5.7290435 | 2.236992 | -0.60953482 |
| 1700014D04Rik | 5.369945 | 2.113459 | -0.606428185 |
| Prrt2 | 5.758792 | 2.2713766 | -0.605581066 |
| D17Ertd648e | 5.336972 | 2.1199877 | -0.602773314 |
| St8sia6 | 5.2329755 | 2.0924838 | -0.60013499 |
| Wdr86 | 6.059076 | 2.4284194 | -0.599209615 |
| Pot1a | 5.3715153 | 2.216801 | -0.58730435 |
| E130309F12Rik | 4.9763527 | 2.06688 | -0.584659664 |
| Vmn1r31 | 5.011687 | 2.118141 | -0.577359679 |
| 1110032F04Rik | 5.255561 | 2.2371502 | -0.574327041 |
| A730089K16Rik | 5.1043677 | 2.1817558 | -0.5725708 |
| Rdh1 | 5.29203 | 2.2911546 | -0.567055629 |
| Tas2r117 | 5.3297343 | 2.33016 | -0.562799969 |
| Copg2as2 | 4.8978653 | 2.149728 | -0.561088787 |
| Heph | 4.8206644 | 2.1469693 | -0.554632075 |
| Kcnd3 | 6.79689 | 3.068867 | -0.54848953 |
| Nlrp4a | 4.683074 | 2.1297407 | -0.545225914 |
| Olfr45 | 5.137594 | 2.3410861 | -0.544322479 |
| Olfr345 | 4.612158 | 2.1148224 | -0.54146792 |
| Olfr687 | 4.801122 | 2.2162805 | -0.538382799 |
| Dusp15 | 4.540866 | 2.103603 | -0.536739688 |
| Cela3b | 4.8282223 | 2.2541485 | -0.533130755 |
| Olfr503 | 4.406626 | 2.0600476 | -0.532511359 |
| AI314760 | 4.500931 | 2.113438 | -0.530444257 |
| Akap6 | 4.4553313 | 2.1359365 | -0.520588626 |
| Vmn1r200 | 4.7452054 | 2.286643 | -0.518115064 |
| Gm9325 | 7.303095 | 3.5588527 | -0.512692537 |
| Acsm3 | 5.4640894 | 2.6922417 | -0.507284471 |
| 4930447N08Rik | 5.277951 | 2.606646 | -0.506125388 |
| Cbln3 | 6.252815 | 3.092552 | -0.505414441 |
| Klra22 | 5.0092196 | 2.4914117 | -0.502634762 |
| F8a | 6.936492 | 3.4513772 | -0.502431892 |
| Ntn5 | 4.505425 | 2.245013 | -0.50170894 |
| Tctex1d1 | 5.452116 | 2.720589 | -0.501003097 |
| BC046401 | 4.5418205 | 2.2687547 | -0.5004746 |
| BC048671 | 5.086596 | 2.5425556 | -0.500145952 |
| LOC100041223 | 4.17028 | 2.0981357 | -0.496883734 |
| Ccl25 | 4.181516 | 2.1117368 | -0.494982968 |
| Wnt8a | 4.149054 | 2.1150188 | -0.490240715 |
| 2900011O08Rik | 4.199082 | 2.1411555 | -0.49008962 |
| Vmn1r168 | 4.2043247 | 2.1520061 | -0.488144648 |
| Olfr19 | 5.0832157 | 2.6054676 | -0.487437136 |
| Glyctk | 4.4697533 | 2.2918868 | -0.487245348 |
| Gabra5 | 4.093771 | 2.1055377 | -0.485672819 |
| Kpna7 | 4.259743 | 2.194375 | -0.48485742 |
| A430054B03 | 4.034481 | 2.0904922 | -0.481843588 |
| Ccdc8 | 4.139486 | 2.1460886 | -0.481556744 |
| Pf4 | 4.234008 | 2.1964834 | -0.48122833 |
| Cd55 | 4.247733 | 2.213821 | -0.478822939 |
| 1700119I11Rik | 4.4879813 | 2.3507607 | -0.476209783 |
| Gm13547 | 4.073487 | 2.1463895 | -0.473083012 |
| Olfr1462 | 4.118645 | 2.1923566 | -0.467699547 |
| 2810404F17Rik | 3.9580534 | 2.1090343 | -0.467153652 |
| A330023F24Rik | 3.8923447 | 2.0759387 | -0.466661136 |
| B4galnt3 | 3.9283855 | 2.101051 | -0.465161706 |
| Gm7849 | 3.9399326 | 2.1150005 | -0.46318866 |
| Hand1 | 4.2849674 | 2.3174462 | -0.459168301 |
| Mageb5 | 4.088491 | 2.222599 | -0.456376693 |
| Kirrel3 | 3.8426025 | 2.0896554 | -0.456187467 |
| Dnahc10 | 3.9457304 | 2.1503196 | -0.455026223 |
| Olfr448 | 3.9480047 | 2.1543202 | -0.45432684 |
| Lcmt2 | 4.411225 | 2.4124823 | -0.453103775 |
| Gm364 | 4.239618 | 2.3265054 | -0.451246457 |
| Adamtsl3 | 4.5159636 | 2.4905076 | -0.448510258 |
| Mup5 | 4.085096 | 2.2556505 | -0.447834151 |
| Rabep1 | 3.8080394 | 2.1215234 | -0.442883023 |
| Bcl2a1d | 3.8340144 | 2.1376443 | -0.44245272 |
| Gm4319 | 5.3845706 | 3.0213866 | -0.438880679 |
| Gm2670 | 3.987846 | 2.2377656 | -0.438853557 |
| Srcin1 | 3.657994 | 2.066315 | -0.435123458 |
| Olfr39 | 6.4883547 | 3.6671245 | -0.434814422 |
| Vmn2r98 | 6.6983566 | 3.8161466 | -0.430286139 |
| Entpd3 | 3.8591979 | 2.206973 | -0.428126503 |
| Nlrp4e | 3.636526 | 2.087011 | -0.4260976 |
| Vat1l | 3.8028202 | 2.1872897 | -0.424824319 |
| Gm10579 | 3.6035252 | 2.0788307 | -0.423111929 |
| Rpl10l | 3.695322 | 2.1455815 | -0.419379015 |
| Ern1 | 3.7139072 | 2.1596363 | -0.418500198 |
| Olfr804 | 3.6781287 | 2.1526468 | -0.414744025 |
| LOC100041550 | 3.876818 | 2.270951 | -0.414222953 |
| Zfp853 | 3.8712857 | 2.2714071 | -0.413268026 |
| Alox8 | 3.9176161 | 2.301181 | -0.412606815 |
| Jag2 | 3.5771368 | 2.1035166 | -0.411955226 |
| H2-M9 | 3.7653387 | 2.2144635 | -0.411881991 |
| Olfr146 | 3.5766542 | 2.1151025 | -0.408636569 |
| Gpr26 | 3.6293297 | 2.149005 | -0.407878265 |
| Havcr2 | 5.7137804 | 3.3859289 | -0.40741004 |
| Clcn6 | 7.261939 | 4.306494 | -0.406977393 |
| Lrp8 | 4.42931 | 2.6285071 | -0.406565108 |
| Gpat2 | 6.1075644 | 3.6257138 | -0.406356845 |
| Zfp185 | 3.523439 | 2.0931966 | -0.405922282 |
| Olfr1161 | 3.9789093 | 2.3641908 | -0.405819379 |
| Serpinb3c | 3.545411 | 2.107777 | -0.405491493 |
| 4930528H21Rik | 3.632256 | 2.1608982 | -0.405080974 |
| Fut4 | 3.5308366 | 2.103878 | -0.404141783 |
| Syt14 | 5.5834956 | 3.3283293 | -0.403898644 |
| Gm10802 | 3.6274495 | 2.1635041 | -0.403574302 |
| Mtap7d2 | 5.7907515 | 3.4747918 | -0.39994113 |
| Timm8a2 | 3.5041666 | 2.104914 | -0.399311094 |
| Gm12522 | 3.528911 | 2.1220274 | -0.398673585 |
| Rbm3 | 3.5063045 | 2.112359 | -0.397554034 |
| Dnajc6 | 3.460143 | 2.0874276 | -0.396722159 |
| Olfr181 | 3.5244634 | 2.1285994 | -0.396050077 |
| 4930426I24Rik | 3.7310517 | 2.2573764 | -0.394975846 |
| Ctnna2 | 3.5914855 | 2.177491 | -0.393707423 |
| Ints6 | 3.6041982 | 2.1862733 | -0.393409247 |
| Zscan4c | 3.4715815 | 2.1073778 | -0.392963178 |
| Ahsg | 3.6679893 | 2.2352161 | -0.390615425 |
| Mbd3l1 | 5.334397 | 3.2521038 | -0.390352124 |
| Ticam2 | 3.8010352 | 2.3174715 | -0.390305173 |
| Pcdhb4 | 3.8626637 | 2.3667266 | -0.387281217 |
| Chrnb2 | 3.413835 | 2.0957294 | -0.386107003 |
| 0610012H03Rik | 3.3498535 | 2.0639782 | -0.38386016 |
| Bmp10 | 3.4150665 | 2.1125705 | -0.381396965 |
| Tlr3 | 3.4314172 | 2.1253362 | -0.380624367 |
| Gm534 | 3.4822392 | 2.1606476 | -0.379523497 |
| Sowahb | 5.0441766 | 3.1376185 | -0.377972115 |
| Olfr130 | 3.383409 | 2.120393 | -0.373296873 |
| AI465300 | 5.735914 | 3.6181464 | -0.369211881 |
| Gm5712 | 5.2287316 | 3.2996204 | -0.368944392 |
| Elf5 | 3.3910384 | 2.145411 | -0.367329193 |
| Pdia2 | 3.2647057 | 2.0732832 | -0.364940246 |
| Gm11783 | 3.833106 | 2.435704 | -0.364561272 |
| 4932416K20Rik | 3.4134593 | 2.1716456 | -0.363799182 |
| Cyp2c38 | 3.423481 | 2.1782966 | -0.3637188 |
| Cyb5rl | 5.7440596 | 3.657994 | -0.363169212 |
| Car3 | 3.3409548 | 2.127996 | -0.363057531 |
| C030034L19Rik | 3.3303394 | 2.1226492 | -0.36263277 |
| 1190003K10Rik | 3.4031928 | 2.1727638 | -0.361551364 |
| A330033J07Rik | 3.2436934 | 2.0720375 | -0.361210434 |
| Vmn1r56 | 3.3089879 | 2.1270418 | -0.357192633 |
| 6330409D20Rik | 4.514279 | 2.914085 | -0.354473882 |
| Btnl1 | 3.3026605 | 2.1400387 | -0.352025829 |
| Olfr140 | 3.2421663 | 2.1017413 | -0.351747842 |
| Kcna2 | 3.269738 | 2.1221015 | -0.350987296 |
| Bpifb2 | 3.1993878 | 2.0787337 | -0.350271418 |
| Prpf31 | 4.1288223 | 2.6831353 | -0.350145125 |
| Ppp1r17 | 4.3850474 | 2.8500254 | -0.350058246 |
| C8a | 3.2798629 | 2.1364198 | -0.348625273 |
| Fam81b | 5.758379 | 3.7590132 | -0.347209831 |
| Gm1043 | 3.6312258 | 2.3705888 | -0.347165687 |
| AI854517 | 3.2302816 | 2.1090827 | -0.347090142 |
| Ankrd33b | 4.4031916 | 2.8787441 | -0.34621421 |
| Cd44 | 3.4286914 | 2.252079 | -0.343166609 |
| Tmprss7 | 3.4761868 | 2.2870493 | -0.342081012 |
| St6galnac5 | 3.1937962 | 2.1071048 | -0.340250702 |
| Calhm1 | 4.38077 | 2.899735 | -0.338076411 |
| Wdr65 | 3.6971414 | 2.450027 | -0.337318556 |
| Reg4 | 3.6096725 | 2.394345 | -0.336686361 |
| Hus1b | 3.918069 | 2.6005619 | -0.336264395 |
| 4930554N03Rik | 3.2030501 | 2.1277275 | -0.33571832 |
| Rdh13 | 5.6265993 | 3.742704 | -0.334819524 |
| Galnt13 | 3.7099242 | 5.5674467 | 0.500690149 |
| Pdcd1 | 2.1960406 | 3.3036706 | 0.504375921 |
| Vps13b | 2.2548957 | 3.3994753 | 0.50759758 |
| Olfr433 | 2.4939818 | 3.7979355 | 0.522840103 |
| Adora3 | 2.1499732 | 3.278702 | 0.524996684 |
| Olfr1322 | 2.3054862 | 3.539699 | 0.535337318 |
| Uba6 | 2.5082812 | 3.868495 | 0.542289198 |
| 2210408I21Rik | 2.1102555 | 3.2550774 | 0.542503929 |
| B230307C23Rik | 2.0931494 | 3.2450683 | 0.550328085 |
| Olfr975 | 2.4931657 | 3.8682275 | 0.551532455 |
| LOC100044193 | 2.2830126 | 3.5666234 | 0.562244291 |
| Gm12052 | 2.413618 | 3.7734847 | 0.563414219 |
| Mei4 | 2.3319387 | 3.6696348 | 0.573641194 |
| Tnfaip8l3 | 2.2801516 | 3.5924125 | 0.57551476 |
| Gm44 | 2.9211938 | 4.6110325 | 0.578475382 |
| Olfr476 | 2.2050493 | 3.5102403 | 0.591910122 |
| 1700011E24Rik | 2.1424935 | 3.4522908 | 0.611342485 |
| Rp1l1 | 2.0778227 | 3.350943 | 0.612718448 |
| LOC100862611 | 2.527308 | 4.1042953 | 0.623979072 |
| Gm6117 | 2.156596 | 3.5442133 | 0.643429414 |
| Gm10002 | 2.4146678 | 3.9764848 | 0.646804086 |
| Fam47e | 2.248822 | 3.7183352 | 0.6534591 |
| Olfr827 | 2.0888608 | 3.463592 | 0.65812485 |
| Zfp677 | 3.1494436 | 5.2230244 | 0.658395915 |
| Sytl3 | 2.3736968 | 3.9511662 | 0.664562298 |
| Dmd | 3.4376242 | 5.72861 | 0.666444517 |
| Padi6 | 2.1045527 | 3.5166204 | 0.670958584 |
| Lcorl | 2.1639042 | 3.6258123 | 0.67558818 |
| Ccdc48 | 2.421556 | 4.064628 | 0.678519101 |
| Il7 | 2.2153368 | 3.741179 | 0.688763081 |
| Gm14124 | 2.232318 | 3.7700686 | 0.688858218 |
| 2310003L06Rik | 2.2881734 | 3.8765821 | 0.694181962 |
| Olfr1065 | 2.16364 | 3.694055 | 0.707333475 |
| D630028G08Rik | 2.2038186 | 3.7671394 | 0.709369092 |
| Kcnh7 | 2.4951067 | 4.269746 | 0.71124786 |
| C79562 | 2.2019753 | 3.779001 | 0.716186826 |
| 1700009J07Rik | 2.1259675 | 3.6552732 | 0.719345757 |
| Prrt3 | 2.1062489 | 3.63676 | 0.726652534 |
| Samd12 | 2.3432486 | 4.074248 | 0.738717778 |
| Kcnq1ot1 | 2.2085295 | 3.8560624 | 0.745986368 |
| Pou2af1 | 2.7522168 | 4.834169 | 0.756463735 |
| Efcab3 | 2.4088285 | 4.247516 | 0.763311917 |
| Zic4 | 2.4023445 | 4.262159 | 0.774166444 |
| Gm10554 | 2.6059313 | 4.6335254 | 0.7780689 |
| Gm9926 | 2.3376193 | 4.1891994 | 0.792079403 |
| Ccdc50 | 2.180384 | 3.9191494 | 0.797458338 |
| Ly9 | 2.3078728 | 4.1763144 | 0.809594706 |
| BB287469 | 2.1526918 | 3.9241676 | 0.822911947 |
| Mis18bp1 | 2.7923234 | 5.1587267 | 0.847467489 |
| Gm7480 | 2.2149203 | 4.176904 | 0.885803295 |
| B830008H07Rik | 2.3014624 | 4.3662214 | 0.897150872 |
| Zfp369 | 2.6492 | 5.0375714 | 0.901544391 |
| Rbm41 | 2.8973792 | 5.5441327 | 0.913499172 |
| Gm6712 | 2.4253793 | 4.645027 | 0.915175494 |
| Ctcfl | 2.134423 | 4.095786 | 0.918919539 |
| Nfasc | 2.6960902 | 5.228109 | 0.939144692 |
| Mlc1 | 2.1946793 | 4.2986026 | 0.958647261 |
| Olfr767 | 2.2505574 | 4.410412 | 0.959697629 |
| Phip | 2.0911555 | 4.09881 | 0.960069445 |
| Olfr592 | 2.095842 | 4.1280575 | 0.969641557 |
| Lrrc48 | 2.219398 | 4.374101 | 0.970850204 |
| Olfr629 | 2.1171026 | 4.1939964 | 0.981007628 |
| Spon1 | 2.1975265 | 4.4698315 | 1.034028486 |
| Ano5 | 2.1130176 | 4.303104 | 1.036473336 |
| Olfr453 | 2.3424168 | 4.807699 | 1.052452407 |
| 4930529I22Rik | 2.085304 | 4.317768 | 1.070570046 |
| Txlng | 2.196222 | 4.5898876 | 1.089901476 |
| Olfr1431 | 2.1081872 | 4.538345 | 1.152723914 |
| Rfx4 | 2.1223037 | 4.806736 | 1.264867182 |
| Fgf8 | 2.2945633 | 5.366778 | 1.338910415 |
| Gm11826 | 2.2815976 | 5.354693 | 1.346905081 |
| Olfr113 | 2.1112175 | 4.9884963 | 1.362852856 |

(C) Genes gene expression profiles in TCs cocultured with MSCs and stimulated with 1μg/ml LPS compared with TCs.

| GeneSymbol | TCs | TCs cocultured MSCs and stimulated with 1μg/ml LPS | Fold Change |
| --- | --- | --- | --- |
| AI464131 | 17.903744 | 5.102025 | -0.715030275 |
| Dpep3 | 6.430064 | 2.0846224 | -0.675800676 |
| Npsr1 | 6.4140406 | 2.1398711 | -0.666377057 |
| 4930435F18Rik | 6.1014457 | 2.095812 | -0.65650567 |
| Gm5463 | 7.044654 | 2.428283 | -0.655301311 |
| Gja6 | 6.24381 | 2.1743624 | -0.651757116 |
| LOC100505038 | 6.056363 | 2.1486497 | -0.645224419 |
| 4933402N03Rik | 5.8885784 | 2.093325 | -0.644510974 |
| Raver2 | 5.758087 | 2.067606 | -0.640921369 |
| Cpeb2 | 5.723275 | 2.0942106 | -0.634088769 |
| Slco6d1 | 5.634415 | 2.064358 | -0.633616267 |
| Wdr86 | 6.059076 | 2.2337766 | -0.631333788 |
| Prrt2 | 5.758792 | 2.164294 | -0.624175695 |
| Alox15 | 5.6285334 | 2.1170263 | -0.623876035 |
| Olfr1335 | 5.4793525 | 2.07593 | -0.621135892 |
| Syt14 | 5.5834956 | 2.131101 | -0.618321361 |
| Gm4319 | 5.3845706 | 2.07182 | -0.615230228 |
| Cdx2 | 5.6121144 | 2.2109485 | -0.606040016 |
| Pot1a | 5.3715153 | 2.126856 | -0.604049159 |
| St8sia6 | 5.2329755 | 2.0748575 | -0.603503303 |
| 1110032F04Rik | 5.255561 | 2.1169417 | -0.597199671 |
| D17Ertd648e | 5.336972 | 2.1700277 | -0.593397211 |
| Gm5712 | 5.2287316 | 2.1642318 | -0.586088565 |
| Olfr45 | 5.137594 | 2.1324975 | -0.584922923 |
| A730089K16Rik | 5.1043677 | 2.127637 | -0.58317325 |
| E130309F12Rik | 4.9763527 | 2.0749547 | -0.58303705 |
| Olfr19 | 5.0832157 | 2.130766 | -0.580823218 |
| Rdh1 | 5.29203 | 2.223393 | -0.579860091 |
| Vmn1r31 | 5.011687 | 2.107154 | -0.579551955 |
| Muc6 | 5.6815057 | 2.4194105 | -0.574160332 |
| Copg2as2 | 4.8978653 | 2.1137748 | -0.568429373 |
| 4930447N08Rik | 5.277951 | 2.2787461 | -0.568251751 |
| Vmn1r45 | 5.639136 | 2.442278 | -0.566905639 |
| Heph | 4.8206644 | 2.1318338 | -0.557771788 |
| Klra22 | 5.0092196 | 2.2468417 | -0.551458734 |
| Nlrp4a | 4.683074 | 2.112655 | -0.548874308 |
| Olfr687 | 4.801122 | 2.2017527 | -0.541408717 |
| Lrrc39 | 6.210599 | 2.8604345 | -0.539426954 |
| Dusp15 | 4.540866 | 2.1012943 | -0.537248115 |
| Hnf1a | 5.5805426 | 2.588338 | -0.536185245 |
| AI314760 | 4.500931 | 2.0935524 | -0.534862365 |
| Vmn1r200 | 4.7452054 | 2.2228003 | -0.531569213 |
| AI661453 | 5.575461 | 2.625997 | -0.529008095 |
| 6330409D20Rik | 4.514279 | 2.1361797 | -0.526794932 |
| Akap6 | 4.4553313 | 2.1172369 | -0.524785755 |
| Olfr503 | 4.406626 | 2.0959167 | -0.524371549 |
| BC046401 | 4.5418205 | 2.163124 | -0.523731948 |
| 1700119I11Rik | 4.4879813 | 2.1412778 | -0.522886203 |
| Ntn5 | 4.505425 | 2.159052 | -0.520788383 |
| Olfr345 | 4.612158 | 2.2289402 | -0.516725099 |
| Havcr2 | 5.7137804 | 2.8394725 | -0.503048367 |
| Mtap7d2 | 5.7907515 | 2.8888016 | -0.501135284 |
| LOC100041223 | 4.17028 | 2.0948443 | -0.497672986 |
| 2900011O08Rik | 4.199082 | 2.1153882 | -0.496226032 |
| Prpf31 | 4.1288223 | 2.0810852 | -0.495961548 |
| Wnt8a | 4.149054 | 2.0961015 | -0.49480014 |
| Pf4 | 4.234008 | 2.1396523 | -0.49465086 |
| Mageb5 | 4.088491 | 2.0676548 | -0.494274342 |
| Ccl25 | 4.181516 | 2.1193867 | -0.493153512 |
| Vmn1r168 | 4.2043247 | 2.1324232 | -0.492802447 |
| Lcmt2 | 4.411225 | 2.244919 | -0.491089437 |
| Kpna7 | 4.259743 | 2.168992 | -0.49081623 |
| Glyctk | 4.4697533 | 2.2923183 | -0.48714881 |
| Gabra5 | 4.093771 | 2.1018913 | -0.486563538 |
| Cd55 | 4.247733 | 2.189571 | -0.484531867 |
| Gm364 | 4.239618 | 2.1910334 | -0.483200279 |
| Adamtsl3 | 4.5159636 | 2.3416538 | -0.48147195 |
| Gm13547 | 4.073487 | 2.1136172 | -0.481128281 |
| A430054B03 | 4.034481 | 2.0936093 | -0.481070973 |
| Sowahb | 5.0441766 | 2.6426914 | -0.476090627 |
| Olfr1462 | 4.118645 | 2.167129 | -0.473824765 |
| Mup5 | 4.085096 | 2.163012 | -0.470511342 |
| A330023F24Rik | 3.8923447 | 2.0690572 | -0.468429094 |
| Gm9325 | 7.303095 | 3.8855197 | -0.467962597 |
| Kcnd3 | 6.79689 | 3.6299984 | -0.465932449 |
| B4galnt3 | 3.9283855 | 2.104305 | -0.464333376 |
| Olfr448 | 3.9480047 | 2.1169739 | -0.463786378 |
| Lrp8 | 4.42931 | 2.3800352 | -0.462662311 |
| Kirrel3 | 3.8426025 | 2.0781515 | -0.459181245 |
| Tas2r117 | 5.3297343 | 2.8870974 | -0.458303691 |
| Dnahc10 | 3.9457304 | 2.1401813 | -0.457595658 |
| BC048671 | 5.086596 | 2.78796 | -0.451900642 |
| Tctex1d1 | 5.452116 | 3.003712 | -0.449074084 |
| Vmn2r98 | 6.6983566 | 3.694055 | -0.448513237 |
| Cd44 | 3.8335366 | 2.1211557 | -0.446684375 |
| Bcl2a1d | 3.8340144 | 2.1219738 | -0.44653995 |
| Hus1b | 3.918069 | 2.1707964 | -0.445952483 |
| Alox8 | 3.9176161 | 2.176947 | -0.444318447 |
| Gm2670 | 3.987846 | 2.217185 | -0.444014388 |
| Cbln3 | 6.252815 | 3.4831216 | -0.442951439 |
| Rabep1 | 3.8080394 | 2.126603 | -0.441549108 |
| Ppp1r17 | 4.3850474 | 2.4529395 | -0.440612774 |
| Gtdc1 | 5.7290435 | 3.2115402 | -0.439428205 |
| 1700014D04Rik | 5.369945 | 3.0351462 | -0.434790077 |
| Entpd3 | 3.8591979 | 2.1878676 | -0.433077117 |
| Gpat2 | 6.1075644 | 3.4826627 | -0.4297788 |
| Nlrp4e | 3.636526 | 2.0801113 | -0.427994933 |
| Olfr1161 | 3.9789093 | 2.2790322 | -0.427221877 |
| Pcdhb4 | 3.8626637 | 2.212523 | -0.427202788 |
| Gm10579 | 3.6035252 | 2.0681226 | -0.426083492 |
| Ern1 | 3.7139072 | 2.143411 | -0.422868994 |
| AI465300 | 5.735914 | 3.3295517 | -0.419525519 |
| Gm11783 | 3.833106 | 2.2295096 | -0.418354306 |
| H2-M9 | 3.7653387 | 2.1957927 | -0.416840589 |
| Jag2 | 3.5771368 | 2.0865557 | -0.4166967 |
| Gpr26 | 3.6293297 | 2.1227355 | -0.415116378 |
| Srcin1 | 3.657994 | 2.150536 | -0.412099637 |
| Vat1l | 3.8028202 | 2.2392228 | -0.411167849 |
| Serpinb3c | 3.545411 | 2.0896175 | -0.410613466 |
| 4930528H21Rik | 3.632256 | 2.1477199 | -0.408709105 |
| Olfr146 | 3.5766542 | 2.1174235 | -0.407987638 |
| C030017G13Rik | 6.2505717 | 3.7253206 | -0.404003221 |
| Gm12522 | 3.528911 | 2.1093483 | -0.402266507 |
| Gm1043 | 3.6312258 | 2.1736548 | -0.401399164 |
| Olfr181 | 3.5244634 | 2.1123433 | -0.400662438 |
| Rpl10l | 3.695322 | 2.2164216 | -0.400208805 |
| Olfr975 | 6.465602 | 3.8835049 | -0.399359116 |
| Rbm3 | 3.5063045 | 2.1068306 | -0.399130737 |
| Ctnna2 | 3.5914855 | 2.1657233 | -0.396983978 |
| Olfr804 | 3.6781287 | 2.2221158 | -0.395856975 |
| Ccdc8 | 3.6421256 | 2.2041395 | -0.394820569 |
| Dnajc6 | 3.460143 | 2.0953813 | -0.394423496 |
| Zfp853 | 3.8712857 | 2.3532715 | -0.39212146 |
| Chrnb2 | 3.413835 | 2.0854487 | -0.389118484 |
| Acsm3 | 5.4640894 | 3.3438356 | -0.388034244 |
| Bmp10 | 3.4150665 | 2.0937772 | -0.386900021 |
| Timm8a2 | 3.5041666 | 2.1503963 | -0.386331603 |
| Fam81b | 5.758379 | 3.5381577 | -0.385563594 |
| Gm534 | 3.4822392 | 2.1441512 | -0.384260794 |
| 0610012H03Rik | 3.3498535 | 2.0644617 | -0.383715825 |
| Zfp185 | 3.523439 | 2.172767 | -0.383339118 |
| Wdr65 | 3.6971414 | 2.2872605 | -0.381343516 |
| Ahsg | 3.6679893 | 2.2692502 | -0.381336745 |
| Hand1 | 4.2849674 | 2.653705 | -0.380694238 |
| 4930426I24Rik | 3.7310517 | 2.3115354 | -0.380460099 |
| Ticam2 | 3.8010352 | 2.3632135 | -0.378271083 |
| Cyb5rl | 5.7440596 | 3.5812159 | -0.376535734 |
| Gm10802 | 3.6274495 | 2.2671304 | -0.37500704 |
| F8a | 6.936492 | 4.338 | -0.374611835 |
| Tlr3 | 3.4314172 | 2.14614 | -0.374561624 |
| Calhm1 | 4.38077 | 2.740116 | -0.3745127 |
| Clcn6 | 7.261939 | 4.544016 | -0.374269599 |
| Olfr130 | 3.383409 | 2.1212213 | -0.373052061 |
| Gm7849 | 3.9399326 | 2.4781475 | -0.371017794 |
| 4932416K20Rik | 3.4134593 | 2.153185 | -0.369207361 |
| Cyp2c38 | 3.423481 | 2.164593 | -0.367721626 |
| A330033J07Rik | 3.2436934 | 2.0635893 | -0.363814934 |
| C030034L19Rik | 3.3303394 | 2.1284175 | -0.360900724 |
| Dmd | 3.4376242 | 2.1988955 | -0.36034442 |
| Car3 | 3.3409548 | 2.1375234 | -0.360205831 |
| Vmn1r56 | 3.3089879 | 2.1252508 | -0.357733886 |
| Mbd3l1 | 5.334397 | 3.4388099 | -0.355351711 |
| C8a | 3.2798629 | 2.118154 | -0.354194348 |
| Btnl1 | 3.3026605 | 2.1338239 | -0.353907585 |
| Olfr39 | 6.4883547 | 4.1960974 | -0.353287914 |
| Olfr140 | 3.2421663 | 2.096891 | -0.353243848 |
| Rdh13 | 5.6265993 | 3.6538558 | -0.35061027 |
| Kcna2 | 3.269738 | 2.1274602 | -0.349348419 |
| Bpifb2 | 3.1993878 | 2.0842218 | -0.348556058 |
| AI854517 | 3.2302816 | 2.1049504 | -0.348369381 |
| Ankrd33b | 4.6965966 | 3.0628479 | -0.347858 |
| 1190003K10Rik | 3.4031928 | 2.2212052 | -0.34731726 |
| Tmprss7 | 3.4761868 | 2.2766845 | -0.34506267 |
| Reg4 | 3.6096725 | 2.3646178 | -0.34492179 |
| St6galnac5 | 3.1937962 | 2.09345 | -0.344526116 |
| 4930554N03Rik | 3.2030501 | 2.1149032 | -0.339722098 |
| LOC100041550 | 3.876818 | 2.5627592 | -0.338952925 |
| 2810404F17Rik | 3.9580534 | 2.6208746 | -0.337837483 |
| Elf5 | 3.3910384 | 2.2520232 | -0.335889797 |
| Cela3b | 4.8282223 | 3.2116735 | -0.334812422 |
| BB287469 | 2.1526918 | 3.2468996 | 0.508297472 |
| Rp1l1 | 2.0778227 | 3.1395187 | 0.510965637 |
| Fgf8 | 2.2945633 | 3.4691901 | 0.511917366 |
| Pou2af1 | 2.7522168 | 4.1783743 | 0.518185014 |
| Gm44 | 2.9211938 | 4.454303 | 0.524822831 |
| Olfr827 | 2.0888608 | 3.1897774 | 0.52704163 |
| Fam47e | 2.248822 | 3.4574656 | 0.537456322 |
| Kcnh7 | 2.4951067 | 3.8730495 | 0.552258066 |
| Padi6 | 2.1045527 | 3.2674727 | 0.552573476 |
| Tnfaip8l3 | 2.2801516 | 3.556564 | 0.559792779 |
| Vps13b | 2.2548957 | 3.527296 | 0.564283439 |
| Olfr767 | 2.2505574 | 3.5291016 | 0.568101129 |
| 2310003L06Rik | 2.2881734 | 3.6037486 | 0.574945588 |
| Pdcd1 | 2.1960406 | 3.507061 | 0.596992788 |
| Zscan4c | 2.0820904 | 3.3433788 | 0.605779845 |
| Gm12052 | 2.413618 | 3.8833714 | 0.608942012 |
| Olfr1065 | 2.16364 | 3.481923 | 0.609289438 |
| B830008H07Rik | 2.3014624 | 3.7064757 | 0.610487184 |
| Gm10002 | 2.4146678 | 4.0035853 | 0.658027369 |
| Mei4 | 2.3319387 | 3.878684 | 0.66328729 |
| Olfr1431 | 2.1081872 | 3.512589 | 0.666165604 |
| Samd12 | 2.3432486 | 3.911162 | 0.669119529 |
| Olfr1322 | 2.3054862 | 3.8594565 | 0.674031491 |
| Pdia2 | 3.2647057 | 5.475571 | 0.677202022 |
| Olfr433 | 2.4939818 | 4.1908097 | 0.680368999 |
| D630028G08Rik | 2.2038186 | 3.7337322 | 0.694210313 |
| Txlng | 2.196222 | 3.7617688 | 0.712836316 |
| Adora3 | 2.1499732 | 3.6868412 | 0.714831236 |
| Rbm41 | 2.8973792 | 4.9718213 | 0.715971903 |
| 2210408I21Rik | 2.1102555 | 3.6273043 | 0.718893423 |
| Galnt13 | 3.7099242 | 6.3806086 | 0.719875732 |
| Zic4 | 2.4023445 | 4.1504154 | 0.72765205 |
| Olfr629 | 2.1171026 | 3.6804671 | 0.738445317 |
| Zfp369 | 2.6492 | 4.6141253 | 0.741705156 |
| Olfr476 | 2.2050493 | 3.8468966 | 0.744585302 |
| Spon1 | 2.1975265 | 3.8388572 | 0.746899161 |
| Ints6 | 3.6041982 | 6.335213 | 0.757731581 |
| Prrt3 | 2.1062489 | 3.7219577 | 0.767102502 |
| 1700009J07Rik | 2.1259675 | 3.7619479 | 0.76952277 |
| Mlc1 | 2.1946793 | 3.910051 | 0.78160472 |
| Ly9 | 2.3078728 | 4.1602693 | 0.802642373 |
| Gm7480 | 2.2149203 | 4.0063124 | 0.808784 |
| Olfr113 | 2.1112175 | 3.8460252 | 0.821709606 |
| Kcnq1ot1 | 2.2085295 | 4.05853 | 0.837661666 |
| 4930529I22Rik | 2.085304 | 3.862708 | 0.852347667 |
| Ctcfl | 2.134423 | 3.9689572 | 0.85949889 |
| Fut4 | 3.5308366 | 6.591389 | 0.866806581 |
| Uba6 | 2.5082812 | 4.7146535 | 0.879635146 |
| Olfr453 | 2.3424168 | 4.408478 | 0.882021167 |
| Ano5 | 2.1130176 | 4.02376 | 0.904271881 |
| Ccdc50 | 2.180384 | 4.169168 | 0.912125571 |
| Olfr592 | 2.095842 | 4.020919 | 0.918522007 |
| Gm9926 | 2.3376193 | 4.545578 | 0.944533055 |
| Sytl3 | 2.212039 | 4.3055873 | 0.94643372 |
| Lrrc48 | 2.219398 | 4.3414335 | 0.956131122 |
| B230307C23Rik | 2.0931494 | 4.103119 | 0.960260935 |
| Ccdc48 | 2.421556 | 4.757311 | 0.964567823 |
| Il7 | 2.2153368 | 4.3581705 | 0.9672722 |
| Efcab3 | 2.4088285 | 4.802515 | 0.993713957 |
| Lcorl | 2.1639042 | 4.4117913 | 1.038810822 |
| Nfasc | 2.6960902 | 5.548185 | 1.057863272 |
| Rfx4 | 2.2704115 | 4.721224 | 1.079457402 |
| Zfp677 | 3.1494436 | 6.6625175 | 1.115458584 |
| LOC100862611 | 2.527308 | 5.426284 | 1.147060825 |
| Gm6117 | 2.156596 | 4.7395144 | 1.197683015 |
| Gm14124 | 2.232318 | 4.984667 | 1.232955609 |
| Gm11826 | 2.2815976 | 5.136843 | 1.251423739 |
| Gm10554 | 2.6059313 | 5.9779577 | 1.293981311 |
| Mis18bp1 | 2.7923234 | 6.4437766 | 1.307675608 |
| C79562 | 2.2019753 | 5.1200767 | 1.325219861 |
| Phip | 2.0911555 | 5.258692 | 1.514730253 |
| Gm6712 | 2.4253793 | 6.154025 | 1.537345396 |
| LOC100044193 | 2.2830126 | 5.9980164 | 1.627237537 |
| 1700011E24Rik | 2.1424935 | 6.25449 | 1.919257398 |

(D) Genes up-regulated over one fold in TCs caused by the interaction of TCs and MSCs.

| GeneSymbol | TCs cocultured MSCs and stimulated with 1μg/ml LPS compared with TCs | TCs cocultured MSCs compared with TCs | TCs stimulated with 1μg/ml LPS compared with TCs |
| --- | --- | --- | --- |
| Rfx4 | 1.079457402 | 1.264867182 | 1.071069496 |

(E) Genes down-regulated over 0.5 fold in TCs caused by the interaction of TCs and MSCs.

| GeneSymbol | TCs cocultured MSCs and stimulated with 1μg/ml LPS compared with TCs | TCs cocultured MSCs compared with TCs | TCs stimulated with 1μg/ml LPS compared withTCs |
| --- | --- | --- | --- |
| 1110032F04Rik | -0.597199671 | -0.574327041 | -0.595810723 |
| 4930435F18Rik | -0.65650567 | -0.653395309 | -0.657934742 |
| 4930447N08Rik | -0.568251751 | -0.506125388 | -0.574481991 |
| 4933402N03Rik | -0.644510974 | -0.643350558 | -0.556360377 |
| A730089K16Rik | -0.58317325 | -0.5725708 | -0.582748398 |
| AI314760 | -0.534862365 | -0.530444257 | -0.536310221 |
| AI464131 | -0.715030275 | -0.715077416 | -0.652220172 |
| AI661453 | -0.529008095 | -0.622415833 | -0.626741089 |
| Akap6 | -0.524785755 | -0.520588626 | -0.52764754 |
| Alox15 | -0.623876035 | -0.620722283 | -0.625829528 |
| BC046401 | -0.523731948 | -0.5004746 | -0.524169879 |
| Cdx2 | -0.606040016 | -0.617025359 | -0.608365699 |
| Copg2as2 | -0.568429373 | -0.561088787 | -0.567610322 |
| D17Ertd648e | -0.593397211 | -0.602773314 | -0.59875909 |
| Dpep3 | -0.675800676 | -0.672913987 | -0.677541157 |
| Dusp15 | -0.537248115 | -0.536739688 | -0.535192076 |
| E130309F12Rik | -0.58303705 | -0.584659664 | -0.584284952 |
| Gm5463 | -0.655301311 | -0.669325988 | -0.674077535 |
| Heph | -0.557771788 | -0.554632075 | -0.559872452 |
| Hnf1a | -0.536185245 | -0.612543304 | -0.554195429 |
| Klra22 | -0.551458734 | -0.502634762 | -0.547229373 |
| LOC100505038 | -0.645224419 | -0.636082827 | -0.647435334 |
| Lrrc39 | -0.539426954 | -0.656276633 | -0.660589599 |
| Muc6 | -0.574160332 | -0.632824006 | -0.629768267 |
| Nlrp4a | -0.548874308 | -0.545225914 | -0.552010432 |
| Ntn5 | -0.520788383 | -0.50170894 | -0.52012354 |
| Olfr1335 | -0.621135892 | -0.622723889 | -0.622501801 |
| Olfr345 | -0.516725099 | -0.54146792 | -0.525491755 |
| Olfr45 | -0.584922923 | -0.544322479 | -0.584046657 |
| Olfr687 | -0.541408717 | -0.538382799 | -0.547807867 |
| Raver2 | -0.640921369 | -0.63905297 | -0.642054488 |
| Rdh1 | -0.579860091 | -0.567055629 | -0.517133746 |
| Slco6d1 | -0.633616267 | -0.631874383 | -0.633391577 |
| St8sia6 | -0.603503303 | -0.60013499 | -0.605356647 |
| Vmn1r200 | -0.531569213 | -0.518115064 | -0.538225869 |
| Vmn1r31 | -0.579551955 | -0.577359679 | -0.580648712 |
| Vmn1r45 | -0.566905639 | -0.625239593 | -0.629898392 |
| Wdr86 | -0.631333788 | -0.599209615 | -0.560296818 |
